# Genome-Wide Analysis of the Aquaporin Gene Family in Chickpea (*Cicer arietinum* L.)

**DOI:** 10.3389/fpls.2016.01802

**Published:** 2016-11-29

**Authors:** Amit A. Deokar, Bunyamin Tar'an

**Affiliations:** Department of Plant Sciences, Crop Development Centre, University of SaskatchewanSaskatoon, SK, Canada

**Keywords:** chickpea (*Cicer arietinum* L.), aquaporin gene family, biotic and abiotic stress, genome-wide characterization, gene structure, phylogeny

## Abstract

Aquaporins (AQPs) are essential membrane proteins that play critical role in the transport of water and many other solutes across cell membranes. In this study, a comprehensive genome-wide analysis identified 40 AQP genes in chickpea (*Cicer arietinum* L.). A complete overview of the chickpea AQP (CaAQP) gene family is presented, including their chromosomal locations, gene structure, phylogeny, gene duplication, conserved functional motifs, gene expression, and conserved promoter motifs. To understand AQP's evolution, a comparative analysis of chickpea AQPs with AQP orthologs from soybean, Medicago, common bean, and Arabidopsis was performed. The chickpea AQP genes were found on all of the chickpea chromosomes, except chromosome 7, with a maximum of six genes on chromosome 6, and a minimum of one gene on chromosome 5. Gene duplication analysis indicated that the expansion of chickpea AQP gene family might have been due to segmental and tandem duplications. CaAQPs were grouped into four subfamilies including 15 NOD26-like intrinsic proteins (NIPs), 13 tonoplast intrinsic proteins (TIPs), eight plasma membrane intrinsic proteins (PIPs), and four small basic intrinsic proteins (SIPs) based on sequence similarities and phylogenetic position. Gene structure analysis revealed a highly conserved exon-intron pattern within CaAQP subfamilies supporting the CaAQP family classification. Functional prediction based on conserved Ar/R selectivity filters, Froger's residues, and specificity-determining positions suggested wide differences in substrate specificity among the subfamilies of CaAQPs. Expression analysis of the AQP genes indicated that some of the genes are tissue-specific, whereas few other AQP genes showed differential expression in response to biotic and abiotic stresses. Promoter profiling of CaAQP genes for conserved *cis*-acting regulatory elements revealed enrichment of *cis*-elements involved in circadian control, light response, defense and stress responsiveness reflecting their varying pattern of gene expression and potential involvement in biotic and abiotic stress responses. The current study presents the first detailed genome-wide analysis of the AQP gene family in chickpea and provides valuable information for further functional analysis to infer the role of AQP in the adaptation of chickpea in diverse environmental conditions.

## Introduction

Chickpea (*Cicer arietinum* L.) is second most important food legume crop grown globally over 13.5 Mha with the production of 13.1 Mt in 2013 (FAOSTAT 2013: http://faostat.fao.org/site/339/default.aspx). The productivity of chickpea has been limited by several biotic and abiotic factors, among them drought is one of the major abiotic stress causing a significant reduction in yield in the majority of chickpea growing areas (Krishnamurthy et al., 1999). Soil salinity is another increasing abiotic stress in many of the chickpea growing areas (Flowers et al., 2010). Understanding the genetic and molecular mechanisms of tolerance to drought and salinity will help to improve the adaptation of chickpea to these adverse conditions. Both the drought and salinity stresses cause tissue dehydration instigated by the imbalance between root water uptake and leaf transpiration and modify root water uptake (Wahid and Close, 2007). Water is absorbed by plants through root hairs by apoplastic (passive absorption) and symplastic (active absorption) pathways (Suga et al., 2002; Lian et al., 2004). The later is more active under different abiotic stress conditions and mainly regulated by members of aquaporin family proteins (Amodeo et al., 1999). As a central part of water absorption and transportation, the role of aquaporin (AQP) genes in response to biotic and abiotic stresses has been reported in several plants (Jang et al., 2004; Alexandersson et al., 2005, 2010; Aroca et al., 2012; Zhou et al., 2012). Despite the importance of AQPs in regulating stress tolerance, very limited studies on understanding the role of AQPs in biotic and abiotic stresses were reported in chickpea. Differential regulation of some of the AQP genes under drought stress has been described (Molina et al., 2008; Deokar et al., 2011), suggesting the potential involvement of AQPs in drought and other osmotic related stresses in chickpea.

AQP are water channel proteins belong to the membrane intrinsic proteins (MIPs) family that facilitate the rapid and selective transport of water and several small molecules, such as glycerol, and urea, dissolved gasses such as carbon dioxide and ammonia, and metalloids such as boron and silicon across plant cell membranes (Chaumont et al., 2001; Kaldenhoff et al., 2008; Hachez and Chaumont, 2010; Maurel et al., 2015). Apart from the water transport, AQPs are also involved in different physiological processes such as seed longevity and seed viability (Mao and Sun, 2015), sexual reproduction/anther dehiscence (Bots et al., 2005), photosynthesis, and stomatal and mesophyll conductance (Perez-Martin et al., 2014), cell elongation (Yang and Cui, 2009), and responses to diverse biotic and abiotic stress treatments (Jang et al., 2004; Peng et al., 2007; Montalvo-Hernández et al., 2008; Aroca et al., 2012; Khan et al., 2015).

Based on their subcellular localization, the AQPs genes are classified into five subfamilies including, the tonoplast intrinsic proteins (TIPs), the plasma membrane intrinsic proteins (PIPs), the nodulin-like plasma membrane intrinsic proteins (NIPs), the small intrinsic proteins (SIPs) and the uncategorized (X) intrinsic proteins (XIPs) (Maurel et al., 2015). These AQP subfamilies were found highly conserved in higher plant species; however, the XIP subfamily is absent in monocots or the Brassicaceae (Deshmukh et al., 2015). Although the AQP were initially classified and named on the basis of their subcellular localization, recent studies demonstrated complex and highly regulated mechanism of AQP at subcellular localization, such as dual localization of ZmPIP1;2 in the PM and in the ER of root elongating cells (Chaumont et al., 2000) and salt stress condition which regulate Arabidopsis PIPs to relocated to intracellular vesicles (Boursiac et al., 2008).

The tonoplast AtTIP1;1 gene from Arabidopsis (Arabidopsis thaliana) was the first plant AQP protein characterized for water channel activity (Maurel et al., 1993). After that several plant AQP genes have been identified and characterized using Xenopus oocyte, yeast and or plant protoplast swelling assays for water movement (Kaldenhoff et al., 1998; Sommer et al., 2007, 2008; Gomes et al., 2009). With the availability of whole-genome sequences for several plant species, aquaporin-related sequences were identified using genome-wide analysis. In higher plants depending on the ploidy level, AQP family constitutes from 30 to more than 70 diverse members. 35 AQP homologs from Arabidopsis (Johanson et al., 2001; Quigley et al., 2002), 33 from rice (Sakurai et al., 2005; Nguyen et al., 2013), 31 from maize (Chaumont et al., 2001), 37 from tomato (Reuscher et al., 2013), 41 from common bean (Ariani and Gepts, 2015), 72 from soybean (Deshmukh et al., 2013), and 71 in cotton (Park et al., 2010) have been identified. Compared to other plant species, little is known about the AQP genes in chickpea, only a few differentially expressed EST sequences encoding the AQP protein has been identified (Molina et al., 2008; Jain and Chattopadhyay, 2010; Deokar et al., 2011).

The availability of whole genome sequence of chickpea would facilitate genome-wide analysis to identify the complete set of AQPs in chickpea, and comparative analysis to understand the evolutionary relationship of chickpea's AQPs with related plant species. The present study was carried out to identify chickpea AQP genes using genome-wide analysis and to characterize their phylogeny, chromosomal distribution, and structure. Additionally, we also investigated the expression profile of AQPs genes in various tissues and in response to biotic and abiotic stresses.

## Materials and methods

### Identification of putative aquaporin genes in chickpea genome

Nucleotide and protein sequences of chickpea genome (assembly ASM33114v1) were retrieved from NCBI and (CDC Frontier genome Cav1.0) from https://www.coolseasonfoodlegume.org/analysis/105. Nucleotide and protein sequences of Medicago (*Medicago truncatula*), soybean (*Glycine max*) and common bean (*Phaseolus vulgaris* L.) were retrieved from Phytozome database (http://genome.jgi.doe.gov/pages/dynamicOrganismDownload.jsf?organism=PhytozomeV11). The four species were selected based on their whole-genome sequence availability, their agricultural importance, as a model legume species and representatives of phaseoloid (common bean and soybean) and galegoid (chickpea and Medicago) the two major clades of papilionoideae (grain legume) family. A local nucleotide and protein database of annotated chickpea genes was created using NCBI command-line BLAST utilities in BioEdit (Version 7.0.9.0). The putative chickpea aquaporin genes were identified with BLASTp using 218 known aquaporin genes as query sequences against the chickpea local database. An e-value of 10^−5^ was used as an initial cut-off to claim significant matches. Then, the BLAST output was tabulated and top hits on the basis of bit scores were selected. BLAST hits with less than a 100 bit-score were removed.

### Multiple sequence alignments and phylogenetic analysis

The predicted Chickpea AQP genes (CaAQPs) were classified into subgroups based on sequence alignment and phylogenetic relationship with clearly classified AQPs from Arabidopsis (Quigley et al., 2002). Multiple sequence alignments of amino acid sequences of CaAQPs and those of Arabidopsis, Medicago, soybean, and common bean were performed using CLUSTALW implemented in MEGA5 software (Tamura et al., 2011). A phylogenetic tree was then constructed using maximum likelihood (ML) method. A bootstrap analysis with 1000 reiterations was conducted to determine the statistical stability of each node. The aquaporin subgroups PIP, TIP, NIP, SIP, and XIP formed in the phylogenetic tree were classified in accordance with the nomenclature of known AQPs that were used as a query in initial BLAST search. The phylogenetic tree was visualized using iTOL (http://itol.embl.de/help.cgi).

### Protein characterization and identification of NPA motifs and transmembrane domains in CaAQPs

Conserved domains within the CaAQP protein sequences were identified using NCBI's Conserved Domain Database (CDD, www.ncbi.nlm.nih.gov/Structure/cdd/cdd.shtml). Transmembrane domains were detected using TMHMM 2.0 (www.cbs.dtu.dk). All results were manually examined to re-confirm the CDD results. NPA motifs, ar/R filters (H2, H5, LE1, LE2), Froger's positions (P1–P5) and specificity-determining positions (SDP1-SDP9) were predicted based on careful visual inspection of multiple sequence alignments of CaAQPs with structure resolved and functionally characterized AQPs as reported earlier (Froger et al., 1998; Wallace and Roberts, 2004; Hove and Bhave, 2011; Zhang da et al., 2013).

The Isoelectric Point (pI), molecular weight and grand average of hydropathy (GRAVY) of the amino acid sequences were predicted by Sequence Manipulation Suite (SMS) V2 available at geneinfinity web server (http://www.geneinfinity.org/index.html?dp=5). The subcellular localization of CaAQPs was predicted using WoLF PSORT a protein Subcellular Localization Prediction Tool available at http://www.genscript.com/wolf-psort.html.

### Genomic organization and promoter sequence profiling of CaAQPs

The physical location of CaAQPs on each chickpea chromosome was detected using BLASTNT search against the local database of the CDC Frontier genome Cav1.0. Starting position of all CaAQP genes were used as the indicative position of the genes on the chromosome or the scaffold. MapChart was used to plot the chickpea chromosomes and the respective positions of CaAQPs (Voorrips, 2002).

Intron-exon structures of all CaAQPs were retrieved from the gene annotation file (GFF) of the CDC Frontier genome Cav1.0. Intron-exon structures of the genes were visualized using GSDS 2.0 server (http://gsds.cbi.pku.edu.cn/).

The core promoter sequence (1 kb upstream region from the predicted transcription start site) for all CaAQPs was extracted from CDC Frontier genome Cav1.0. The PlantCARE (http://bioinformatics.psb.ugent.be/webtools/plantcare/html/) database of plant cis-acting regulatory elements was used to find putative cis-acting regulatory elements in the CaAQP promoter sequences.

### 3D structure

The three-dimensional (3D) structure of CaAQPs were generated by intensive protein modeling using Phyre2 server (http://www.sbg.bio.ic.ac.uk/phyre2/html/page.cgi?id=index) using ‘Normal’ mode modeling based on alignment to experimentally solved protein structures. Transmembrane helix and topology of the CaAQPs were predicted by MEMSAT-SVM prediction method available in Phyre2 server.

### Expression analysis of CaAQPs

Over the past few years, the number of publicly available transcriptome (RNA-seq) datasets has greatly increased. These publicly available data sets are extremely useful for large-scale gene expression studies. RNA-seq data were downloaded from the NCBI Sequence Read Archive (SRA) database (http://www.ncbi.nlm.nih.gov/sra/) and used to analyze the expression pattern of the CaAQPs under different biotic and abiotic stresses. The following Sequence Read Archives were used for fusarium wilt stress (SRX535349, SRX535346, SRX535348, SRX535351, and SRX548566), root drought stress (SRX402840), shoot drought stress (SRX402844), root salinity stress (SRX402841), shoot salinity stress (SRX402845), root cold stress (SRX402842), shoot cold stress (SRX402846), root control (SRX402839) and shoot control (SRX402843). For the tissue-specific expression, RNA-seq data from various plant tissues and development stages including shoot (SRX402843), young leaves (SRX208031), apical meristem (SRX208032), flower (SRX208039, SRX208038, and SRX208037), flower bud (SRX208033, SRX208034, SRX208035, and SRX208036), young pod (SRX361953) and root (SRX402839) were used.

Gene expression of the CaAQP genes was presented as the FPKM (fragments per kilobase of transcript per million mapped reads) values. Hierarchical clustering of the CaAQP genes based on the expression data was performed using Cluster 3.0 software (http://bonsai.hgc.jp/~mdehoon/software/cluster/software.htm), using “correlation (uncentered)” as the distance metric and average linkage method. Clustering trees and Heatmaps was visualized using Java TreeView software (http://jtreeview.sourceforge.net/).

## Results and discussion

### Aquaporin gene family in chickpea

Whole-genome sequence availability of Kabuli cultivar CDC Frontier (Varshney et al., 2013) provided an opportunity to identify and analyse AQP genes in chickpea. We identified a total of 40 putative aquaporin encoding genes chickpea genome, namely, thereafter, as CaAQPs (Table 1 and Supplementary File S1). The number of AQP identified in this study are slightly higher than the 35 AQP genes reported in Arabidopsis (Johanson et al., 2001), 33 in rice (Sakurai et al., 2005), 35 in Medicago and 30 in lotus genome, then again almost same in number as reported 41 AQP genes in potato (Venkatesh et al., 2013), Sorghum (Reddy et al., 2015), common bean (Ariani and Gepts, 2015), and 40 AQP genes in Pigeonpea (Deshmukh et al., 2015).

**Table 1:** **Nomenclature and protein properties of chickpea aquaporins**.

**AQP sub-family**	**Proposed**	**Locus^a^**	**Chromosome location^a^**	**Protein Isoelectric Point**	**Protein Molecular Weight (kD)**	**GRAVY^b^**	**Predicted subcellular location^c^**
PIP	CaPIP1-1	Ca_02435	Ca8:1717936-1719735	9.1	30.75	0.396	plas
	CaPIP1-2	Ca_05754	Ca6:5464092-5465462	9	30.81	0.427	plas
	CaPIP1-3	Ca_10319	Ca6:1932039-1933176	8.68	30.92	0.398	plas
	CaPIP1-4	Ca_12502	Ca2:30479427-30481282	8.7	31.06	0.393	plas
	CaPIP2-5	Ca_04707	Ca5:30303896-30304911	9.14	30.21	0.49	cyto
	CaPIP2-1	Ca_08491	Ca4:10225765-10228793	8.34	30.52	0.588	plas
	CaPIP2-2	Ca_12039	Ca3:32991462-32992568	9.11	30.8	0.406	plas
	CaPIP2-3	Ca_14568	Ca6:27966413-27967838	6.71	30.58	0.375	plas
	CaPIP2-4	Ca_02533	Ca1:12226605-12227713	7.81	30.87	0.382	plas
TIP	CaTIP1-1	Ca_00723	Ca3:34451205-34452295	6.02	25.47	0.88	vacu
	CaTIP1-2	Ca_16712	Ca6:26818668-26820154	5.43	25.82	0.847	vacu
	CaTIP1-3	Ca_18630	Ca4:17906721-17908388	5.61	26.01	0.78	plas
	CaTIP1-4	Ca_19377	Ca3:11393179-11394299	4.94	25.79	0.744	cyto
	CaTIP2-1	Ca_02797	Ca1:9896268-9897261	6.29	25.42	0.745	nucl
	CaTIP2-2	Ca_24137	scaffold1844:60282-61675	5.3	25.32	0.873	vacu
	CaTIP2-3	Ca_02338	Ca8:2481906-2483425	4.8	25.22	0.947	vacu
	CaTIP3-1	Ca_03854	Ca4:3990175-3991038	7.31	27.59	0.565	cyto
	CaTIP3-2	Ca_19737	Ca8:10865608-10867961	6.36	26.92	0.671	mito
	CaTIP4-1	Ca_14915	Ca4:39954258-39957667	7.92	23.85	0.893	cyto
	CaTIP4-2	Ca_14916	Ca4:39950985-39952627	5.51	25.87	0.777	vacu
	CaTIP5-1	Ca_15805	Ca6:34791986-34793660	7.08	26.49	0.731	vacu
NIP	CaNIP1-1	Ca_06493	Ca6:18836478-18837512	5.15	26.59	0.732	plas
	CaNIP1-2	Ca_16129	Ca2:29576437-29578961	7.06	28.53	0.632	plas
	CaNIP1-3	Ca_08631	Ca6:9654827-9656732	8	28.67	0.522	vacu
	CaNIP1-4	Ca_08632	Ca6:9651625-9653530	8	28.67	0.522	vacu
	CaNIP1-5	Ca_08630	Ca6:9661095-9662900	9.15	29.44	0.504	cyto
	CaNIP1-6	Ca_00434	Ca1:3567423-3568768	6.46	27.09	0.761	plas
	CaNIP1-7	Ca_00435	Ca1:3574718-3576622	8.98	29.17	0.421	plas
	CaNIP1-8	Ca_00436	Ca1:3579756-3581062	5.73	25.65	0.822	plas
	CaNIP1-9	Ca_00437	Ca1:3583849-3585825	6.31	29.8	0.501	plas
	CaNIP2-1	Ca_21333	Ca3:3854949-3859331	8.81	28.65	0.354	plas
	CaNIP3-1	Ca_02921	Ca1:8870515-8872730	9.09	31.93	0.361	plas
	CaNIP3-2	Ca_22848	Ca5:17858736-17862203	6.47	26.55	0.713	plas
	CaNIP3-3	Ca_25553	scaffold590:19540-20529	5.44	21.78	0.773	plas
	CaNIP3-4	Ca_04355	Ca4:11227522-11231177	8.56	30.7	0.502	plas
	CaNIP4-1	Ca_07775	Ca4:1463846-1467215	8.72	36.17	0.504	plas
	CaNIP6-1	Ca_22925	Ca6:53303745-53307875	8.49	25.56	0.228	vacu
SIP	CaSIP1-2	Ca_19143	Ca2:6911037-6913006	6.3	22.93	0.979	vacu
	CaSIP1-1	Ca_08262	Ca3:26454060-26457001	8.89	26.44	0.697	vacu
	CaSIP2-1	Ca_08136	Ca3:27710934-27713756	9.43	26.11	0.603	vacu

a *Gene IDs and AQP location are based on CDC Frontier genome assembly v1.0*.

b *GRAVY, Grand Average of Hydropathy*.

c *Predicted subcellular location of CaAQPs: plas, plasma membrane; cyto, cytosol; vacu, vacuolar; nucl, nuclear; mito, mitochondria*.

**Figure 1:**
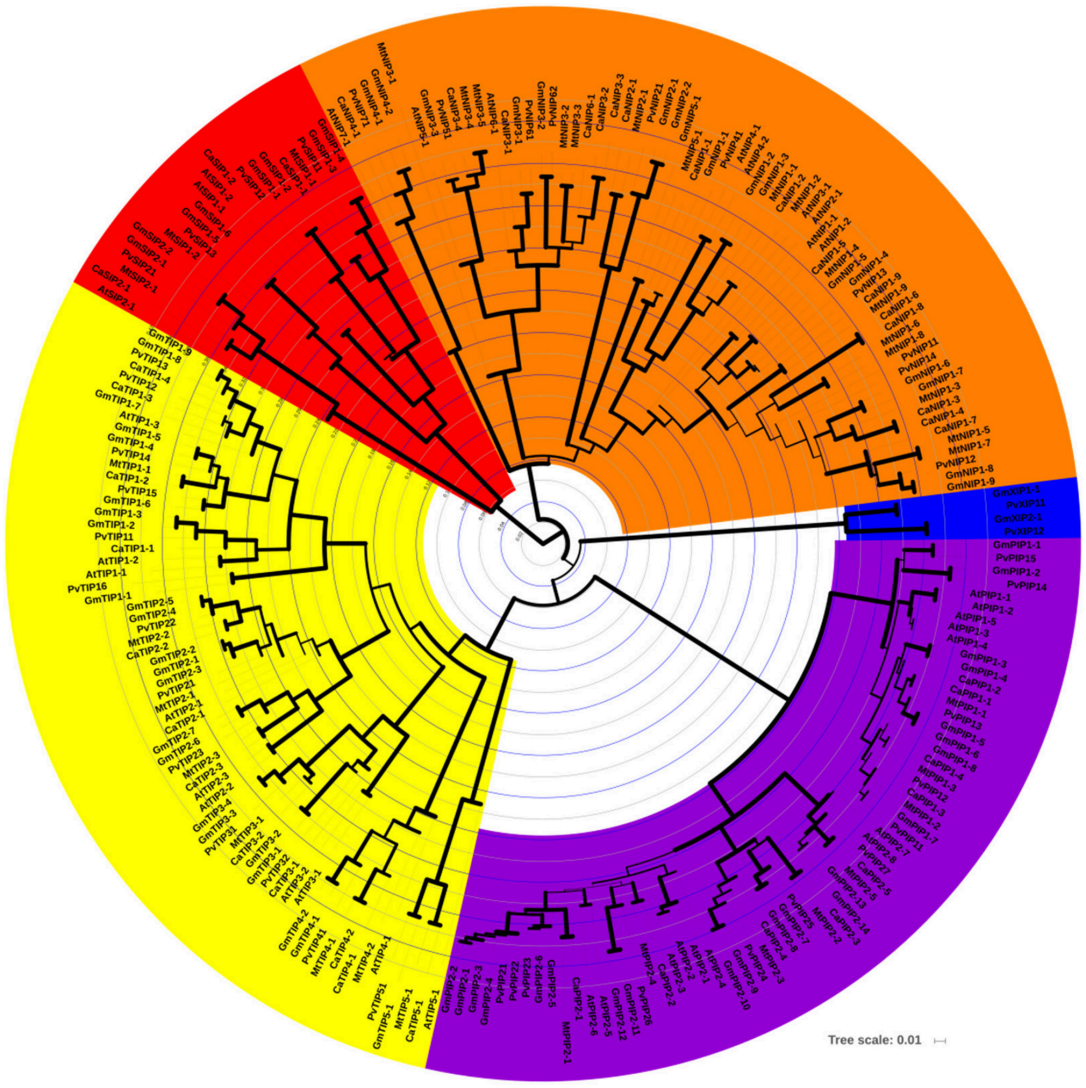
**Phylogenetic relationships among the chickpea, soybean, Medicago, common bean, and Arabidopsis AQP genes**. The phylogenetic tree is NJ tree and bootstrap support is based on 1000 replicates. All five subfamilies of AQP gene family are well separated in different clades and represented by different color background. TIP subfamily clade is represented by yellow color, NIPs by orange, SIPs by red, PIP by purple, and XIP by blue color. Bootstrap values are symbolized as tree branch width. The minimum bootstrap value represented as a tree branch width of 1 pixel width, whereas max bootstrap value represented by a tree branch width of 10 pixels.

A strong correlation between phylogenetic analysis with the gene function has been observed in AQPs (Soto et al., 2012; Perez Di Giorgio et al., 2014), indicating that the amino acid based phylogenetic analysis can be used to predict a putative function of the identified CaAQPs. To investigate the phylogenetic relationships and to predict the functionality of the CaAQPs, we constructed a phylogenetic tree of 223 protein sequences of aquaporin genes from four legumes including Medicago (35), common bean (41), soybean (72), chickpea (40), and 35 sequences from Arabidopsis (Figure 1). In accordance with the AQP gene classification in Arabidopsis, the phylogenetic tree was subdivided into five clades with well-supported bootstrap values, representing five distinct AQPs subfamilies. The chickpea AQPs identified in the present study were named according to nomenclature proposed in gene classification of Arabidopsis AQP genes as genus (one letter), species (one letter) i.e., Ca (*Cicer arietinum* L.), gene name (three letter code for AQP subfamily; e.g., PIP) (Table 1). All groups contain CaAQP genes, except the XIP group. The XIP group contains two genes from soybean and common bean. In addition to these two members of phaseoloid clade of Papilionoideae family, XIPs have been also reported in other phaseoloids such as pigeon pea (*Cajanus cajan*) (Deshmukh and Bélanger, 2016). However, the XIPs were completely missing or have not yet been reported in species from a galegoid clade of papilionoideae family such as Medicago and *Lotus japonicus* (Deshmukh and Bélanger, 2016). This observation suggests the possibility that the XIPs genes were mainly observed in phaseoloids clade (warm season legumes) and lost from the galegoid species (cool-season legumes) during the evolution of the papilionoideae family. Compared to the 40 CaAQPs, its legume counterparts Medicago, common bean, and soybean have 35, 41, and 72 AQP genes, respectively (Supplementary File S2). Multiple copies of AQP genes in soybean could be due to whole-genome duplication (WGD) event occurred twice at approximately 59 and 13 million years ago (mya) (Schmutz et al., 2010). Whole-genome sequence analysis of chickpea also observed a historical genome duplication after the divergence of legumes from Arabidopsis and grape (Varshney et al., 2013). The soybean whole-genome duplication events also occurred in the same period. The observed CaAQPs (40) number is within the range of Medicago, common bean, and Arabidopsis, but fewer than that of soybean. This could be due to the combination of gene loss and duplication events occurred during chickpea genome evolution.

**Figure 2:**
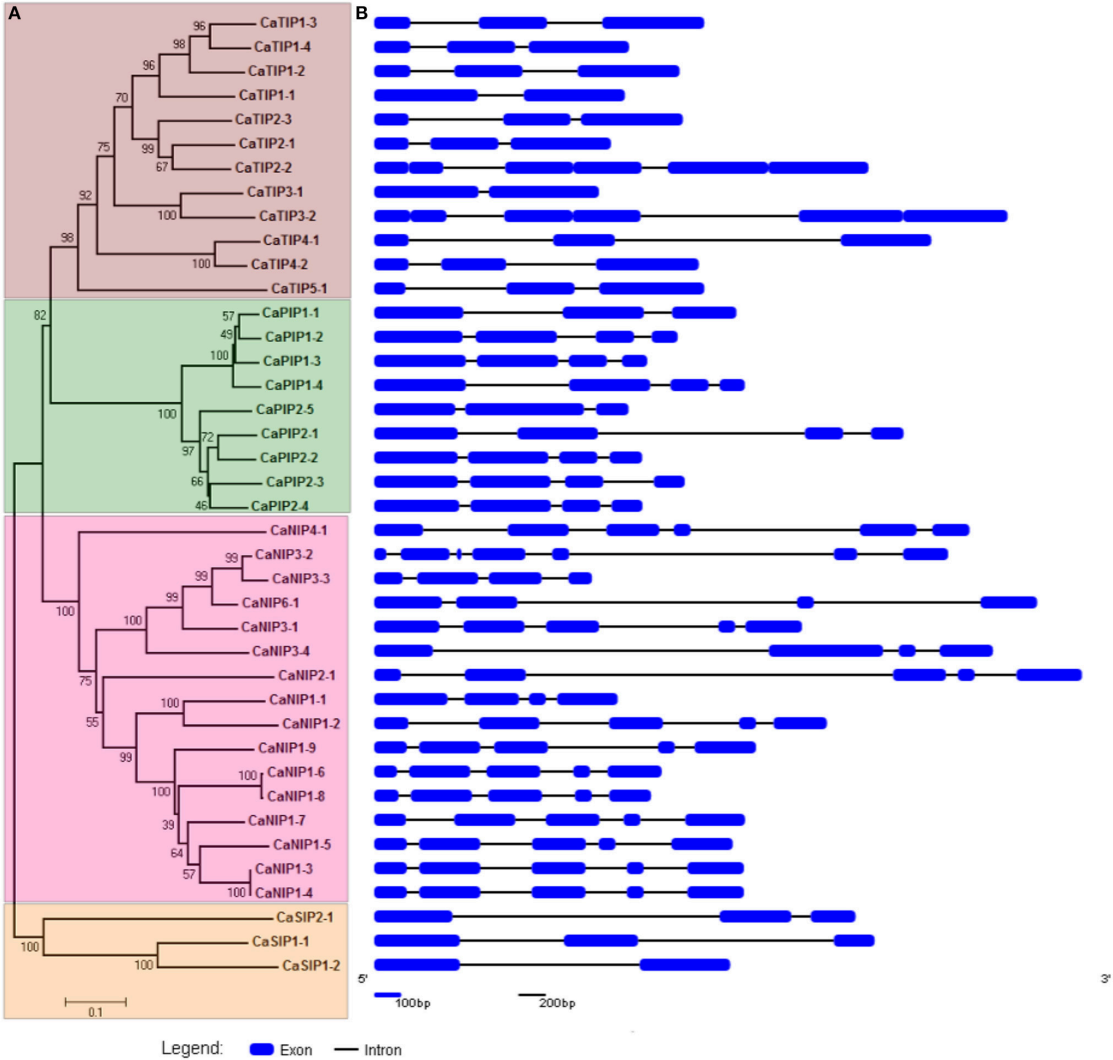
**Phylogenetic relationship and structural analysis of CaAQP genes. (A)** A Neighbor-joining evolutionary tree of 40 CaAQP genes was created with 1000 bootstraps. The 40 CaAQPs clustered into groups representing four AQP subfamilies CaTIPs, CaPIPs, CaNIPS, and CaNIPs. **(B)** Exons and introns of 40 CaAQP genes are represented by blue boxes and black lines, respectively. Gene models are based on CDC Frontier genome Cav1.0 gene annotations.

The CaAQPs were classified in four different subfamilies, PIPs (9 members), TIPs (12), NIPs (16 members), and SIPs (3 members). Further, the subfamilies were sub-grouped based on the analysis of the aromatic/arginine (Ar/R) selective filters and phylogenetic positioning that corroborate each other very well. The CaPIP subfamily was grouped in two subgroups CaPIP1 and CaPIP2 with four and five members in each group, respectively. Similarly, the CaSIP subfamily was also grouped in two subgroups CaSIP1 and CaSIP2 with two and one members in each group, respectively. The CaTIP subfamily was clustered in five subgroups (CaTIPs1-5). The largest subfamily CaNIPs consisted of six subgroups (CaNIPs1-6), with a maximum of nine members in CaNIP1 subgroups (Figure 2A and Table 1). Among the CaAQPs, CaNIPs were the most diverse subfamily with 47.5% sequence similarity, whereas CaPIPs were the most conserved subfamily with 76.1% sequence similarity at the amino acid level (Supplementary Table 1).

Plant AQPs have been shown to have different biochemical properties associated with the function characteristics (Johanson and Gustavsson, 2002; Hove and Bhave, 2011). In order to analyse biochemical properties of the identified genes, we predicted molecular weight, protein Isoelectric Point (pI) and predated subcellular localization of the CaAQPs. The identified CaAQPs encodes protein ranging from 251 to 335 (average 261) amino acids in length, a molecular weight ranging from 21.8 to 36.2 (average 27.9) kD and a pI value ranging from 4.8 to 9.43 (average 7.4) (Table 1). Among the CaAQP subfamilies, the average pI value of CaTIPs (pH 6.0) is less (i.e., more acidic) than the TIPs (pH 8.5) i.e., more basic (Table 1). The difference is mainly due to the presence or absence of basic residues in the C-terminal domains of AQP proteins as also observed in Arabidopsis and sweet orange AQPs (Johansson et al., 2000; Martins Cde et al., 2015). A positive grand average of hydropathy (GRAVY) scores of all the identified CaAQP proteins indicated the hydrophobic nature of the CaAQP proteins, which is a key property of aquaporins which facilitates the high water permeability (Murata et al., 2000). Further, among the CaAQPs subfamilies, the PIPs have the lowest average of GRAVY value (0.43) indicating better interaction of CaTIPs with water molecules.

The analysis of the predicted subcellular localization of the CaAQPs showed that all CaPIPs, except CaPIP2-5, are located at the plasma membrane. The CaPIP2-5 has been predicted to localize at cytosol (Table 1). Similarly, 12 out of 16 CaNIPs were also predicted to be localized to the plasma membrane. It has been shown that the majority of plants PIPs and some NIPs were preferentially localized at the plasma membrane (Maurel et al., 2008; Xu et al., 2014), however, this is not a general feature of TIPs or PIPs. Some of the Tobacco NtAQP1, a member of PIP group was found in the inner chloroplast membrane and plasma membrane (Uehlein et al., 2008). Similarly, dual localizations of maize PIP gene (ZmPIP1;2) in the plasma membrane and endoplasmic reticulum (ER) have also been observed (Chaumont et al., 2000). The seed specific isoforms of Arabidopsis TIP genes (AtTIP3;1 and AtTIP3;2) were also found in tonoplast and plasma membrane (Gattolin et al., 2011). However, molecular mechanisms underlying the dual localizations of these genes are not yet clear (Luu and Maurel, 2013). With the increasing number of functionally characterized plant aquaporin genes, more diverse patterns of subcellular localization and relocalization or redistribution AQPs that were influenced by environmental conditions such as drought and salinity have been also reported (Boursiac et al., 2008; Luu et al., 2012). All these observations indicated that the AQP sub-cellular localization is complex and highly regulated.

### Gene structure and genomic distribution of CaAQP

The gene structure analysis showed the distribution of introns and exons in the CaAQP genes, from one to six introns per genes (Figure 2B). Variation in the average number of introns between the CaAQP subfamily was also observed. The members of SIPs and TIPs subfamilies contained a maximum of 2 introns, with an average of 1.7 and 2.0 introns per gene, respectively, whereas the members of PIPs and NIP's subfamily contains a maximum of 4 and 6 introns, with an average of 2.9 and 4 introns per gene. Gene length variation within CaAQP was observed, where CaTIP3-1 (863 bp) was the shortest and CaNIP2-1 (4382 bp) was the longest AQP in chickpea genome. The length variation in intron size was also observed with the shortest intron size of 76 bp in CaNIP3-1 and CaTIP2-3 genes, whereas the largest intron size of 3118 bp in the CaNIP2-1 gene. With some exceptions, the exon-intron structure is conserved within each AQP subfamily. For example, the CaPIP subfamily contains three introns, except CaPIP1-1and CaPIP2-5, which contain two introns. Similarly, the majority of the CaTIP subfamily genes contains two introns with the exception of CaTIP1-1 and CaTIP3-1 which contain one intron. Relatively diverse exon-intron pattern was observed for the CaNIPs subfamily, the majority (10 out of 16 CaNIPs) of the genes contains four introns, four NIPs (CaNIP1-1, CaNIP3-3, CaNIP3-4, CaNIP6-1) contain three introns, CaNIP4-1 contains one intron, and CaNIP3-2 contains six introns. Three members of the CaSIP subfamily contain two introns (CaSIP and CaSIP) and one (CaSIP) intron. Similar exon-intron patterns have been reported in PIP and TIP subfamily of Arabidopsis, soybean, tomato, and orange (Johanson et al., 2001; Deshmukh et al., 2013; Reuscher et al., 2013; Martins Cde et al., 2015). Overall, the gene structure analysis indicated a conserved pattern of intron-exon within the subfamilies of CaAQPs suggesting the conserved evolution and supporting the CaAQP family classification.

**Figure 3:**
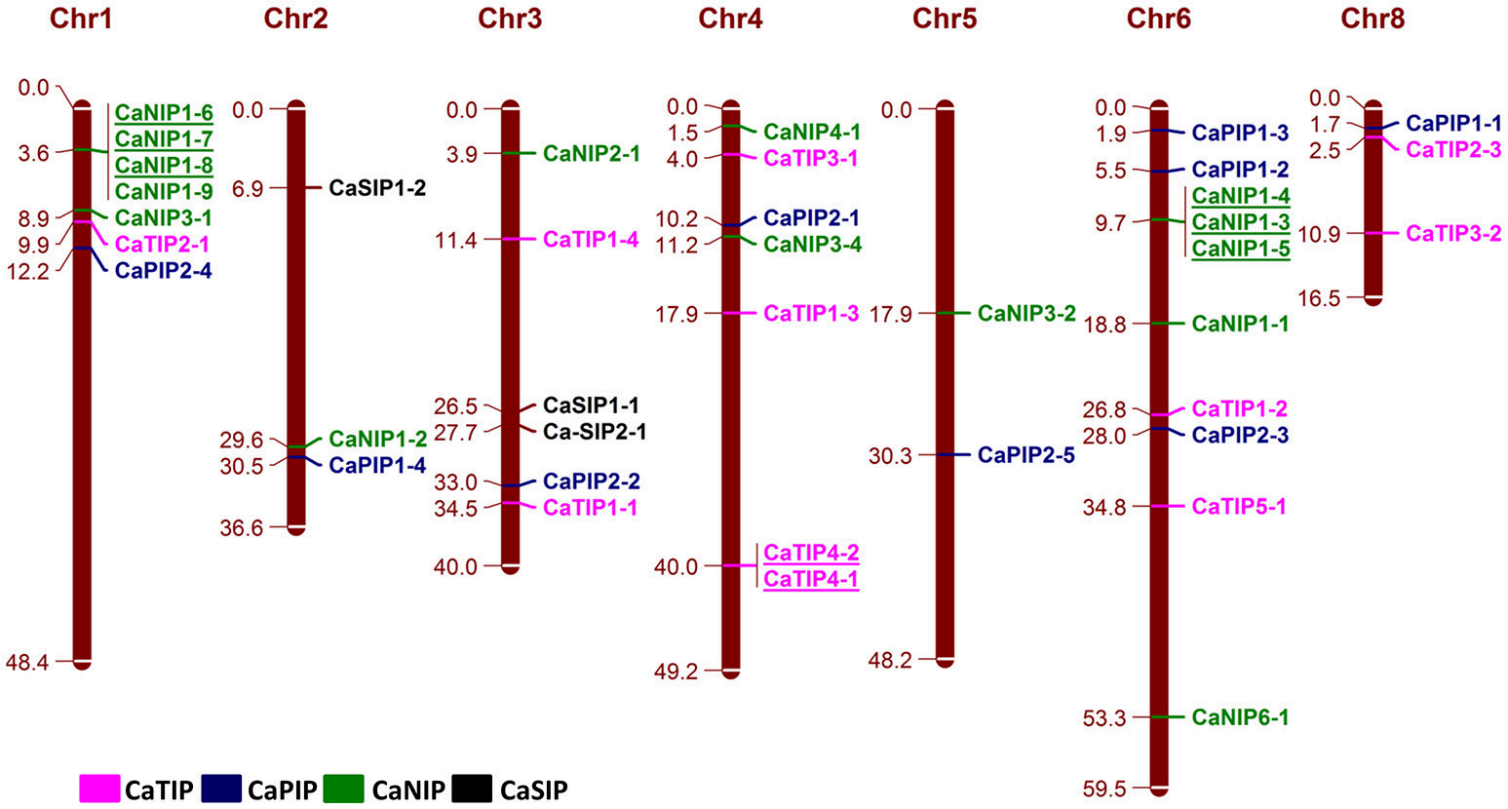
**Genomic distribution of CaAQP genes**. 38 CaAQP genes are located on all eight chickpea chromosomes, except chromosome 7. The remaining two CaAQP genes are located on scaffolds and not shown in this figure. The numbers at the right side of the chromosomal bar indicate the names of CaAQP genes and the corresponding position on the chromosome (megabase pairs; Mb) are given in left side. The “CaTIP” genes are represented in pink, “CaPIP” genes are in blue, “CaNIP” genes are in green and “CaSIP” genes are in black color. Tandemly duplicated genes are underlined.

The Physical mapping of the identified CaAQPs on the CDC Frontier kabuli genome assembly indicated the diverse distribution of the genes on chickpea chromosomes, except chromosome seven (Figure 3). Chromosome 3 contained the largest number (10; 25%) of the CaAQP genes, followed by chromosome 1 and 4, which both contained six members (17.5%). Chromosome 5 contained only two members (5.0%). Two CaAQP genes (CaTIP2-2 and CaNIP3-3) were located on unplaced scaffold sequences scaffold1844 and scaffold590, respectively. AQP gene density varies on individual chromosomes. The entire chickpea genome has an average of one AQP gene per 13.3 Mb. Further, some of the AQP genes were located in clusters at certain chromosomal regions, especially in the chromosome 1 and 6, and were dispersed in a single manner at other locations. Eight CaAQPs were found to be tandem duplications according to the criteria described in MCScan algorithm implemented in MCScanX software (Wang Y. et al., 2012). These genes included CaNIP1-6, CaNIP1-7, and CaNIP1-8 located on chromosome 1, CaTIP4-2, and CaTIP4-1 located on chromosome 4 and CaNIP1-3, CaNIP1-4, CaNIP1-5 located on chromosome 6. Additionally, 14 CaAQPs were predicted as segmentally or whole-genome duplication (WGD). Fifty percent of CaNIP subfamily members were predicted as either tandem or segmentally duplicated. These results suggested that the larger size of the NIP subfamily in legumes compared to Arabidopsis (16 members in chickpea and Medicago vs. nine members in Arabidopsis) may have evolved from gene duplication events. Expansion of AQP gene families via genome duplication events have been reported in other plants (Abascal et al., 2014). whole-genome sequence analysis also demonstrated that the significant number (69%) of the annotated chickpea genes have a history of gene duplication after the divergence of the legumes from Arabidopsis and grape (Varshney et al., 2013), which also supports our observation of CaAQPs gene duplication in chickpea.

### The conserved and substrate-specific residues of CaAQPs

Several conserved amino acid domains determining substrate specificity by affecting pore diameter and hydrophobicity have been reported in AQP family members (Froger et al., 1998; Hove and Bhave, 2011). The highly conserved features included six transmembrane α helices and two segments responsible for selectivity, the NPA domain (asparagine-proline-alanine) and the ar/R (aromatic arginine) selectivity filter (Kosinska Eriksson et al., 2013; Almasalmeh et al., 2014). Following a conserved domain database (CDD) BLAST search and careful visual inspection of amino acid sequence alignment, we identified conserved NPA domains, an ar/R selectivity filter, and Froger's residues in all 40 CaAQPs (Table 2, Supplementary File S3). The CaAQP fold is characterized by six transmembrane α-helices that are arranged in a right-handed bundle and five inter-helical loop regions (A-E) that form the extracellular and cytoplasmic vestibules. The majority of CaAQP (32 out of 40) showed six predicted transmembrane domains (TMDs), whereas the remaining eight CaAQPs have seven (CaNIP3-1, and CaTIP5-1), five (CaPIP2-1, CaPIP2-2, and CaSIP1-1) and four (CaNIP3-3, CaNIP6-1, and CaSIP2-1) TMDs. Two of the five interhelical loops, the loop B (LB) and E (LE) contain the highly conserved asparagine-proline-alanine (NPA) motifs that form one of the two major channel construction sites, the NPA region. Most of the identified putative CaAQPs also contain two typical NPA domains each one in loop B and loop E. However, six CaNIP and three CaSIP showed variable third residue of NPA motif (LB or LE) in which A is replaced by either L/S/T/V. Several studies also showed that the incompletely conserved NPA motifs also function as water channels (Johanson and Gustavsson, 2002; Yakata et al., 2007). The spacing between the two NPA motifs found essential for the silicon permeability by NIP2s in all silicon transporting plants (Deshmukh et al., 2015). We also observed diversity in the spacing between two NPA motif in CaAQPs ranging from 108 to 128 amino acids. Although silicon transportation mechanism in chickpea is unknown, it could be possible that the variation in NPA-NPA motif might be associated with permeability and transport of some unknown uncharged solutes.

**Table 2:** **Amino acid composition of the NPA motifs and ar/R selectivity filter of CaAQPs**.

**Gene_Id**	**NPA(LB)**	**NPA(LE)**	**Ar/R filters**				**Froger's residues**				
			**H2**	**H5**	**LE1**	**LE2**	**P1**	**P2**	**P3**	**P4**	**P5**
CaNIP1-1	NPA	NPA	W	V	A	R	F	S	A	Y	I
CaNIP1-2	NPA	NPA	W	L	A	R	F	S	A	Y	I
CaNIP1-3	NPA	NPA	W	V	A	R	F	S	A	Y	L
CaNIP1-4	NPA	NPA	W	V	A	R	F	S	A	Y	L
CaNIP1-5	NPA	NPA	W	A	A	R	F	S	A	Y	L
CaNIP1-6	NPA	NPA	W	V	A	R	F	S	A	Y	L
CaNIP1-7	NPA	NPA	W	V	A	R	F	S	A	Y	I
CaNIP1-8	NPA	NPA	W	V	A	R	F	S	A	Y	L
CaNIP1-9	NPA	NPA	W	I	A	R	F	S	A	Y	L
CaNIP2-1	NPA	NPA	G	S	G	R	L	T	A	Y	M
CaNIP3-1	NPA	NPV	S	I	G	R	F	S	A	Y	L
CaNIP3-2	NPA	NPV	S	I	G	R	F	A	A	Y	L
CaNIP3-3	NPA	–	S	I	–	–	F	–	–	–	–
CaNIP3-4	NPS	NPV	A	I	G	R	Y	T	A	Y	L
CaNIP4-1	NPA	NPA	A	V	G	R	Y	S	A	Y	I
CaNIP6-1	NPA	NPV	S	I	G	R	–	T	T	Y	L
CaPIP1-1	NPA	NPA	F	H	T	R	E	S	A	F	W
CaPIP1-2	NPA	NPA	F	H	T	R	E	S	A	F	W
CaPIP1-3	NPA	NPA	F	H	T	R	E	S	A	F	W
CaPIP1-4	NPA	NPA	F	H	T	R	E	S	A	F	W
CaPIP2-1	NPA	NPA	F	H	T	R	Q	S	A	F	W
CaPIP2-2	NPA	NPA	F	H	T	R	Q	S	A	F	W
CaPIP2-3	NPA	NPA	F	H	T	R	Q	S	A	F	W
CaPIP2-4	NPA	NPA	F	H	T	R	Q	S	A	Y	W
CaPIP2-5	NPA	NPA	F	H	T	R	M	S	A	F	W
CaSIP1-1	NPT	NPA	L	V	P	N	I	A	A	Y	W
CaSIP1-2	NPT	NPV	L	I	P	L	I	V	V	–	–
CaSIP2-1	NPL	NPA	F	H	G	A	I	V	A	Y	W
CaTIP1-1	NPA	NPA	H	I	A	V	T	S	A	Y	W
CaTIP1-2	NPA	NPA	H	I	A	V	T	S	A	Y	W
CaTIP1-3	NPA	NPA	H	I	A	V	T	S	A	Y	W
CaTIP1-4	NPA	NPA	H	I	A	V	T	S	A	Y	W
CaTIP2-1	NPA	NPA	H	I	G	R	T	S	A	Y	W
CaTIP2-2	NPA	NPA	H	I	G	R	T	S	A	Y	W
CaTIP2-3	NPA	NPA	H	I	G	R	T	S	A	Y	W
CaTIP3-1	NPA	NPA	H	I	A	R	T	A	A	Y	W
CaTIP3-2	NPA	NPA	H	I	A	L	T	A	S	F	W
CaTIP4-1	NPA	NPA	Q	I	A	R	S	S	A	Y	W
CaTIP4-2	NPA	NPA	H	I	A	R	S	S	A	Y	W
CaTIP5-1	NPA	NPA	N	V	G	C	L	A	A	Y	W

The second channel construction site known as “ar/R” that consist of tetrad formed by helicase 2 (H2) and 5 (H5) and two LE1 and LE2 residues from loop E. Different combination of ar/R selectivity filter in CaAQP family and a conserved pattern within the subfamilies were also observed. For example, in the CaPIP subfamily, all the members showed a conserved ar/R filter configuration (F-H-R-T), which was mainly observed in a water transporting AQP, indicating that the CaPIP family members are able to facilitate water and solute transport (Savage et al., 2003). Beside the NPA motif and ar/R selectivity filter, we also analyzed Froger's position P1-P5 the five conserved amino acid residues which discriminated glycerol-transporting aquaglyceroporins (GLPs) from water-conducting AQPs (Froger et al., 1998). The P2, P3, P4 and P5 Froger's positions in the CaPIPs were highly conserved (S-A-F-W), whereas the P1 position varies with either E/Q/M amino acid residue. Froger's positions in the CaNIP subfamily were highly variable in comparison to other subfamilies; however, groups within subfamily showed a conserved pattern. The CaNIP1 group has conserved ar/R filter W-[A/V/L]-A-R and F-S-A-Y-I residue in the P1-P5 positions, which has been predicted as conserved motifs in urea and H_2_O_2_ transporter NIPs (Hove and Bhave, 2011). The CaNIP3 group have conserved residue A-Y-L in the P2-P5 position, whereas the position P1 (F/Y) and P2 (S/A/T) having variable residues. In the subfamily CaTIP (CaTIP1 and CaTIP2) the ar/R filter H-I-A-V or H-I-G-R and the Froger's positions T-S-A-Y-W residue in the P1-P5 positions were highly conserved and were reported to involve in the transport of urea and H_2_O_2_ (Hove and Bhave, 2011).

Functional characterization of several AQP genes has shown that the roles of some members of AQP family are not limited to water transport, but also involved in the transport of non-aqua substrates, such as glycerol, CO_2_, NH_4_ +/NH_3_, urea, silicon, H_2_O_2_, arsenic, antimony, lactic acid, and boron (Rivers et al., 1997; Biela et al., 1999; Ma et al., 2006, 2008; Takano et al., 2006; Bienert et al., 2008). Based on sequence similarity of the functionally characterized plant AQP genes, Hove and Bhave (Hove and Bhave, 2011) proposed distinct signature sequences and nine specificity-determining positions (SDPs) for each substrate group. To postulate the role of CaAQPs in the transport of non-aqua substrates, we analyzed these conserved SDPs in CaAQPs (Supplementary File S4). The members of CaPIP1 and CaPIP2 showed the presence of SDPs deciphered for boric acid (T-I-H-P-E-L/M-L-T-P),CO_2_ (ILV-I/M-C-A-I/V-D/H-W) and H_2_O_2_ (A-G-V-F/L-I-Q/H-F/Y-V-P) transporter. On the other hand, all the CaPIPs and CaTIPs exhibited the H_2_O_2_ (A/S-G/A-V/L-F/L/A/I-I/V-Q/H/I/L-F/Y-V/A-P) and Urea (H-P-F-F/I/L-L-P/A-G-G/S-N) SDPs.

### Molecular modeling of CaAQP

**Figure 4:**
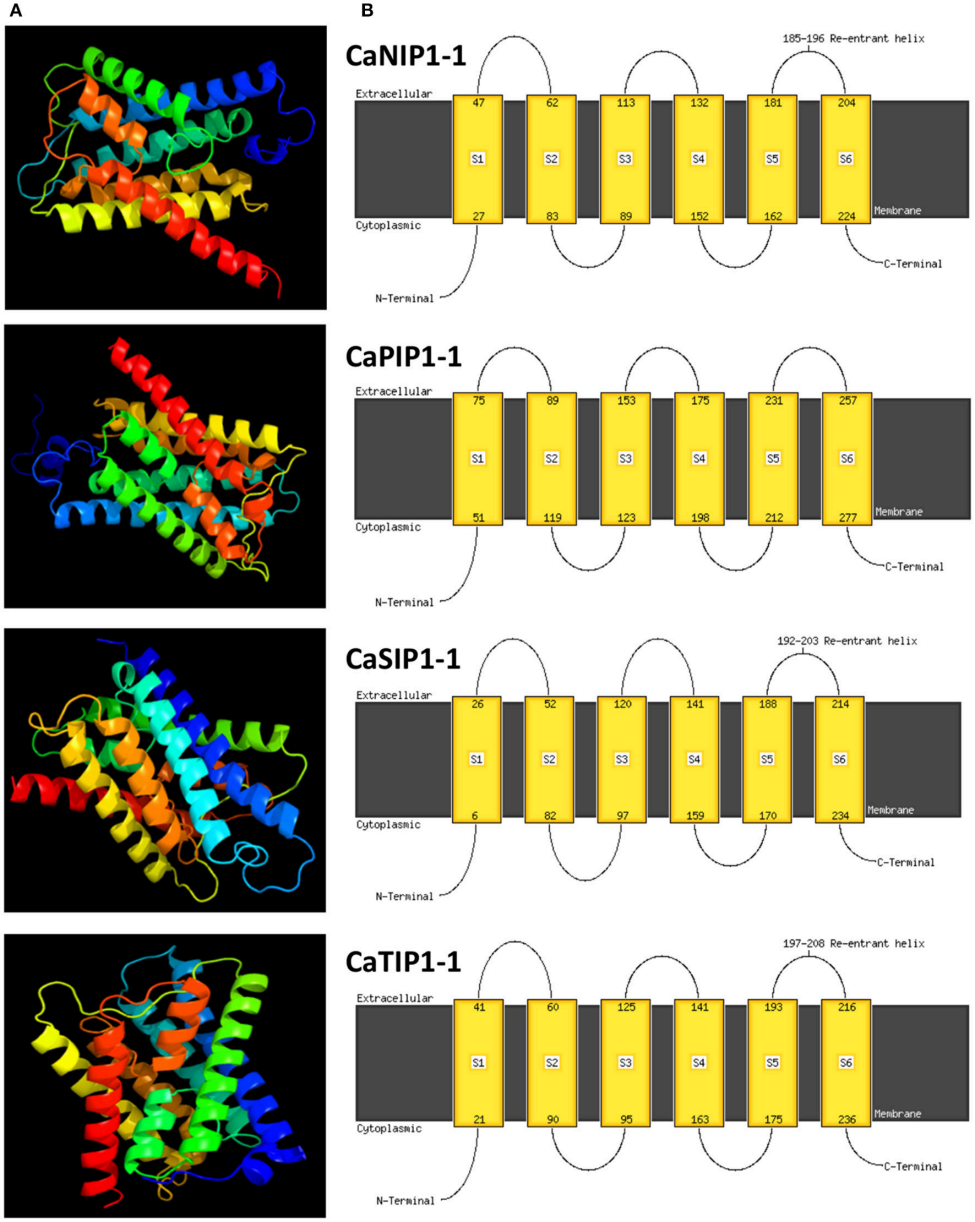
**Predicted 3D structures and transmembrane helix of four selected CaAQP proteins. (A)** 3D structure and **(B)** TM helix of four CaAQPs representing each subfamily namely CaNIP1-1, CaTIP1-1, CaSIP1-1, and CaPIP1-1, modeled at >90% confidence level by using Phyre2 server. The extracellular and cytoplasmic sides of the membrane are labeled and the beginning and end of each transmembrane helix illustrated with a number indicating the residue index.

Molecular dynamics simulations have provided insight into the solute permeation rate and selectivity of AQPs by providing dynamic and energetic information which usually difficult to get experimentally (de Groot and Grubmüller, 2005). In order to understand the structural properties of AQP genes in chickpea, three-dimensional (3D) protein models of all CaAQPs were constructed using phyre2 server and the results are shown in Figure 4 and Supplementary File S5. All the 3D protein models have been constructed with 100% confidence and residue coverage varied from 72 to 98%. Hence, all the predicted 3D protein structures are considered highly reliable and offer a preliminary basis for understanding the molecular function of Aquaporin genes in chickpea. Broadly, the CaAQP 3D protein structure contains the conserved hour-glass model with a pore-forming integral membrane protein containing α-helical bundle forming six TM helices (H1 to H6) and two additional short (half) helices (HE and HB). The loops HE and HB each containing the conserved NPA motifs get close together in the center of the membrane. Homology-based 3D structure models provided important insights about the boron (B) and silicon (Si) substrate selectivity and passage capabilities of barley and soybean AQPs, respectively (Deshmukh et al., 2013; Tombuloglu et al., 2016). This pointed out that the homology-based 3D modeling of plant AQPs can be effectively used for understanding the substrate specificity and molecular function of AQP genes.

### Expression profile of CaAQP genes in different tissue

**Figure 5:**
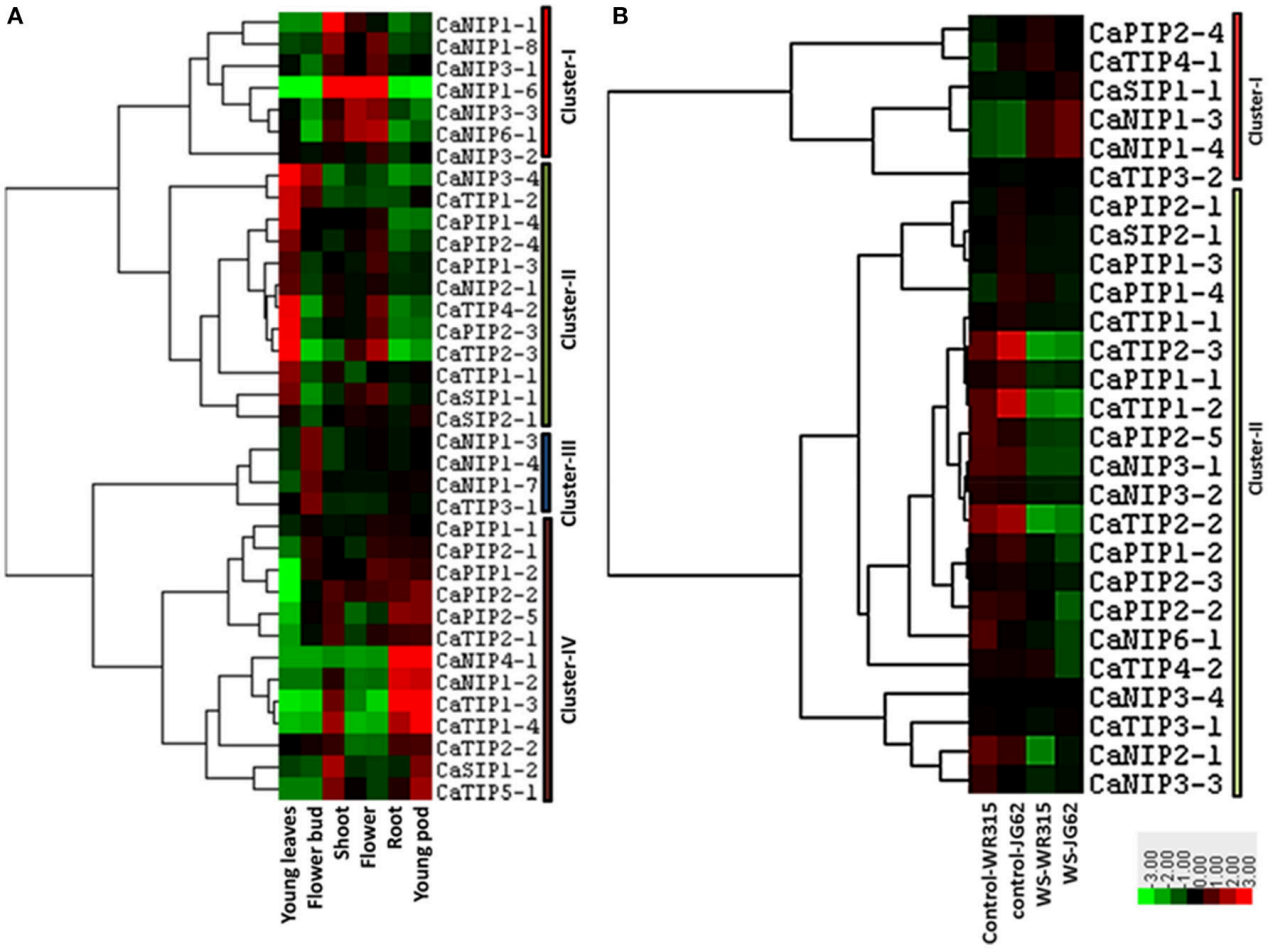
**Expression patterns of CaAQP genes in different tissues and under fusarium wilt stress**. The heatmap with hierarchical clustering was created based on the FPKM value of CaAQPs. The scale represents the relative signal intensity of FPKM values where Red color represents higher expression while green color represents the lower expression. **(A)** Heat map of CaAQPs in young leaves, flower bud, shoot, flower, root, and young pod. **(B)** Heat map of CaAQPs under wilt stress in two different genotypes.

Exclusive or highly confined expressions of some of the AQPs in specific tissues such as root or leaf have been observed in several plants. The cell type localization of aquaporin expression profile can provide clues about their potential physiological roles. In order to gather information on the potential role of the CaAQP genes in chickpea, we analyzed RNA-seq data of young leaves, shoot, shoot apical meristem, flower, flower bud, young pod, and root. We observed the expression of all 40 CaAQPs in at least one of the tissue types. The expression of CaAQP varies in tissues and developmental stages. 28 CaAQP genes showed expression in all the tissue types analyzed in this study (Figure 5A and Supplementary Table 2). From the remaining 12 AQP genes, 10 genes (CaNIP1-1, CaTIP1-3, CaTIP1-4, CaNIP1-5, CaTIP3-2, CaNIP1-2, CaNIP1-8, CaNIP1-9, CaNIP4-1, CaTIP5-1, and CaNIP1-6) were not represented by any sequence reads in either any of the three of the tissue type analyzed, whereas the CaTIP4-1 was only detected in young leaves. Gene clustering and heat map of the CaAQPs showed that the CaPIP1-1, CaPIP2-3, CaPIP1-4, CaPIP2-4, CaPIP2-1, CaPIP1-3 and CaTIP1-1 had high expression in all the eight tissues indicating the constitutive transport process throughout the plant. Gene clustering based on the expression values formed four gene clusters, cluster- I contained seven CaAQP genes that were mainly highly expressed in the shoot, flower, and root, whereas 12 genes in cluster-II genes were mainly highly expressed in the young leaves and roots (Figure 5A). Cluster-I contains only CaNIP genes, whereas the cluster-II contains four TIPs and PIPs and two SIP and NIPs. Cluster-III contains four genes (CaNIP1-3, CaNIP1-4, CaNIP1-7, and CaTIP3-1) and mainly highly expressed in flower bud. The cluster-IV contains 13 genes, mainly and highly expressed in the shoot, shoot apical meristem and young pod (Figure 5A).Highly expressed CaAQPs in reproductive tissue might be associated with regulation of water and nutrient transport in chickpea as many reproductive development processes in plants involves the movement of water between cells or tissues. Recently, two members of NIP subfamily have been reported to involve in the movement of water in reproductive tissue and in pollen development and pollination (Di Giorgio et al., 2016). We also observed higher expression of six CaNIPs (cluster-I) in flower tissue sample indicating the potential involvement of these genes in similar function. Higher expression of some of the chickpea AQPs in tissues with high water fluxes such as fast growing regions in leaf, shoot, and root where actual water uptake occurred have been observed in many other plants. It was hypothesized that the high level of expression in these tissues permit osmotic equilibration between the cytosol and the vacuolar content, and permit rapid transcellular water flow when required (Barrieu et al., 1998). All these tissue/organ level expression data suggested that CaAQPs might involve in regulating water transport, physiology and developmental process. To further detail the understanding of the role of CaAQPs in water transport and other developmental process, knowledge of their expression at the cellular level is essential.

### Expression pattern of CaAQP genes under biotic and abiotic stresses

Expression of several plant AQPs is regulated by abiotic and abiotic stresses, and the altered expression can lead to stresses tolerance in plants (Maurel et al., 2008). To predict the potential role of the CaAQPs in chickpea under diverse environmental stresses, RNA-seq dataset of biotic stress (fusarium wilt) and abiotic stress (drought, cold and salinity stress) were analyzed. In total, transcripts of 26 AQP genes were detected in the fusarium wilt expression datasets (Figure 5B and Supplementary Table 3). Two main clusters were formed, Cluster-I contains seven CaAQP genes with high expression levels in wilt stressed condition as compared to the untreated samples. Cluster-II contains 19 AQP genes with high expression levels in untreated samples as compared to fusarium wilt treated samples. The *Fusarium oxysporum* fungus infects the host plant via the roots or stem and induces wilting of plants due to disruption of water balance. The disturbed water balances resulted in reduced root water uptake, increased resistance to water flow through xylem elements and increased water losses from damaged cells (Wang M. et al., 2012, 2015). This type of physiological alteration in chickpea plants in response to *Fusarium oxysporum* infection likely regulated the expression of some of the observed changes in expression of CaAQP genes. Recently, Tian et al. reported differential expression of 13 Arabidopsis AtPIPs and the involvement of AtPIP1;4 in the transport pathogen-induced apoplastic H2O2 to the cytoplasm of plant cells in response to bacterial pathogen *Pseudomonas syringae* infection (Tian et al., 2016). The cytoplasmic H_2_O_2_ triggered the pathogen-associated molecular pattern (PAMP)-triggered immunity (PTI) pathway, which conferred resistance to the pathogen. With these increasing evidences of the involvement of AQPs in biotic stress, the CaAQPs and expression profiles presented in present work provide a foundation and resource to study the role of AQPs in resistance mechanism of two most important diseases of chickpea fusarium wilt and ascochyta blight.

**Figure 6:**
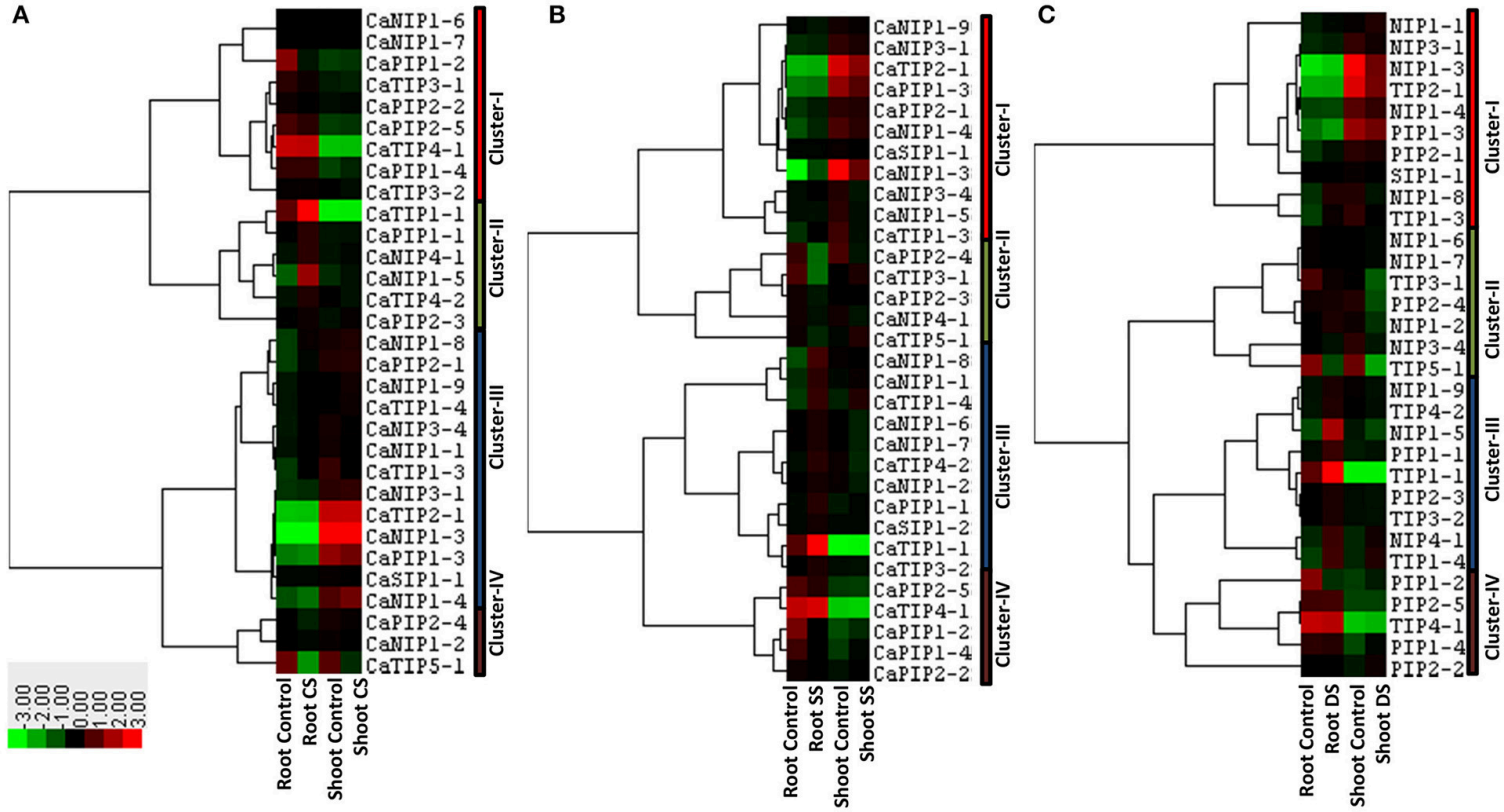
**Expression patterns of CaAQP genes under different abiotic stresses**. Heat maps with hierarchical clustering were created based on the FPKM values of CaAQPs under different abiotic stress treatments and the control sample. **(A)** Heat map of CaAQPs under cold stress (CS), **(B)** Salinity stress (CS), and **(C)** drought stress (DS).

Several pieces of evidence of the AQP involvement in abiotic stress that disturbs plant osmotic balance such as cold, salinity and drought have been reported (Luu and Maurel, 2005). In order to understand the role of CaAQPs in abiotic stress conditions, we analyzed the response of AQPs in root and shoot tissue under cold, salinity and drought stress conditions. Transcripts of 31 AQP genes were detected in shoot and roots of cold stressed (4°C) chickpea seedlings (Figure 6A and Supplementary Table 4). These genes were clustered into four main clustered. Cluster-I contains nine AQPs with high-expression levels in root tissue under both control and cold stress condition as compared to the shoot. Whereas, Cluster-III contains 13 genes with the opposite expression profile of cluster I, i.e., low expression levels in root tissue, but high-expression in the shoot tissue. On average in both of these clusters, AQPs showed constitutive expression profile in control and stress conditions. The cluster-II contain six genes (CaTIP1-1, CaTIP4-2, CaPIP1-1, CaPIP2-3, CaNIP1-5, and CaNIP4-1) and were highly expressed, whereas cluster-IV contained three genes (CaPIP2-4, CaTIP5-1, and CaNIP1-2) under-expressed in root tissue under cold stress (Figure 6A).

Transcripts of 32 CaAQPs were detected in shoot or root or both tissues under salinity stress condition (Figure 6B and Supplementary Table 4). These genes were clustered into four main clusters. Cluster-I contains 11 genes, highly expressed in shoot tissue, whereas cluster-V contains five CaAQPs, highly expressed in root tissue. Cluster-II contains five genes (CaTIP3-1, CaTIP5-1, CaPIP2-3, CaPIP2-4, and CaNIP4-1) down-regulated under salinity stress, whereas Cluster-III contains three genes (CaTIP1-4, CaNIP1-8, and CaNIP1-4) up-regulated under salinity stress in both root and shoot.

We also analyzed the expression profiles of AQP genes in response to drought stress in root and shoot tissue. Results showed that transcripts of 31 CaAQPs were detected in at least one condition i.e., control/stress of root and shoot (Figure 6C and Supplementary Table 4). Based on their expression values, the CaAQPs were clustered into four main clusters. Cluster-I contains ten genes highly expressed in root tissue as compared to shoot tissue. Cluster-IV contains five CaAQPs and have opposite expression profile as in cluster-I i.e., genes showed low expression in roots as compared to shoot. However, AQPs in cluster I and IV shows constitutive expression pattern under control and stress condition. Cluster III contains seven CaAQPs that were highly expressed in root control and drought stress condition, however under-expressed (down-regulated) in shoot tissue under drought stress conditions. This cluster contains CaTIP3-1, CaTIP5-1, CaPIP2-4, CaNIP1-2, CaNIP1-6, CaNIP1-7, and CaNIP3-4 genes. Cluster-III contains nine CaAQPs low-expressed in the shoot, but high-expressed in roots under drought condition. This cluster mainly contains CaTIPs (CaTIP1-1, CaTIP1-4, CaTIP2-3, and CaTIP3-2), CaPIP1-1, CaPIP2-3, CaNIP1-5, CaNIP1-9, and CaNIP4-1 genes.

Overall expression profiles of CaAQPs in response to all stress indicated that some of the CaAQPs such as CaTIP1-1 and CaPIP1-1 showed up-regulation in cold, drought and salinity stress. Whereas, some of the CaAQP genes also have an organ-specific response such as CaTIP1-1 and CaPIP2-5 that were highly expressed in root tissue and CaTIP2-1 and CaNIP1-3 that were highly expressed in shoot tissue. Differential expression of 13 unigenes encoding PIPs, TIPs, and NIPs in root tissue under terminal drought stress has been reported (Deokar et al., 2011). The majority of these genes were down-regulation under terminal drought conditions. Similar pattern of aquaporin gene expression under stress condition was also observed in Arabidopsis (Jang et al., 2004; Alexandersson et al., 2005, 2010; Boursiac et al., 2005) and other plant species (Smart et al., 2001; Mahdieh et al., 2008; Secchi and Zwieniecki, 2014), although up-regulation and constitutive expression pattern of some of the aquaporins under stress conditions were also observed. The increase and decrease in expression of AQP under water stress have been associated with a general pattern of AQP regulation under different stress conditions. The decrease in the expression of AQPs under stress can effectively prevent water losses under stress condition, whereas the increase in AQP expression helps plants to direct water flow to specific organ that is critical for plant survival under stress condition or necessary for its rapid recovery upon rehydration (Alexandersson et al., 2005, 2010). Therefore, the observed expression pattern of many of CaAQPs in response to biotic and abiotic stresses suggested the potential role of CaAQPs in biotic and abiotic stress response in chickpea.

Our expression analysis categorized the CaAQPs into different groups based on their expression profiles. This information will be helpful for functional characterization of CaAQP genes in chickpea.

### Promoter profiling of CaAQP

The *cis-*acting regulatory elements (CREs) are DNA region in the promoter, where a number of transcription factors can bind and regulate the transcription of nearby genes. Profiling of the promoter region for the CREs can provide information on gene regulatory networks. To predict the CREs, the promoter sequences (1000 bp upstream DNA sequences) of the predicted transcription initiation site of 40 CaAQP gene were extracted and subjected to promoter profiling using PlantCARE web tool (Lescot et al., 2002). The most common *cis-*acting elements present in CaAQPs were known to involve in light response such as Box-4, G-Box, I-Box, ACE etc., (Supplementary Table 5). Among the light responsive elements, the Box-4 is the most abundant and present in almost all CaAQPs, except CaPIP1-3, CaTIP1-2, and CaNIP4-1. The ARE, HSE and I-Box light regulating *cis-*acting element were present in the majority of the members of chickpea aquaporin subfamily, but absent in the members of CaSIP subfamily. Light is one of the most important environmental factors that regulate plant growth and development. Besides the source of energy, light regulates gene expression network at many levels (Jiao et al., 2007; Petrillo et al., 2014). Effect of light (quantity and quality) on gene expression and function of individual or subfamily of AQPs has been reported in many plants (Baaziz et al., 2012). The presence of several light responsive *cis-*active elements in specific chickpea AQPs suggested that some of the chickpea AQPs might be regulated by light-dependent activations.

Plant hormone responsive *cis-*elements such as AuxRR-core, ABRE, ERE, TCA-element, and GARE-motif were also abundantly found in several chickpea AQPs, suggested that the expression of some of the chickpea AQPs might be regulated by the different type of hormones. Several *cis-*elements responsive to biotic and abiotic stresses such as W-box, TC-rich repeat, HSE, and TCA-element were also found in CaAQPs. The presence of these stress-responsive *cis-*elements in the promoter region of CaAQPs indicated their potential involvement in regulating stress responses in chickpea. Two AQPs genes (CaTIP1-4 and CANIP4-1), showed higher expression in both roots and shoot tissues under drought and salinity stresses and root tissue under the cold condition contains stress-responsive *cis-*acting elements ABRE, LTR, and MBS. The presence of these stress-responsive *cis-*elements in CaAQPs correlates to high expression in response to drought, salinity, and cold stress. The ABRE and MBS *cis-*elements have been previously reported to be involved in ABA-mediated osmotic stress signaling in the regulation of drought-inducible gene expressions (Abreu and Aragão, 2007; Hou et al., 2016), whereas *cis-*element LTR is a low-temperature-responsive element and involved in the expression of cold-regulated genes (Brown et al., 2001). The link between the presence of stress-related *cis-*acting elements and the differential expression of aquaporin genes under various stress conditions have been reported in several plants (Alexandersson et al., 2010; Wang L. et al., 2015).

The circadian regulation of aquaporin gene expression and the correlation with the modulation water dynamics have been well demonstrated in several plants (Lopez et al., 2003; Hachez et al., 2008, 2012; Sakurai-Ishikawa et al., 2011; Takase et al., 2011). Promoter analysis these AQP genes also showed the presence of abundant circadian regulatory elements (Liu et al., 2009; Lopez et al., 2013). Similarly, in our analysis, we also observed high representation of circadian (CAANNNNATC) *cis-*acting regulatory element involved in circadian control in the CaAQPs promoter region. The presence of a circadian-related *cis-*acting regulatory element suggested that some of the CaAQPs might be involved in the diurnal transport of water through roots as observed in Arabidopsis and maize plants (Lopez et al., 2003; Takase et al., 2011).

Comparative analysis of promoter sequences of Arabidopsis, rice, maize, grape, poplar, Medicago, and Glycine max also observed the high abundance of the conserved light, hormone, biotic and abiotic stress-related response regulatory elements (Lopez et al., 2013). Previous studies also demonstrated that photosynthesis or protein biosynthesis and defense signaling are largely conserved across the species at the promoter element level (Rushton et al., 2002; Vandepoele et al., 2006; Ravel et al., 2014). Promoter profiling and the conserved regulatory elements identified in chickpea AQPs promoter might have conserved regulatory modules as observed for AQP genes in other model plants.

## Conclusions

This study presents the results of the identification and genome-wide survey of the aquaporin genes in chickpea. A total of 40 AQP genes were identified and characterized for their genomic organization, protein sequence properties, gene duplications, phylogenetic relationships with Arabidopsis and closely related legumes, homology-based 3D models and promoter sequence profiles for conserved *cis-*acting elements. The expression profiles of CaAQPs in different plant parts and under biotic and abiotic stress conditions provided valuable data to understand the involvement of AQPs under different osmotic stress conditions in chickpea.

## Author contributions

AD: Conducted the experiments, analyzed and summarized the data. AD and BT: Wrote and finalized the manuscript; BT: Conceived and directed the project.

## Funding

The authors were supported by funding from the Saskatchewan Pulse Growers and the Saskatchewan Ministry of Agriculture.

### Conflict of interest statement

The authors declare that the research was conducted in the absence of any commercial or financial relationships that could be construed as a potential conflict of interest.

## Supplementary material

Supplementary File S1 **Nucleotide and amino acid sequences of 40 CaAQP gene identified in this study**.

Supplementary File S2 **Comparison of number of different AQP subfamily members in chickpea, Arabidopsis, Medicago, common bean, and soybean**.

Supplementary File S3 **Multiple sequence alignment of deduced amino acid sequences of 40 CaAQP genes**. Sequence alignment was performed using CLUSATLW. Transmembrane helices (TM1–TM6) and two NPAs (LB and LE) are highlighted in gray color, P1–P5 residues highlighted in blue color, and ar/R selectivity filter residues highlighted in green color. The NPA motifs are shown in red color.

Supplementary File S4 **Summary of specificity-determining positions (SDP) identified in the CaAQPs**. SDP positions identified in the present study and not found conserved with the reported SDPs in AQPs are highlighted in red color.

Supplementary File S5 **Predicted 3D structure of the 40 CaAQP protein generated using Phyre 2 server**.

Supplementary Table 1 **Protein sequence similarity matrix between members of CaAQP genes. Protein sequence similarity expressed in terms of percentage identity calculated using sequence Identity Matrix option available in Bioedit software**.

Supplementary Table 2 **FPKM values for all the detected CaAQP genes in the shoot, young leaves, shoot apical meristem, flower, Flower bud, young pod, and root tissue of chickpea plant**.

Supplementary Table 3 **FPKM values for all the detected CaAQP genes in fusarium wilt expression datasets**.

Supplementary Table 4 **FPKM values for all the detected CaAQP genes in drought, cold and salinity expression datasets**.

Supplementary Table 5 **Analysis of cis-acting elements in the 1000bp sequence upstream of the translation initiation codon of CaAQP genes**.
