# Supplementary material for: Genome-Wide Analysis of the Aquaporin Gene Family in Chickpea (Cicer arietinum L.)
Source: Front Plant Sci. 2016 Nov 29;7:1802. doi: 10.3389/fpls.2016.01802 (PMC5126082; doi:10.3389/fpls.2016.01802)
Supplement: Supplementary File S1 — Nucleotide and amino acid sequences of 40 CaAQP gene identified in this study. [file DataSheet1.PDF]

>Ca_06493 (CaNIP1-1)
ATGGAAGAAGGTGGATTTAAGGAACTAACTGGAAACGAGGGGGGGTTTTGCGGTTCTCCAGAGG
TAGTTCAAGTGATACAAAAGGTAATAGCAGAGGTGATAGGATCATACTTCTTAATATTTGCAGG
GTGTTGCTCAGTGGTTTTGAATAAAGTGGAAGGTAGTAAAGGGACAATCACGTTTCCTGGAATT
TGTATTGTATGGGGTGTGTCAGTCATGATCTTGGTTTATGCTCTTGGTCATATCTCTGGTGCAC
ATTTCAATCCTGCTGTTCCATTGTATCTTATTGCTCAGGTGCTAGGATCTATCCTTGCTAGTGG
GACATTATATCTTCTATTCGATGATCTAAATGAAAGTACTTATTTTGGAACAGTACCAGCGGGT
TCTGATGTTCAATCTCTTGTCTTTGAGATAATCACATCATTTCTCTTGATGTTTGTTATCTCTG
CAGTTTCCACAGACAATAGAGCGATTGGAGAACTGGCAGGGATTGCTGTTGGTATGACGATTAT
GATAGACGTCTTCATTGCTGGGCCCATCTCAGGTGCATCTATGAACCCAGCGAGGAGCTTTGGA
CCGGCTTTGGTGATGCACATTTACGATGGATTTTGGATTTATATAGTTGGACCATTTGTTGGTG
CCATACTAGGTGCCTCGGCATACAACCTCATTAGATTCACTGACAAACCACTTAAGGAAATAAG
CGGTAGCTCAAAGTTCCTCAAAAGCGTTTCTAGAGCAACAAGTTTTCGGTAG
>Ca_06493 (CaNIP1-1)
MEEGGFKELTGNEGGFCGSPEVVQVIQKVIAEVIGSYFLIFAGCCSVVLNKVEGSKGTITFPGI
CIVWGVSVMILVYALGHISGAHFNPAVPLYLIAQVLGSILASGTLYLLFDDLNESTYFGTVPAG
SDVQSLVFEIITSFLLMFVISAVSTDNRAIGELAGIAVGMTIMIDVFIAGPISGASMNPARSFG
PALVMHIYDGFWIYIVGPFVGAILGASAYNLIRFTDKPLKEISGSSKFLKSVSRATSFR

>Ca_16129 (CaNIP1-2)
ATGGCCAACAAACGTGAAGGCAATATTCAATTAGACGAGGAAACATCATCAAGTGTGGATGAAG
GCAACCCTTCCATCATGCAATTTTGTTGTTCATCAAACCATACTATAACCTTGATACAAAAGGT
GATTGCAGAAATAATTGGGACATATTTTTTGGTGTTTGCTGGGTGTGGTGCTGTAGTAGTGAAT
AAAATATATGGCTCAGTCACATTTCCAGGGATTTGTATCACTTGGGGACTAATTGTAATGGTTA
TGTGTTACTCTCTTGGTCATATCTCTGGAGGCCACTTCAACCCTGCAGTTACCATCACTTGGAC
CCTCTTTCGTAGGATCTCAATTAAAGAGGCTCCACTATACATTTTTGCTCAGTTGCTGGGGTCC
ACACTTGCAAGTGGGACATTATCTCTAATGTTTGATATCACACCAAAAACTTATTTTGGAACGG
TACCAAGTGGATCAAATGGGCAATCTTTAGTTGTTGAAATCATCATCAGTTTTCTTCTAATGTT
TGTCGTTTCAGCAGTTTCCACTGATGATAGAGCGGTGAATGACATGGGAGGTGTTGCAGTTGGA
ATGACTATAATGTTGAATCTTTTTATTGCTGGGCCTGTATCAGGGGCTTCAATGAATCCAGCAA
GAAGTATTGGGCCAGCACTTGTGAAACATATTTACAAAGGATTATGGATATACATTGTTGGTCC
AATTGTTGGAGCCATAGCTGGAGCAATTGCTTATAACTTTCTTAGATCCATAGAAAAGTCACCT
ACAGAGTCACCATTGAGGAACATCATTGTAGTTTGA
>Ca_16129 (CaNIP1-2)
MANKREGNIQLDEETSSSVDEGNPSIMQFCCSSNHTITLIQKVIAEIIGTYFLVFAGCGAVVVN
KIYGSVTFPGICITWGLIVMVMCYSLGHISGGHFNPAVTITWTLFRRISIKEAPLYIFAQLLGS
TLASGTLSLMFDITPKTYFGTVPSGSNGQSLVVEIIISFLLMFVVSAVSTDDRAVNDMGGVAVG
MTIMLNLFIAGPVSGASMNPARSIGPALVKHIYKGLWIYIVGPIVGAIAGAIAYNFLRSIEKSP
TESPLRNIIVV

>Ca_08631 (CaNIP1-3)
ATGGATGACAATTCAGCTAGCAATGGAACAATCAATGAGGTCGTTTTAAACGTAAACAAGGATG
ACTTAAAGATAACTGAAAACTCAACCGCCCCTGCCACTGCTTCTTTCTTGCAAAAGTTGGTAGC
TGAAGTGGTAGGCACCTATTTCTTGATATTTGCTGGTTGTGCTTCAGTGGTGGTGAACAAGAAC
AATGAAAATGTTGTAACACTTCCTGGGATTTCAATTGTTTGGGGACTTGCTGTCATGGTCTTGG

TTTACTCTCTTGGTCATATCTCTGGTGCTCATTTCAATCCTGCTGTTACAATTGCTTTTGCCAC
CACCAGAAGATTTCCCCTCAAACAGGTACCAGGCTATGTAGTAGCTCAAGTCCTTGGATCGACA
CTTGCAAGTGGAACTCTTAGATTACTATTTAGTGGGAAGGATAACCAATTTGCAGGAACACTCC
CAGATGGATCTAACTTGCAAGCTTTTGTTGTTGAATTCATAATCACTTTTTATCTTATGTTTAT
CATATCTGGAGTTGCCACCGATAATAGAGCGATTGGTGAATTGGCTGGAATTGCCGTTGGGTCT
ACTGTACTTTTGAATGTGATGTTTGCAGGGCCAATAACAGGGGCATCAATGAATCCAGCAAGAA
GCATAGGACCTGCATTTGTACACATGGAATATAATGGAATATGGATATATTTGGTGTCTCCAAT
TCTAGGGGCTGTGGCTGGTGCATGGGTTTATAACATCATTCGATATACGGATAAGTCGGTTCGT
GAGATCACCAAAAGTGCATCTTTCCTCAAAGGAGTAAAGCCATAG
>Ca_08631 (CaNIP1-3)
MDDNSASNGTINEVVLNVNKDDLKITENSTAPATASFLQKLVAEVVGTYFLIFAGCASVVVNKN
NENVVTLPGISIVWGLAVMVLVYSLGHISGAHFNPAVTIAFATTRRFPLKQVPGYVVAQVLGST
LASGTLRLLFSGKDNQFAGTLPDGSNLQAFVVEFIITFYLMFIISGVATDNRAIGELAGIAVGS
TVLLNVMFAGPITGASMNPARSIGPAFVHMEYNGIWIYLVSPILGAVAGAWVYNIIRYTDKSVR
EITKSASFLKGVKP

>Ca_08632 (CaNIP1-4)
ATGGATGACAATTCAGCTAGCAATGGAACAATCAATGAGGTCGTTTTAAACGTAAACAAGGATG
ACTTAAAGATAACTGAAAACTCAACCGCCCCTGCCACTGCTTCTTTCTTGCAAAAGTTGGTAGC
TGAAGTGGTAGGCACCTATTTCTTGATATTTGCTGGTTGTGCTTCAGTGGTGGTGAACAAGAAC
AATGAAAATGTTGTAACACTTCCTGGGATTTCAATTGTTTGGGGACTTGCTGTCATGGTCTTGG
TTTACTCTCTTGGTCATATCTCTGGTGCTCATTTCAATCCTGCTGTTACAATTGCTTTTGCCAC
CACCAGAAGATTTCCCCTCAAACAGGTACCAGGCTATGTAGTAGCTCAAGTCCTTGGATCGACA
CTTGCAAGTGGAACTCTTAGATTACTATTTAGTGGGAAGGATAACCAATTTGCAGGAACACTCC
CAGATGGATCTAACTTGCAAGCTTTTGTTGTTGAATTCATAATCACTTTTTATCTTATGTTTAT
CATATCTGGAGTTGCCACCGATAATAGAGCGATTGGTGAATTGGCTGGAATTGCCGTTGGGTCT
ACTGTACTTTTGAATGTGATGTTTGCAGGGCCAATAACAGGGGCATCAATGAATCCAGCAAGAA
GCATAGGACCTGCATTTGTACACATGGAATATAATGGAATATGGATATATTTGGTGTCTCCAAT
TCTAGGGGCTGTGGCTGGTGCATGGGTTTATAACATCATTCGATATACGGATAAGTCGGTTCGT
GAGATCACCAAAAGTGCATCTTTCCTCAAAGGAGTAAAGCCATAG
>Ca_08632 (CaNIP1-4)
MDDNSASNGTINEVVLNVNKDDLKITENSTAPATASFLQKLVAEVVGTYFLIFAGCASVVVNKN
NENVVTLPGISIVWGLAVMVLVYSLGHISGAHFNPAVTIAFATTRRFPLKQVPGYVVAQVLGST
LASGTLRLLFSGKDNQFAGTLPDGSNLQAFVVEFIITFYLMFIISGVATDNRAIGELAGIAVGS
TVLLNVMFAGPITGASMNPARSIGPAFVHMEYNGIWIYLVSPILGAVAGAWVYNIIRYTDKSVR
EITKSASFLKGVKP

>Ca_08630 (CaNIP1-5)
ATGGCTAATAATAATTCAGCAAGAATTGAAACTCTTGATGTTGTTTTAGATGTAAACAAGGACT
CCTCCAGAACATGTGAAGGCTCTGACTCCTATGTCTCTGTCCCTTTCTTGCAGAAGTTAATAGC
AGAGATGGTGGGAACATATTTCTTGATATTTGCAGGATGTGCTTCTGTAGTGGTGAACAATAAC
AAAGACAACGTTGTAACACTTCCTGGGATTGCAATTGTTTGGGGACTCACTCTTATGGTACTTG
TTTACTCTCTTGGTCATATCTCTGGTGCTCATTTCAATCCTGCTGTCACCCTTGCTTTCGCTTC
AACAAGGAGATTTCCTTTAGTACAGGTACCAGCTTATTTATCAGCTCAGGTCTTGGGAGCTACA
CTTGGTAGTGGAACTTTGAAACTAATATTCAGTGGCAGCCATGATCAGTTTACAGGAACACTTC

CAACTGGATCTAATTTTCAAGCTTTTGTACTTGAATTTATAATCACTTTTTTCCTTATGTTTGT
CATTTCTGGTGTGGCCACCGATAACAGAGCGATTGGTGAATTAGCTGGAATTGCAATTGGATCT
ACATTATTACTTAATGCGATAATTGCAGCACCAATAACAGGAGCATCAATGAATCCAGCTAGAA
GTTTAGGACCTGCATTTGTACACAATAAATACAGAGGAATATGGATATATTTGGTGTCACCGAT
TTTAGGAGCGGTGGCCGGAGCATGGGTGTACAACACTGTGAGGTACACTAACAAGCCATTGCGT
GAGATCACGAAGAGTGCATCCTTCCTGAGAGAAGCAGGGCGTGGTGGACAACAAATAATCTAA
>Ca_08630 (CaNIP1-5)
MANNNSARIETLDVVLDVNKDSSRTCEGSDSYVSVPFLQKLIAEMVGTYFLIFAGCASVVVNNN
KDNVVTLPGIAIVWGLTLMVLVYSLGHISGAHFNPAVTLAFASTRRFPLVQVPAYLSAQVLGAT
LGSGTLKLIFSGSHDQFTGTLPTGSNFQAFVLEFIITFFLMFVISGVATDNRAIGELAGIAIGS
TLLLNAIIAAPITGASMNPARSLGPAFVHNKYRGIWIYLVSPILGAVAGAWVYNTVRYTNKPLR
EITKSASFLREAGRGGQQII

>Ca_00434 (CaNIP1-6)
ATGGGTGATATTTTAGATAGCAATGATGTGGTTTTGAAGGTAGATGATTCAATCATTGAAGATA
GTGTTCCTCTTTTGCAGAAGTTGGTAGCAGAGGTGGTGGGAACATTCATGTTGATATTTGTTGG
TTGTGGTGTAGTGGTGACGAACCTTAACAATGATAATGTAGTGACACTTCCTGGTGTTGCAATT
GTTTGGGGACTTGCTGTTATGGTATTGGCCTATTCTCTCGGTCACGTCTCTGGTGCTCATTTCA
ATCCTGCTGTCACCATTGCTCAGGCTTCCACCAAAAGATTTCCTGTCCAACAGATACCAACTTA
TATAATTGCTCAGCTCTTTGGATCAATACTTGCAAGTGTTGTTCTTAAAGTTATATTCAGTGAC
AAGGAAAATCGTTTTGTAGGAACACTTCCAGCTGGTTCTAACCTCCAAGCTTTTGTGGTCGAAT
TTCTAATCACTTTCTTACTTATGTTCGTTATTTCTGGAGTTGGCACTGATAACAGAGCGGTTAA
TGAATTGGCTGCACTTGCAGTTGGATCTACAGTGCTACTAGTTGTGTTGTTTGCTGGGCCAATC
ACAGGAGCATCAATGAATCCAGCAAGAAGCTTAGGGCCAGCTATTGTGCACCATGAATATAGAG
GAATATGGATATATTTGGTGTCACCTATTCTAGGAGCTTTGGCCGGTACATGGACATATACTTT
CATTAGATACACAAACAAACCAGCAACAATCCCCAAGAGTGCTTCATTCCTCAAAGGAGCTTAA
>Ca_00434 (CaNIP1-6)
MGDILDSNDVVLKVDDSIIEDSVPLLQKLVAEVVGTFMLIFVGCGVVVTNLNNDNVVTLPGVAI
VWGLAVMVLAYSLGHVSGAHFNPAVTIAQASTKRFPVQQIPTYIIAQLFGSILASVVLKVIFSD
KENRFVGTLPAGSNLQAFVVEFLITFLLMFVISGVGTDNRAVNELAALAVGSTVLLVVLFAGPI
TGASMNPARSLGPAIVHHEYRGIWIYLVSPILGALAGTWTYTFIRYTNKPATIPKSASFLKGA

>Ca_00435 (CaNIP1-7)
ATGGCTGATCATTCAGAAAGCAATGGAAACCGTGAGATGGTTTTGAATGTAAATGGTGATGCCT
CTAATATTTGTGACAACTCAAGCATCGAGGAACGTGTGCCCCTTTTGAAGAAGTTGGTAGCAGA
GGTGGTGGGTACATACTTGTTGATATTTGCAGGTTGTGGTGCAGTAGTGGTGAACCTTGACAAA
GACAAAGTAATAACACATCCGGGAATTTCAATTGTTTGGGGACTCACTGTTATGGTATTGGTTT
ACTCTATTGGTCACATCTCTGGTGCTCATTTCAATCCTGCTGTCACTATTGCTCATGCTTCTAC
CAAAAGATTTCCTCTTAAGCAGGTTCCAGCTTATGTAATAGCTCAAGTACTTGGATCTACACTT
GCAAGTGGAACACTTAGACTTATATTCAATGGCAAAGAAAACCATTTTTCAGGAACACTACCCG
CTGGGTCTAATCTTCAATGTTTTGTTGTCGAATTTATAATAACTTTTTATCTCATGTTCGTCAT
TTCTGGAGTTGCCACCGATAATAGAGCGATTGGCGAGTTGGCTGGACTTGCAGTTGGGTCTACA
GTACTGCTAAATGTGATGTTTGCCGGGCCAATCACTGGAGCATCAATGAATCCTGCAAGGAGTT
TAGGACCTGCCATTGTTCATAATGAATACAGAGGAATATGGATATACATAGTGTCAACTACTCT

TGGAGCTATGGCCGGTACATGGGTCTATAATATCATTAGATATACAAACAAACCGGTGCGTGAA
ATTAGCAAGAGTGCCTCTTTCCTTAGAGGAGTACAAAATGGCATAGCTAAGTGA
>Ca_00435 (CaNIP1-7)
MADHSESNGNREMVLNVNGDASNICDNSSIEERVPLLKKLVAEVVGTYLLIFAGCGAVVVNLDK
DKVITHPGISIVWGLTVMVLVYSIGHISGAHFNPAVTIAHASTKRFPLKQVPAYVIAQVLGSTL
ASGTLRLIFNGKENHFSGTLPAGSNLQCFVVEFIITFYLMFVISGVATDNRAIGELAGLAVGST
VLLNVMFAGPITGASMNPARSLGPAIVHNEYRGIWIYIVSTTLGAMAGTWVYNIIRYTNKPVRE
ISKSASFLRGVQNGIAK

>Ca_00436 (CaNIP1-8)
ATGGGTGATATTTCAGCTACCAATGATGTGGTTTTAAATGTTGATGCCAATGACTCAATCATTG
AAGATAGTGTTCCTCTTTTGCAGAAGTTGGTAGCAGAGGTGGTGGGAACATTCATGTTGATATT
TGTTGGTTGTGGTGTAGTGGTGACGAACCTTAACAATGATAATGTAGTGACACTTCCTGGTGTT
GCAATTGTTTGGGGACTTGCTGTTATGGTATTGGCCTATTCTCTCGGTCACGTCTCTGGTGCTC
ATTTCAATCCTGCTGTCACCATTGCTCAGGCTTCCACCAAAAGATTTCCTGTCCAACAGATACC
AACTTATATAATTGCTCAGCTCTTTGGATCAATACTTGCAAGTGTTGTTCTTAAAGTTATATTC
AGTGACAAGGAAAATCGTTTTGTAGGAACACTTCCAGCTGGTTCTAACCTCCAAGCTTTTGTGG
TCGAATTTCTAATCACTTTCTTACTTATGTTCGTTATTTCTGGAGTTGGCACTGATAACAGAGC
GGTTAATGAATTGGCTGCACTTGCAGTTGGATCTACAGTGCTACTAGTTGTGTTGTTTGCTGGG
CCAATCACAGGAGCATCAATGAATCCAGCAAGAAGCTTAGGGCCAGCTATTGTGCACCATGAAT
ATAGAGGAATATGGATATATTTGGTGTCACCTATTCTAGGAGCTTTGGCCGGTACATTGACATA
TACTTTCATTAGATACACAAAGTGA
>Ca_00436 (CaNIP1-8)
MGDISATNDVVLNVDANDSIIEDSVPLLQKLVAEVVGTFMLIFVGCGVVVTNLNNDNVVTLPGV
AIVWGLAVMVLAYSLGHVSGAHFNPAVTIAQASTKRFPVQQIPTYIIAQLFGSILASVVLKVIF
SDKENRFVGTLPAGSNLQAFVVEFLITFLLMFVISGVGTDNRAVNELAALAVGSTVLLVVLFAG
PITGASMNPARSLGPAIVHHEYRGIWIYLVSPILGALAGTLTYTFIRYTK

>Ca_00437 (CaNIP1-9)
ATGGATGATAATTCAGAAAGCAATGAAATTCATCATGAGGTGGTTTTAAATGTAAACGGTGATG
CATCCAAAAATTGTGATGAGTCAGGTTTCAAAGACTCTGTGCCCCTTTTGAAGAAGTTGGTAGC
AGAAGTGGTGGGAACATACTTGATGATATTTGCCGGATGTGCTGCTGTTTTGGTTAATCTTAAC
AATGATCATGTAGTGACACTTCCTGGAATCGCAGTTACTTGGGGATTCACTGTTATGGTATTAA
TTTATTCTCTTGGTCACATCTCTGGTGCTCATTTCAACCCTGCTGTTACCATTGCTCATGCTTC
CACTAAAACATTTCCCTTAAAGCAGGTACCGGCATATATTATAGCTCAGGTCCTTGGATCCATA
CTAGCAAGTGGAACGCTCAAACTTATATTTAATGGCAAAGATGGTCATTTTATCGGAACACTCC
CAACTGGTTCTAACCTCCAAGCTTTTGTAATCGAGTTCATATGCACTTTCTTCCTTATGTTTGT
TATTACTGCAGTAGCCACCGATGACAGAGCGATTGGTGAGTTGACCGGGATTGCAGTTGGGTGT
ACAATATTGATAGATATATTGTTTGCAGGACCAATCACGGGAGCTTCGATGAATCCGGCAAGGA
GCTTAGGACCTGCTGTTGTGGATCATGAGTATAGAGGACTATGGATATATTTGATTTCACCCAT
TCTGGGAGCTTTGATAGGCACATGGACTTACAATTTCATTAGGCACAAGAACAAACCAATGTGT
GATGAGCTCACCAAGATTGTCCCTACCAAGATTGTCCCTTTCTTCAGGAGCAGCAGGATGTAA
>Ca_00437 (CaNIP1-9)
MDDNSESNEIHHEVVLNVNGDASKNCDESGFKDSVPLLKKLVAEVVGTYLMIFAGCAAVLVNLN
NDHVVTLPGIAVTWGFTVMVLIYSLGHISGAHFNPAVTIAHASTKTFPLKQVPAYIIAQVLGSI

LASGTLKLIFNGKDGHFIGTLPTGSNLQAFVIEFICTFFLMFVITAVATDDRAIGELTGIAVGC
TILIDILFAGPITGASMNPARSLGPAVVDHEYRGLWIYLISPILGALIGTWTYNFIRHKNKPMC
DELTKIVPTKIVPFFRSSRM

>Ca_21333 (CaNIP2-1)
ATGGACAGAAGAACACACAGTTTGGTCAATGCTACAAATGACTTTCAAAACCACATAACACAAA
AGCAATCACTGTATCCTTCTGGTTTTCCAAGAAAGGTACTTGCAGAGGTTATAGGGACATATTT
GTTGGTGTTTGTTGGGAGTGGGAGTGCAGCTATGAATGCAATTGATGAAAACAAAGTATCAAAA
TTGGGAGCATCAATGGCAGGTGGATTCATAGTTACAGTTATGATTTATGCAATTGGACATATCT
CTGGTGCACACATGAATCCTGCTGTTTCCTTAGCTTTTGCCACCGTTAGCCATTTTCCTTGGAA
ACAGGTACCATTTTACATTGCAGCTCAACTCACAGGAGCAATTTCAGCTTCATATACATTGAAA
GTTTTGCTAGAGCCATCAAAGCAACTTGGTGCAACATCACCTTCTGGATCTAACATTCAAGCAT
TAATCATAGAAATTGTGACCACCTTCACCATGGTGCTCATCTCCACCGCCGTCTCCACCGACCC
AAAAGCTATAGGAGAGCTTTCAGGAGTTGCAGTTGGTTCATCTGTTTGCATAGCAAGCATTGTG
GCTGGACCAATATCGGGGGGGTCAATGAACCCAGCAAGGACATTAGGTCCAGCAATTGCTACTT
CATCCTACAAAGGAATTTGGGTTTATATGGTTGGACCAATTACTGGTGCACTTTTAGGTACATG
GTCTTATGTTGTGATTCAGGAGACAAATAAACAAGCTCTTACAACTTCACTCAAGCTACACCAT
GAGATGAAAGGGATTGAGTTAGTTGGTGACAAGGACAATCAATGTTCAGTGTGA
>Ca_21333 (CaNIP2-1)
MDRRTHSLVNATNDFQNHITQKQSLYPSGFPRKVLAEVIGTYLLVFVGSGSAAMNAIDENKVSK
LGASMAGGFIVTVMIYAIGHISGAHMNPAVSLAFATVSHFPWKQVPFYIAAQLTGAISASYTLK
VLLEPSKQLGATSPSGSNIQALIIEIVTTFTMVLISTAVSTDPKAIGELSGVAVGSSVCIASIV
AGPISGGSMNPARTLGPAIATSSYKGIWVYMVGPITGALLGTWSYVVIQETNKQALTTSLKLHH
EMKGIELVGDKDNQCSV

>Ca_02921 (CaNIP3-1)
ATGGAGCATAGTAATAATGAGGAAATTCCATCAACACCAGCAACACCAGGCACTCCTGGTGTAC
CTCTTTTTGGTGGGTTTAAGTCAGAGAGAAACGGGAATGGTAGTAATAAGAATAAGAAGTCTCT
CCTTAAGAATTGTAAATGCTTCAGTGTCCAAGACTGGACCATAGAAGATGGCGCTCTTCCCACA
GTCTCTTGCTCATTAATGCCTCCCCCTCCTGTCCCTCTCGCAAAAAAGATAGGAGCTGAGTTTA
TAGGGACATTCATTCTGATTTTTGCTGGGACAGCCACTGCTATTGTGAACCAAAAGACAAAAGG
GTCGGAGACTCTGATTGGATGTGCTGCTTCTTCTGGTCTTGCCGTTATGATTGTCATCCTCTCC
ACCGGTCACATCTCTGGTGCTCATCTCAACCCCGCAGTCACCATTTCCTTTGCTGCATTGAAAC
ACTTCCCATGGAAGCATGTGCCTATGTATATTGGCGCACAAGTTTTAGCATCAATATGTGCTGC
ATTTGCACTCAAAGGGGTATTTCATCCTTTCATGAGTGGTGGAGTCACTGTTCCTTCTGGAGGA
TATGGCCAAGCTTTTGCTTTAGAGTTCATTATCAGCTTTAATCTCATGTTCGTTGTCACGGCGG
TGGCCACCGACACAAGAGCTGTAGGAGAACTGGCAGGAATTGCAGTAGGAGCAACTGTGATGCT
TAATATACTCATAGCAGGGCCAATAACAGGAGGATCAATGAACCCAGTAAGAAGTCTTGGTCCA
GCTATTGCTGCAAACAACTACAAAGCCATATGGGTCTATCTATTAGCTCCCATAATTGGCGCTC
TAGGTGGTGCAGGTACTTATACCGCAGTCAAGCTTCCCCAAGAAGACGATAATGCAAAGGCTAA
TGCTCCTTCAAACCCCGCCAGCTTCAGAAGATGA
>Ca_02921 (CaNIP3-1)
MEHSNNEEIPSTPATPGTPGVPLFGGFKSERNGNGSNKNKKSLLKNCKCFSVQDWTIEDGALPT
VSCSLMPPPPVPLAKKIGAEFIGTFILIFAGTATAIVNQKTKGSETLIGCAASSGLAVMIVILS
TGHISGAHLNPAVTISFAALKHFPWKHVPMYIGAQVLASICAAFALKGVFHPFMSGGVTVPSGG

YGQAFALEFIISFNLMFVVTAVATDTRAVGELAGIAVGATVMLNILIAGPITGGSMNPVRSLGP
AIAANNYKAIWVYLLAPIIGALGGAGTYTAVKLPQEDDNAKANAPSNPASFRR

>Ca_22848 (CaNIP3-2)
ATGAAATATTCATACAAGTTTAGATCTATCCCTCTTGCTAAAAAGGTTGCAGCAGAGTTTATAG
GCACATTCATTCTAATGTTTGCTGGAATGGGAAGTGCAATAGAGAACGAAAAGGTTGAAAATTC
AGAGACACTAATTGGATGTGCTGGAGCTAGTGGACTTGCTGTTATGATCATAATTCTATCAACT
GGTCATATCTCCGGTGCTCATCTCAATCCTGCTCATTTTCCTTGGAAGAATGTACCTGTGTATA
TTGGTGCACAAATATTGGCATCAATATGTGCTGCATTTTCTCTGAAAGTGATTTTTCATCCATT
CATGAATGGTGGAGTGACGGTTCCTTCAGTAGCAATTGGTGAAGCTTTTGCATTAAAATTCATT
ATTGGCTTTAATCTCATGTTTGTTGTCACTGCTGTTGCCACCGACACAAGAGTTATGGGAGAAT
TTGCGGGAATCACGGTGGAAGCCACCGTCATGCTCAACATACTCATAGCCGGGGGACAAAATTG
TATTTTCATTTGGAATGGGGGGTGGGAAGTTAAAGTAGGGGATGCATGGTACATTTCTCTATCT
GGTGGGGTATGGCCTGCAACTGGAGGTTCAATGAATCCAGTAAGAGCACTGGGCCCAGCAATTG
CTGCAAACAACTTCAGAGACATATGGTTTTATCTCATAGCTCCTATTCTTGGAGCTCTAATTGG
GGCAGGTGCATACATTGTTGTCAAATTGCCTGATGAAGAATTTAACTGA
>Ca_22848 (CaNIP3-2)
MKYSYKFRSIPLAKKVAAEFIGTFILMFAGMGSAIENEKVENSETLIGCAGASGLAVMIIILST
GHISGAHLNPAHFPWKNVPVYIGAQILASICAAFSLKVIFHPFMNGGVTVPSVAIGEAFALKFI
IGFNLMFVVTAVATDTRVMGEFAGITVEATVMLNILIAGGQNCIFIWNGGWEVKVGDAWYISLS
GGVWPATGGSMNPVRALGPAIAANNFRDIWFYLIAPILGALIGAGAYIVVKLPDEEFN

>Ca_25553 (CaNIP3-3)
ATGAATTGTTTTACTATTGAAGAATGGAATTTGGAAGATGGTTATTTACCTAGAGTGTCTTGTG
CTTTGCCATTGCCACATGCTCCTATCCCTCTTGCTAAAAAGGTTGCAGCAGAGTTTATAGGCAC
ATTCATTCTAATGTTTGCTGGAATGGGAAGTGCAATAGAGAACGAAAAGGTTGAAAATTCAGAG
ACACTAATTGGATGTGTTGGAGCTAGTGGACTTGCTGTTATGATCATAATTCTATCAACTGGTT
ATATCTCCGGTGCTCATCTCAATCCTGCTGTTACCATCTCATTTGTTGCATTGAAGCATTTTCC
TTGGAAGAATGTACCTGTGTATATTGGTGCACAAATATTGGCATCAATATGTGCTGCATTTTCT
CTGAAAGTGATTTTTCATCCATTCATGAATGGTGGAGTGACGGTTCCTTCAGTAGCAATTGGTG
AAGCTTTTGCATTAGAATTCATTATTGGCTTTAATCTCATGTTTGTTGTCACTGATGTTGCCAC
CGACACAAGAGCTATGGGAGAATTTGCGGGAATCACGGTGGGAGCCACCATCATGCACAACATA
CTCATAGCCGGGTATGTTCAACGTTATTATTTTTAA
>Ca_25553 (CaNIP3-3)
MNCFTIEEWNLEDGYLPRVSCALPLPHAPIPLAKKVAAEFIGTFILMFAGMGSAIENEKVENSE
TLIGCVGASGLAVMIIILSTGYISGAHLNPAVTISFVALKHFPWKNVPVYIGAQILASICAAFS
LKVIFHPFMNGGVTVPSVAIGEAFALEFIIGFNLMFVVTDVATDTRAMGEFAGITVGATIMHNI
LIAGYVQRYYF

>Ca_04355 (CaNIP3-4)
ATGCCGGGATTGGAGACGGGGACACCGACGGCAGCGTCGACGCCGGCGACACCGGATACTCCGG
GAGGTCCACTATTTTCATCGGTGCGAGTTGACTCATTGGACCAACGTGAATCGTTTGGAATGGG
TAGGTGCAACAATTGTTTTCCGGGTAAAAGTAATGGAGGTTGCATCAATATCGCCGATTTCTCT
GCTAGTGTTTCTCTCACTCAAAAGATTGGAGCAGAGTTTGTAGGGACATTCATATTAATATATG

CAGCAACAGCAGGACCAATAGTGAACAACAAATACAATGGAGCAGAATCACTTATGGGAAATGC
AGCTTGTGCTGGATTAACAGTTATGTTCATTATTCTCTCAATTGGTCACATCTCAGGTGCACAT
CTCAATCCATCACTCACAATTGCGTTCGCTGCGTTTCGCCATTTCCCTTGGGCACACGTTCCTG
CCTACATTGCTGCACAAGTTTCTGCTTCTATTTGTGCTTGTTATGCTCTCAAAGTTGTTTACCA
TCCTTTCCTTACTGGTGGTGTCACTGTCCCTACTGTTAGCATTGGTCAAGCTTTTGCAACTGAG
TTTATCATCACTTTTATTCTCTTGTTTGTTGTCACTGCTGTTGCCACCGATACTCGCGCGGTTG
GTGAATTAGCTGGTATTGCTGTTGGGGCTACAGTTTTGCTCAACATTCTCATCTCAGGGCCAAC
CAGTGGTGGTTCGATGAACCCGGTGCGCACCTTAGGTCCAGCAGTTGCAGCAGGAAATTACAAA
CATATCTGGATATATTTAGTGGCGCCGACGCTCGGTGCTCTTGCTGGTTCTGGAGTTTATACGC
TTGTCAAGCTGCGTGACAACGAAGCTAATCCAGCGCAATCGGTTAGAAGCTTCCGTCGCTAG
>Ca_04355 (CaNIP3-4)
MPGLETGTPTAASTPATPDTPGGPLFSSVRVDSLDQRESFGMGRCNNCFPGKSNGGCINIADFS
ASVSLTQKIGAEFVGTFILIYAATAGPIVNNKYNGAESLMGNAACAGLTVMFIILSIGHISGAH
LNPSLTIAFAAFRHFPWAHVPAYIAAQVSASICACYALKVVYHPFLTGGVTVPTVSIGQAFATE
FIITFILLFVVTAVATDTRAVGELAGIAVGATVLLNILISGPTSGGSMNPVRTLGPAVAAGNYK
HIWIYLVAPTLGALAGSGVYTLVKLRDNEANPAQSVRSFRR

>Ca_07775 (CaNIP4-1)
ATGTTTGAAAAGCAATCATCACCCGAATTAGCTTCAAATTATGCATCAAGTAGTGGCTTGTCGG
GGGATGATAAAGAGATTGGTTATAGAGCTTCAACATTAAAGCATGGGTATCTTTTGGCTAATAA
TTCTTCTCTGCATTTCTTACCCAACAAAATTGACTTGAACTTTGCTCGAATGGTGATGGCAGAG
GTGGTGGGTACTTTTATATTGATGTTTTGTGTATGTGGAATCATTGCAAGCACACAACACCAAA
ATGGTGCAGTAGGCCTTCTGGAGTATGCAGCTACAGCAGGATTAACAGTTGTTGTCATAATTTT
CTCTATAGGCCCAATTTCTTGTGCACATGTTAACCCAGCTGTTACAATAGCCTTTGCAACAATT
GGTCAATTTCCATGGTTTAAGGTTCCAGTTTACATAATAGCACAAACAATAGGATCTTTGATGG
CAACATATATTGGTAGCCTTGTGTATGGCATAAAATCAGATGTTATGATGACACAACCACTTCA
AGGATGCAACTCTGCCTTCTGGGTGGAGGTTATTGCAACTTTCATCATCATGTTTTTGGTTTCA
GCTTTGACATCTGAACATCAATCAGTGGGCCATTTATCCGGTTTTGTTGCTGGAATTGCAATTG
GGCTTGCTGTACTAATAACAGGCCCTGTTTCAGGTGGATCAATGAATCCAGCAAGATCACTAGG
TCCAGCAATTGTATCATGGAAATTTAATTACATTTGGATATATATCATAGCCCCAAGTTCAGGA
GCTATAGCAGGAGCTCTAATGTTCCGTTTCCTACGTCTTCAAGACCAACAATGCACCTCCTCAA
ACATCACTAATGTTGGTCACCCCATACCCTTTTGTGCAAGGAGAAGTGGGTCCATGATTTTGCT
TGTAGAGAAAAATTGGAGTTTGTTCTCTAATAGAGTGGAAGGGTTCAGACAAAGATATGTTTTA
AGAACAGAAGCAATATCAGATGGTGTTTATCATAAGTTGTCACTGTAA
>Ca_07775 (CaNIP4-1)
MFEKQSSPELASNYASSSGLSGDDKEIGYRASTLKHGYLLANNSSLHFLPNKIDLNFARMVMAE
VVGTFILMFCVCGIIASTQHQNGAVGLLEYAATAGLTVVVIIFSIGPISCAHVNPAVTIAFATI
GQFPWFKVPVYIIAQTIGSLMATYIGSLVYGIKSDVMMTQPLQGCNSAFWVEVIATFIIMFLVS
ALTSEHQSVGHLSGFVAGIAIGLAVLITGPVSGGSMNPARSLGPAIVSWKFNYIWIYIIAPSSG
AIAGALMFRFLRLQDQQCTSSNITNVGHPIPFCARRSGSMILLVEKNWSLFSNRVEGFRQRYVL
RTEAISDGVYHKLSL

>Ca_22925 (CaNIP6-1)
ATGGACAATGAAGAAATTCCATCAATACCCGGAACACCTGGAAATGCAACACCTGGTACACCTG
GTGCCCCTCTTTTTGGTGGCTTAAAGCCTGAGAAAAATGGAAATCGTTCTGTTGGTAGAAATAA

ATCACTTCTGAAGAATATGAATTGTTTTGCTGTTGAAGAATGGAATTTGGAAGATGGTTCTTTA
CCTAGAGTCTCTTGTGCTTTGCCATTGCCACCTGCTCCTATCCCTCTTGCTAAAAAGGTTGGAG
CAGAGTTTATTGGCACATTCATTCTAATGTTTGCTGGAATTGGAACTGCAATAGAGAACCAAAA
GGTTGAAAATTCAGAGACACTAATAGGATGTGCTGGAGCTAGTGGACTTGCTGTTATGATCATA
ATTCTATCAACTGGTCATATCTCTGGTGCTCATCTCAATCCTGCTGTTACCATCTCATTTGCTG
CATTGAAGCATTTTCCTTGGAAGAATGTGGGAGAACTTGCGGGAATTGCGGTGGGAGCCACCGT
CATGCTCAACATACTCATAGCAGGGCCTGCAACTGGAGGTTCAATGAATCCAGTAAGAACACTG
GGCCCAACAATTGCTACAAACAACTTCAGAGGCATATGGTTATATCTCATAGCTCCTATTCTTG
GAGCTCTAACTGGGGCAGGTGCATACACTGTTGTCAAATTGCCTAATGAAGAATTTAACCCAGA
GTTAAAAGCCTCTTCTGCCCCTGGCAGTTTCAGAAGATGA

>Ca_22925 (CaNIP6-1)

MDNEEIPSIPGTPGNATPGTPGAPLFGGLKPEKNGNRSVGRNKSLLKNMNCFAVEEWNLEDGSL
PRVSCALPLPPAPIPLAKKVGAEFIGTFILMFAGIGTAIENQKVENSETLIGCAGASGLAVMII
ILSTGHISGAHLNPAVTISFAALKHFPWKNVGELAGIAVGATVMLNILIAGPATGGSMNPVRTL
GPTIATNNFRGIWLYLIAPILGALTGAGAYTVVKLPNEEFNPELKASSAPGSFRR

>Ca_02435 (CaPIP1-1)

ATGGAGAGGGAAGAAGATGTGAAAATTGGAGCAAACAAATTCTCAGAAAAGAGTGCATTAGGCA
TAGGAGCTAAAAGTGACAGCAAAGACTACAAAGAAGCACCAGCAGCACCATTGTTTGAGCCAGG
GGAGTTGAAGTCATGGTCTTTCTATAGAGCTGGAATTGCTGAGTTTGTTGCCACATTCTTGTTT
CTTTACATCAGTGTTTTAACTGTGATGGGTGTTAACAGGTCAACTTCTAAGTGTGCCTCTGTTG
GTATTCAAGGTATTGCTTGGGCTTTTGGTGGTATGATCTTTGCCCTTGTTTACTGCACTGCTGG
AATTTCAGGTGGGCACATAAATCCAGCTGTGACCTTTGGTCTATTTTTGGCTAGGAAACTGTCC
TTAACAAGGGCAGTATTCTACATAATAATGCAGTGTCTTGGAGCTATCTGTGGTGCTGGTGTGG
TGAAGGGTTTTGAAGGTAATGCACGTTATGAGATGTACAAAGGTGGAGCAAATGTTGTGAATCC
TGGATACACCAAGGGTGATGGTCTTGGAGCTGAGATTGTTGGCACTTTTGTTCTTGTCTACACT
GTCTTCTCCGCCACCGATGCCAAAAGAAACGCCAGAGACTCTCACGTTCCGATTTTGGCCCCAC
TTCCAATTGGGTTTGCAGTGTTCTTGGTTCACTTGGCAACTATTCCCATAACAGGAACAGGCAT
TAACCCAGCAAGGAGTCTTGGTGCTGCCATAATATACAACAGAGAGCATGCTTGGGATGACCAG
TGGATATTCTGGGTTGGACCTTTCATTGGAGCTGCACTTGCTGCTTTATATCACCAGATTATCA
TACGAGCTATTCCTTTCAAGGCAAGGGGTTAA

>Ca_02435 (CaPIP1-1)

MEREEDVKIGANKFSEKSALGIGAKSDSKDYKEAPAAPLFEPGELKSWSFYRAGIAEFVATFLF
LYISVLTVMGVNRSTSKCASVGIQGIAWAFGGMIFALVYCTAGISGGHINPAVTFGLFLARKLS
LTRAVFYIIMQCLGAICGAGVVKGFEGNARYEMYKGGANVVNPGYTKGDGLGAEIVGTFVLVYT
VFSATDAKRNARDSHVPILAPLPIGFAVFLVHLATIPITGTGINPARSLGAAIIYNREHAWDDQ
WIFWVGPFIGAALAALYHQIIIRAIPFKARG

>Ca_05754 (CaPIP1-2)

ATGGAGAAGGAAGAAGATGTGAAGGTAGGAGCAAGCAAATTCTCAGAGAGGCAGGCATTAGGAA
CAGCAGCTCAAAATGACAAAGACTACAAAGAGGTACCAGCAGCTCCATTGTTTGAGCCTGGTGA
GCTCAAGTCATGGTCTTTTTACAGAGCTGGAATTGCTGAGTTCATAGCCACTTTCTTGTTCCTC
TACATCACCATTTTGACTGTAATGGGTGTCAATAGATCAACCTCCAAATGTTCCTCTGTTGGCA
TTCAAGGTATTGCTTGGTCTTTTGGGGGTATGATCTTTGCCCTTGTCTACTGCACTGCTGGAAT
TTCAGGTGGACACATAAACCCTGCTGTGACATTTGGACTATTTTTGGCAAGGAAGCTATCTCTT

ACAAGAGCTATATTCTACATTGTGATGCAATGTCTTGGTGCTATTTGTGGTGCTGGTGTGGTGA
AGGGTTTTGAAGGTAATGCTAGATATGAGTTGTTCAAAGGTGGAGCTAATTTTGTGAATGCTGG
TTACACTAAAGGTGATGGCCTTGGAGCTGAGATTGTTGGTACTTTTGTTCTTGTCTACACTGTT
TTCTCTGCCACTGATGCCAAGAGAAATGCTAGAGACTCTCATGTTCCTCTTTTGGCTCCTCTTC
CAATTGGTTTTGCTGTGTTCTTGGTCCACTTGGCAACTATTCCCATCACAGGAACTGGCATTAA
CCCAGCTAGGAGTCTTGGAGCTGCTATCATCTTTAACAGGGACTTTGCATGGGATGACCATTGG
ATATTCTGGGTTGGACCATTCATTGGAGCTGCTCTTGCTGCTATGTATCACCAGATTGTTATTA
GAGCCATTCCTTTCAAGACAAGGGCTTGA

>Ca_05754 (CaPIP1-2)

MEKEEDVKVGASKFSERQALGTAAQNDKDYKEVPAAPLFEPGELKSWSFYRAGIAEFIATFLFL
YITILTVMGVNRSTSKCSSVGIQGIAWSFGGMIFALVYCTAGISGGHINPAVTFGLFLARKLSL
TRAIFYIVMQCLGAICGAGVVKGFEGNARYELFKGGANFVNAGYTKGDGLGAEIVGTFVLVYTV
FSATDAKRNARDSHVPLLAPLPIGFAVFLVHLATIPITGTGINPARSLGAAIIFNRDFAWDDHW
IFWVGPFIGAALAAMYHQIVIRAIPFKTRA

>Ca_10319 (CaPIP1-3)

ATGGAGGCAAAGGAACAAGATGTCTCATTGGGAGCAAACAAATACCCAGAGAGACAACCAATTG
GTATAGCAGCTCAGAGCCAAGACGATGGAAAAGACTACAAGGAACCACCACCTGCACCATTGTT
TGAACCATCTGAACTCACATCATGGTCTTTCTACAGAGCTGGGATAGCTGAGTTTGTGGCCACT
TTTCTGTTTCTTTACATAACCATCTTGACGGTTATGGGTGTGAACAGATCTGACTCAAAATGCA
AATCTGTTGGTATTCAAGGGATTGCTTGGTCTTTTGGTGGCATGATATTTGCCCTCGTTTACTG
TACTGCCGGAATCTCTGGGGGTCACATAAACCCAGCAGTGACATTCGGTTTGTTCTTGGCGAGG
AAATTGTCGTTGACACGGGCGGTGTTTTACATCGTGATGCAGGTGCTTGGTGCTATCTGTGGAG
CTGGTGTTGTTAAGGGTTTTGAGGGAAAGACCTTGTACGGTAAATTCCACGGTGGTGCTAACTT
TGTTGCTCCTGGTTACACAAAAGGAGATGGACTTGGTGCTGAGATTATTGGTACTTTTGTTCTT
GTCTACACCGTTTTCTCCGCCACTGATGCCAAACGTAGCGCTAGAGACTCTCACGTTCCTATTT
TGGCACCCTTGCCAATTGGGTTCGCTGTCTTTTTGGTGCACTTGGCCACTATCCCAATCACTGG
AACTGGTATCAACCCTGCTAGGAGTCTTGGTGCTGCCATTATCTTCAACAAAGACCTTGGCTGG
GATGATCAATGGATTTTCTGGGTTGGGCCATTCATTGGTGCAGCTCTTGCAGCCCTTTACCACC
AAGTTGTCATCAGAGCCATTCCTTTCAAATCAAGTTAA

>Ca_10319 (CaPIP1-3)

MEAKEQDVSLGANKYPERQPIGIAAQSQDDGKDYKEPPPAPLFEPSELTSWSFYRAGIAEFVAT
FLFLYITILTVMGVNRSDSKCKSVGIQGIAWSFGGMIFALVYCTAGISGGHINPAVTFGLFLAR
KLSLTRAVFYIVMQVLGAICGAGVVKGFEGKTLYGKFHGGANFVAPGYTKGDGLGAEIIGTFVL
VYTVFSATDAKRSARDSHVPILAPLPIGFAVFLVHLATIPITGTGINPARSLGAAIIFNKDLGW
DDQWIFWVGPFIGAALAALYHQVVIRAIPFKSS

>Ca_12502 (CaPIP1-4)

ATGGAAGCGAAGGAACAAGATGTGTCTTTGGGAGCCAACAAATTCCCAGAGAGACAGCCGCTTG
GGATTGCGGCTCAGAGCCAAGATGAACCGAAGGATTACCAGGAGCCACCACCGGCTCCACTTTT
TGAGCCGTCGGAGCTGACTTCATGGTCTTTCTACAGAGCTGGGATAGCTGAGTTCATTGCCACT
TTTCTTTTTCTTTACATCACGGTTTTAACTGTCATGGGTGTTGTTAGAGAAAGTTCCAAGTGTA
AAACCGTTGGTATTCAAGGAATTGCTTGGGCTTTTGGTGGCATGATCTTCGCTCTCGTTTATTG
TACTGCTGGAATCTCAGGTGGTCACATAAATCCAGCGGTGACATTTGGGTTATTTTTGGCGAGG
AAGTTGTCATTGACTAGGGCACTGTTCTACATGGTGATGCAAGTGTTGGGTGCTATATGTGGTG

CTGGTGTTGTGAAAGGGTTTGAAGGAAAAACAAGATTTGGTGATCTTAAAGGTGGTGCCAACTT
TGTGAACCCTGGTTACACCAAAGGTGATGGACTTGGTGCTGAAATTGTTGGCACTTTCATTCTT
GTTTACACCGTTTTCTCAGCCACTGATGCTAAGCGTAGCGCCAGAGACTCTCATGTCCCTATTT
TGGCACCATTGCCAATTGGGTTCGCTGTGTTCTTGGTGCATTTGGCTACAATCCCAATTACTGG
AACCGGTATTAATCCAGCTCGTAGTCTCGGTGCTGCAATTATCTTCAACAAGGACCTTGGTTGG
GATGATCATTGGATCTTCTGGGTGGGACCATTTATTGGAGCAGCTCTGGCAGCTCTATACCACC
AAGTTGTAATCAGAGCCATTCCCTTTAAGTCCAAGTAA

>Ca_12502 (CaPIP1-4)

MEAKEQDVSLGANKFPERQPLGIAAQSQDEPKDYQEPPPAPLFEPSELTSWSFYRAGIAEFIAT
FLFLYITVLTVMGVVRESSKCKTVGIQGIAWAFGGMIFALVYCTAGISGGHINPAVTFGLFLAR
KLSLTRALFYMVMQVLGAICGAGVVKGFEGKTRFGDLKGGANFVNPGYTKGDGLGAEIVGTFIL
VYTVFSATDAKRSARDSHVPILAPLPIGFAVFLVHLATIPITGTGINPARSLGAAIIFNKDLGW
DDHWIFWVGPFIGAALAALYHQVVIRAIPFKSK

>Ca_08491 (CaPIP2-1)

ATGGCAAAAAACGTTGAGGTTGCAGAGCGTGGCTCTTTCTCTAACAAAGACTACCATGACCCTC
CTCCAGCACCGTTCATTGATGCTGCTGAACTAACAAAATGGTCTTTTTACAGAGCACTCATTGC
TGAGTTCATTGCAACTTTGCTTTTTCTTTATGTTACTGTTTTGACTGTTATTGGATATAGTATT
CAGACTGATATTAAAGCAGGTGGTGATGTTTGTGGTGGTGTTGGTATTCTTGGTATTGCTTGGG
CTTTTGGTGGCATGATTTTTGTACTTGTTTATTGCACTGCTGGAATTTCAGGGGGTCACATTAA
CCCAGCAGTGACATTTGGGTTATTTTTGGCTCGCAAGGTGTCTTTAATCAGAGCAATTATGTAC
ATAGTAGCTCAGTGTTTGGGGGCAATTTGTGGAGTTGGGTTGGTGAAGGCTTTCCAAAGTGCTT
ACTTTGACAGGTATGGTGGTGGAGCTAATTTTCTCCATGATGGGTATAGTACTGGTGTTGGATT
AGGTGCTGAGATTGTTGGAACTTTTGTCTTGGTATACACTGTGTTTTCTGCTACCGATCCTAAG
AGAAGTGCTAGAGATTCTCATGTTCCGGTTTTGGCTCCACTTCCCATTGGATTTGCTGTATTCA
TGGTTCACTTGGCCACTATCCCTGTCACTGGCACTGGCATTAATCCTGCTAGAAGTCTTGGTTC
TGCTGTTATCCTCAACCAAGATAAGCCTTGGGATGACCATTGGATCTTTTGGGTAGGACCATTT
GCTGGGGCAGCCATTGCAGCTTTCTACCACCAATTCATCTTAAGAGCAGGTGCAGTTAAAGCTC
TTGGATCATTCAGGAGTAACCCCACTGTTTGA

>Ca_08491 (CaPIP2-1)

MAKNVEVAERGSFSNKDYHDPPPAPFIDAAELTKWSFYRALIAEFIATLLFLYVTVLTVIGYSI
QTDIKAGGDVCGGVGILGIAWAFGGMIFVLVYCTAGISGGHINPAVTFGLFLARKVSLIRAIMY
IVAQCLGAICGVGLVKAFQSAYFDRYGGGANFLHDGYSTGVGLGAEIVGTFVLVYTVFSATDPK
RSARDSHVPVLAPLPIGFAVFMVHLATIPVTGTGINPARSLGSAVILNQDKPWDDHWIFWVGPF
AGAAIAAFYHQFILRAGAVKALGSFRSNPTV

>Ca_12039 (CaPIP2-2)

ATGGCTAAAGACATTCAAACCGAACCTCAAACTGCTTTGCCAAACAAAGACTACCAAGATCCAC
CACCTGCTCCACTCTTCGACACCTCTGAACTCTCCCAGTGGTCTTTCTACAGAGCTCTCATCGC
CGAGTTCGTTGCCACTCTCCTCTTCCTCTACGTCACCGTCGCCACTGTCATTGGCTACAACTCT
CAAACCGACCCAGCTCATAACGGCACCGCCTGTGACGGTGTCGGCATCCTCGGCATCGCTTGGG
CCTTCGGTGGCATGATTTTTGTCCTTGTCTACTGCACCGCCGGCATATCTGGGGGACACATAAA
TCCGGCGGTGACATTTGGGTTGTTTCTGGCGAGAAAGGTATCGTTGATTAGAGCAATATTATAC
ATGGTGGTTCAATGTTTAGGAGCAATATGTGGTGTTGGGTTAGTAAAAGGTTTTCAGAAAAGTT
ACTACAATAGGTACAAAGGTGGTGCAAATATGTTATCTAATGGGTACAGTAAGGGAACAGGGTT

AGGTGCTGAGATCATTGGAACCTTTTTTCTTGTCTACACCGTTTTCTCTGCCACTGATCCTAAG
AGAAATGCTAGAGACTCTCATGTTCCCGTTTTGGCACCACTTCCAATTGGTTTTGCTGTGTTTG
TGGTTCACCTTGCTACTATTCCTATCACTGGAACTGGTATCAACCCTGCTAGAAGCCTTGGAGC
TGCTGTCATATACAACAATCACAAGGCATGGGATCACCATTGGATATTCTGGGTTGGACCCTTT
ATTGGTGCTGCCATTGCTGCAATTTACCACCAGTTTGTGTTGAGAGCACAAGCGGCAAAGGCTT
TGGGTTCTTTCAAGAGTTCTTCAAACCTTTAA

>Ca_12039 (CaPIP2-2)

MAKDIQTEPQTALPNKDYQDPPPAPLFDTSELSQWSFYRALIAEFVATLLFLYVTVATVIGYNS
QTDPAHNGTACDGVGILGIAWAFGGMIFVLVYCTAGISGGHINPAVTFGLFLARKVSLIRAILY
MVVQCLGAICGVGLVKGFQKSYYNRYKGGANMLSNGYSKGTGLGAEIIGTFFLVYTVFSATDPK
RNARDSHVPVLAPLPIGFAVFVVHLATIPITGTGINPARSLGAAVIYNNHKAWDHHWIFWVGPF
IGAAIAAIYHQFVLRAQAAKALGSFKSSSNL

>Ca_14568 (CaPIP2-3)

ATGGCTAAAGATGTTGAGGTTCAAGAACATGGTGAATTCTCAGCTAAAGATTACCAAGATCCAC
CACCAGCACCATTGATTGATTTTGATGAGTTAACAAAGTGGTCTTTTTATAGAGCTCTTATAGC
TGAATTTGTAGCAACACTTTTGTTCCTTTATGTCACAATTTTGACCATTATTGGTTATAGTCAT
CAGAGTGATCCTAAAGCTGGTGGTACTGATTGTGATGGTGTTGGGATTTTGGGTATTGCTTGGG
CTTTTGGTGGCATGATTTTCATCCTTGTTTACTGCACTGCTGGTATTTCTGGAGGGCACATAAA
TCCGGCAGTGACATTCGGACTATTCGTAGGAAGAAAGGTGTCGTTGCTGAGGGCGGTGTTTTAC
ATGGCAGCGCAGTGTGCCGGTGCGATCTCCGGTACCGGACTAGCAAAGGGTTTCCAAAAAGCAT
ACTTTGACAGGTATGGAGGTGGTGCTAACTTTGTTCATGATGGTTACAACAAAGGTACAGCTTT
GGGTGCTGAGATTATTGGTACCTTTGTTCTTGTCTACACTGTCTTCTCTGCCACTGATCCAAAA
AGAAACGCTAGGGACTCCCATGTTCCTGTTTTGGCACCACTACCCATTGGATTTGCTGTTTTCA
TGGTTCACTTGGCTACTATCCCTATTACCGGTACCGGTATTAACCCGGCAAGAAGTTTCGGATC
AGCCGTAATCTACAACGAAGGCAAAATTTGGGATGACCAGTGGATATTCTGGGTTGGACCAATT
ATTGGAGCTACAGTGGCTGCAATATACCACCAATACATTCTTAGAGGATCAGCCATTAAAGCTC
TTGGATCCTTCAGAAGCAATGCTTAA

>Ca_14568 (CaPIP2-3)

MAKDVEVQEHGEFSAKDYQDPPPAPLIDFDELTKWSFYRALIAEFVATLLFLYVTILTIIGYSH
QSDPKAGGTDCDGVGILGIAWAFGGMIFILVYCTAGISGGHINPAVTFGLFVGRKVSLLRAVFY
MAAQCAGAISGTGLAKGFQKAYFDRYGGGANFVHDGYNKGTALGAEIIGTFVLVYTVFSATDPK
RNARDSHVPVLAPLPIGFAVFMVHLATIPITGTGINPARSFGSAVIYNEGKIWDDQWIFWVGPI
IGATVAAIYHQYILRGSAIKALGSFRSNA

>Ca_02533 (CaPIP2-4)

ATGGGAAAAGATGTTGAAGTTCAAGAACAAGGTGGTGAATATTCAGCTAAGGATTATCAAGATC
CACCACCAGCACCTTTGTTTGATCCAGCAGAGTTAACAAAGTGGTCTTTGTATAGAGCTGTGAT
AGCAGAGTTCATAGCAACACTTCTGTTCCTTTACATCACTGTGTTGACCATTATTGGTTATAGT
AGACAAACTGATACCACTATTACAGGTAACACTGAATGTGATGGTGTTGGAGTTTTGGGTATTG
CTTGGGCTTTTGGTGGTATGATCTTCGTCCTTGTTTACTGCACCGCCGGTATCTCTGGAGGACA
CATAAATCCAGCAGTGACATTTGGGCTGTTTTTGGGACGCAAGGTGTCTTTAATAAGGGCTGTA
CTATACATAATAGCACAATGTTTAGGTGCAATTTGTGGTGCTGGTCTTGCAAAAGGGTTCCAAA
AATCATACTACAATAGGTACCATGGAGGTGTTAATTTGGTGTCTGATGGTTATAGTAAAGGCAC
TGCTTTGGGTGCTGAAATTATTGGTACCTTTGTTTTAGTTTACACTGTTTTCTCAGCCACTGAT

CCTAAGAGAAGTGCTAGGGATTCACATGTTCCTGTATTGGCACCACTTCCTATTGGATTTGCTG
TCTTCATGGTTCATTTGGCAACAATCCCTGTTACCGGTACCGGAATTAACCCTGCTAGGAGTTT
TGGACCCGCTGTTATCTACAACAACGACAAAGCTTGGGATGATCAGTGGATTTACTGGGTTGGA
CCATTTATTGGAGCTGCAATAGCTGCATTCTATCACCAATTTATTCTAAGAGCAACAGCAATAA
AGGCTCTTGGATCCTTCAGGAGCAACCCTTAA
>Ca_02533 (CaPIP2-4)
MGKDVEVQEQGGEYSAKDYQDPPPAPLFDPAELTKWSLYRAVIAEFIATLLFLYITVLTIIGYS
RQTDTTITGNTECDGVGVLGIAWAFGGMIFVLVYCTAGISGGHINPAVTFGLFLGRKVSLIRAV
LYIIAQCLGAICGAGLAKGFQKSYYNRYHGGVNLVSDGYSKGTALGAEIIGTFVLVYTVFSATD
PKRSARDSHVPVLAPLPIGFAVFMVHLATIPVTGTGINPARSFGPAVIYNNDKAWDDQWIYWVG
PFIGAAIAAFYHQFILRATAIKALGSFRSNP

>Ca_04707 (CaPIP2-5)
ATGTCGAAGGAAGTGAGCGAAGAAGGTCACTTGCAGACTCATCATCATGGTGGTAAAGACTACG
TAGACCCACCACCAGCACCCCTCCTCGATTTCGCTGAGATTAAACTCTGGTCCTTTTACAGAGC
TCTCATCGCCGAGTTCATAGCCACACTCCTCTTCCTCTATGTCACCGTCGCCACCGTCATTGGT
CACAAAAAACAAACCGGTCCCTGCGACGGTGTTGGGCTTCTAGGCATAGCCTGGTCCTTCGGTG
GCATGATCTTCGTCCTTGTCTACTGCACCGCCGGCATCTCCGGTGGACACATAAACCCTGCGGT
TACATTTGGGTTGTTTTTGGCACGTAAGGTGTCTCTGATACGTGCGGTGTTATACATGGTAGCA
CAGTGTTTGGGTGCTATTTGTGGTGTTGGATTGGTGAAAGCTTTGATGAAGCAACCTTACAACA
ACCTTGGTGGCGGTGCTAACTCTGTTGCTTCTGGTTACTCCAAAGGAAGTGCACTTGGTGCTGA
AATGATCGGAACTTTTGTACTTGTGTACACAGTGTTCTCTGCCACAGACCCAAAGAGAAACGCA
CGTGATTCGCATGTGCCTGTTTTGGCTCCTTTGCCAATTGGTTTTGCTGTTTTCATGGTTCATT
TGGCAACTATACCAATCACCGGTACAGGTATCAACCCTGCAAGGAGCTTCGGTGCTGCTGTTAT
CTTCAACAATGCTAAAGTTTGGGATGACCATTGGATCTTCTGGGTTGGGCCTTTCGTGGGAGCT
TTAGCGGCGGCTGCGTACCATCAATACATTCTTAGAGCGGCGGCTATCAAAGCGTTGGGGTCTT
TCAGGAGCAATCCCACCAACTAG
>Ca_04707 (CaPIP2-5)
MSKEVSEEGHLQTHHHGGKDYVDPPPAPLLDFAEIKLWSFYRALIAEFIATLLFLYVTVATVIG
HKKQTGPCDGVGLLGIAWSFGGMIFVLVYCTAGISGGHINPAVTFGLFLARKVSLIRAVLYMVA
QCLGAICGVGLVKALMKQPYNNLGGGANSVASGYSKGSALGAEMIGTFVLVYTVFSATDPKRNA
RDSHVPVLAPLPIGFAVFMVHLATIPITGTGINPARSFGAAVIFNNAKVWDDHWIFWVGPFVGA
LAAAAYHQYILRAAAIKALGSFRSNPTN

>Ca_08262 (CaSIP1-1)
ATGGTCAGTGCTATAAAGTCAGCAATTGGAGATGCAGTGTTGACTTTCATGTGGGTGTTTTGTT
CTTCCATGTTGGGGATAGTTACAAATGCTATAACCAAATCCCTCGATCTTCAAGACGTTTCGTA
CAATGGTTTTCCATACCCTTCTTTCATTGTCATCACTACGCTTGTTTTTCTCCTTGTTTTTTTG
TTTACCTTAATTGGTAGTGCAATGGGTGGTGCTAGCTTTAACCCTACCGGGACTGCTTCGTTTT
ATGCTGTTGGTCTTGGTTCTGATACGCTTTTCTCAATGGCTCTTCGTTTCCCTGCTCAGGCACT
TGGTGCTGCTGGCGGTGCAATGGCAATTTCGGAGTTGATTCATCCGAAATACAAGCACATGATC
GGGGGACCTTCTTTGAAAGTGGACTTGCATACTGGTGCTGTTGCTGAATTGGTTTTGACATTTG
TGATTACTTTTATTGTCCTCTGCATATTCCTCAAGGGCCCTCGTAACGAGTTAATGAAGATTTG
GTTGCTGGCCATGTCAACCGTCACTTTGGTCATGGCTGGTGGTGCTTACACTGGTCCATCCATG
AACCCGGCCAATGCATTTGGTTGGGCATACATAAACAACCGGCACAACACATGTGACCAATTCT

ATGTATACTGGATTTGTCCTTTCACTGGAGCAATATTGGCTGCTTGGCTATTTCGCGCTATCTT
CCCCCCACCAGAAGTAAAACAGAAAAAAGCATGA
>Ca_08262 (CaSIP1-1)
MVSAIKSAIGDAVLTFMWVFCSSMLGIVTNAITKSLDLQDVSYNGFPYPSFIVITTLVFLLVFL
FTLIGSAMGGASFNPTGTASFYAVGLGSDTLFSMALRFPAQALGAAGGAMAISELIHPKYKHMI
GGPSLKVDLHTGAVAELVLTFVITFIVLCIFLKGPRNELMKIWLLAMSTVTLVMAGGAYTGPSM
NPANAFGWAYINNRHNTCDQFYVYWICPFTGAILAAWLFRAIFPPPEVKQKKA

>Ca_19143 (CaSIP1-2)
ATGGTTAATGCAATAAAGGCAGCAATTGGAGATGCAGTATTGACATTCACATGGCTGTTTATAT
CATCCACACTTGGATTAGTCACAAATGAAATTATGAAATTTTTTGATCTTCAATTTGTGACATA
TAATGGTTTAAATTATCCTTTTATTATAATCACAATATTACTAATATTCATTACTATAATTACT
TTCACTACAATTGGTAATGCTTTGGGTGGTGCAAGTTTCAATCCCACAGGCAATGCTTCTCTTT
ATGCAGCTGGTCTTGGTTCTGATACCCTTTTCTCTATGGCTCTTCGTTTCCCTGCTCAGGCATT
AGGTGCAGTGGGTGGTGCTGTTGCAATTATGGAGGTGATTCCGCCGAAATATCGGCATATGATT
GGAGGACCTGCTTTGAAGGTTGATTTGCATACTGGTGCTATTGCAGAAGGAGTATTGACATTTG
TAATCACTTTTGCTGTTCTCTTCATCATGCTTCGGGGTCCTCGTAGTGAGTTGGTGAAGACTTT
GTTGACGGCCATCTTGACCGTGGCTTTGATCATCGTCGGTTCTGCTTACACCGGACCATCCATG
AATCCTGTCCTTGTGGGTGATCAAGTTTTTTGCTTTCACCAGTTGTTATCATTATTTTTCTTGT
CTGAATAA
>Ca_19143 (CaSIP1-2)
MVNAIKAAIGDAVLTFTWLFISSTLGLVTNEIMKFFDLQFVTYNGLNYPFIIITILLIFITIIT
FTTIGNALGGASFNPTGNASLYAAGLGSDTLFSMALRFPAQALGAVGGAVAIMEVIPPKYRHMI
GGPALKVDLHTGAIAEGVLTFVITFAVLFIMLRGPRSELVKTLLTAILTVALIIVGSAYTGPSM
NPVLVGDQVFCFHQLLSLFFLSE

>Ca_08136 (CaSIP2-1)
ATGGAACGAAGTAAATTGATTATTGTATCAGATTTTGTTATGTCTTTCATGTGGGTATGCTCTG
GTGTTCTCGTTCGATTATTCGTCTTCAAAGTTCTTGCCTTTTCTCATACCCATATCGCTGAGAT
TGTCAAAATTGTTTTTTCCATAGCCAACATGTTCCTCTTTGCTTTCCTTGCTAAGGTTTCTCGT
GGTGGGGCCTACAATCCTCTCACTGTTTTGGCTGATGCTTTCTCTGGAGATTTTCATAACTTCA
TTTTTTGTGTTGGTTCCAGAATTCCTGCTCAGGTGGTTGGATCTATTGTTGGTGTAAAATTTCT
TATTGATACCATTCCCGAAGTAGGACGGGGACCGCGTTTGAATGTTGACATTCATCGGGGAGCA
TTGACAGAAGGATTACTAACATATGCAATTGTAACCATTTCACTTGGACTTGCCGCAACAAAAA
TCCATGGAAGTTTCTTCATGAAGACTTGGATTTCCAGCCTCTCCAAGTTAACACTTCATATACT
TGGTTCTGATCTTACCGGTGGTTGTATGAACCCCGCAGCTGTAATGGGATGGGCTTATGCTCGA
GGCGATCACATAACAAAGGAGCACATTCTTGTATACTGGCTTGCCCCCATAGAGGCAACTATTT
TGGCAGTGTGGACATTTAAATTGCTCGTACGACCCGTAAAAGAAGATAAAACAGGCTCAAAAAG
TAAATCAGATTGA
>Ca_08136 (CaSIP2-1)
MERSKLIIVSDFVMSFMWVCSGVLVRLFVFKVLAFSHTHIAEIVKIVFSIANMFLFAFLAKVSR
GGAYNPLTVLADAFSGDFHNFIFCVGSRIPAQVVGSIVGVKFLIDTIPEVGRGPRLNVDIHRGA

LTEGLLTYAIVTISLGLAATKIHGSFFMKTWISSLSKLTLHILGSDLTGGCMNPAAVMGWAYAR
GDHITKEHILVYWLAPIEATILAVWTFKLLVRPVKEDKTGSKSKSD

>Ca_00723 (CaTIP1-1)
ATGCCGATCAGAAATATTGCCATCGGAAATCCACAAGAGGCAACTCACCCAGACACCTTGAAAG
CTGGTCTCGCTGAGTTTATCTCAACCTTCATCTTCGTCTTCGCCGGCTCCGGTTCAAGCATCGC
TTACAACAAGCTAACCAACGACGGTGCTGCTACTCCCTCCGGCCTCATCTCCGCCGCTATCGCT
CATGCGTTTGCTCTCTTTGTTGCTGTTTCCGTAGGTGCCAATATCTCCGGTGGTCATGTTAACC
CCGCCGTCACTTTCGGTGCCTTCGTTGGTGGTAACATCACCCTCCTCCGTGGTATCGTTTACAT
TATCGCTCAACTCCTCGGATCCATCGTCGCTTCCTTGCTCCTCGTCTTCGCTACTGGTTTGTCT
GTTCCAGCATTCTCACTTTCAGCTGGAGTTGGAGTGGGTCCCGCTTTGGTGTTGGAGATCGTGT
TGACCTTCGGTTTGGTGTACACCGTGTATGCTACTGCCGTTGACCCAAAGAAGGGTAATATTGG
AATTATTGCACCAATTGCAATTGGTTTCATTGTTGGTGCTAACATTTTGGTTGGAGGAGCCTTC
ACCGGAGCATCCATGAACCCAGCCGTGTCATTCGGCCCAGCTGTTGTAAGCTGGAGCTGGTCCA
ACCACTGGATCTACTGGGCTGGGCCACTTATCGGTGGTGGGCTTGCTGGGCTTATCTACGAGGT
CGTCTTCATTAGCCACACCCACGAGCAACTCCCTACCACTGACTACTAG
>Ca_00723 (CaTIP1-1)
MPIRNIAIGNPQEATHPDTLKAGLAEFISTFIFVFAGSGSSIAYNKLTNDGAATPSGLISAAIA
HAFALFVAVSVGANISGGHVNPAVTFGAFVGGNITLLRGIVYIIAQLLGSIVASLLLVFATGLS
VPAFSLSAGVGVGPALVLEIVLTFGLVYTVYATAVDPKKGNIGIIAPIAIGFIVGANILVGGAF
TGASMNPAVSFGPAVVSWSWSNHWIYWAGPLIGGGLAGLIYEVVFISHTHEQLPTTDY

>Ca_16712 (CaTIP1-2)
ATGCCGATTTCTAGAATTGCAATTGGAAGTCCTTCTGAGTTTGGCAAAGCTGATGCACTTAAGG
CTGCACTTGCTGAGTTCATCTCAATGCTCATCTTTGTCTTTGCAGGGGAAGGCTCTGGCATGGC
ATATAATAAGCTTACAAACAATGGTGCAGCAACACCAGCTGGGTTGGTAGCTGCATCACTGTCA
CATGCTTTTGCTCTTTTTGTTGCTGTCTCTGTTGGTGCTAACATCTCTGGTGGACATGTCAATC
CTGCTGTTACATTTGGTGCCTTCATTGGTGGCCACATTACACTTATTAGAGGCCTTTTGTATTG
GATTGCTCAGTTGCTTGGTTCTGTTGTTGCTTGCTTGCTCCTTAAAATTGCCACTGGTGGATTG
GAAACATCTGCATTCTCATTGTCTTCAGGAGTGGGAGCAACAAATGCACTTGTGTTTGAGATTG
TGATGACTTTTGGTTTGGTTTACACAGTGTATGCCACTGCAGTAGACCCAAAGAAGGGTGACCT
TGGAACAATTGCTCCAATTGCCATTGGTTTCATTGTAGGGGCCAACATATTAGCAGGTGGTGCT
TTTGATGGTGCCTCCATGAACCCAGCAGTCTCTTTTGGGCCTGCTGTTGTTAGCTGGACATGGA
CTAATCATTGGGTCTATTGGGTCGGCCCATTGATCGGTTCTGCTGTCGCTGCAGTTGTGTATGA
GATTTTCTTCATCACCCCTAGCTCTTATGAACAGTTACCGGTCGCAGATTATTAG
>Ca_16712 (CaTIP1-2)
MPISRIAIGSPSEFGKADALKAALAEFISMLIFVFAGEGSGMAYNKLTNNGAATPAGLVAASLS
HAFALFVAVSVGANISGGHVNPAVTFGAFIGGHITLIRGLLYWIAQLLGSVVACLLLKIATGGL
ETSAFSLSSGVGATNALVFEIVMTFGLVYTVYATAVDPKKGDLGTIAPIAIGFIVGANILAGGA
FDGASMNPAVSFGPAVVSWTWTNHWVYWVGPLIGSAVAAVVYEIFFITPSSYEQLPVADY

>Ca_18630 (CaTIP1-3)
ATGGCGATATACAGAATAGCAATTGGATCGCCTAGAGAGGCTAGCAATCCAGCTGCCATTAGAG
CAGCTTTTGCAGAGTTCTTCTCTATGCTCATTTTTGTTTTTGCTGGTCAAGGCTCTGGAATGGC
TTACAGCAAACTTACAAACAATGGAGCTGCAACTCCTGAAGGTCTCATAGTTGCATCATTATCT
CATGCATTTGGGTTGTTTGTTGCTGTTTCTGTTGGGGCAAACATTTCTGGTGGTCATGTCAACC
CTGCAGTCACATTTGGTGCTTTCATTGGTGGAAATATAACCTTCTTGAGAAGTATTTTGTATTG
GATTGCTCAGTTACTTGGTTCAGTTGCTGCTTGCATTATTCTCAATTCTTGCACCGGTGGAATG
GAAACATCAGCTTTCTCTCTATCCTCAGGTGTGTCTGTGTGGAATGCATTAGTATTTGAGATTG
TGATGACATTTGGATTGGTATATACAGTGTATGCAACAGCAATAGACCCAAAGAAAGGGAATTT
GGGTGTTGTAGCTCCATTAGCAATTGGTTGTGTTGTTGGTGCTAATATATTGGTTGGTGGTGTT
TTTGATGGTGCATCCATGAACCCAGCTGTGTCCTTTGGGCCTGCTGTTGTTAGTGGGGTATGGA
CTCATCATTGGGTCTATTGGGTTGGCCCATTTATTGGTTCAGCCACTGCTGCTATTCTCTATGA
TAATATCTTTATTGGTGATGATGCTCATCAACCCCTTTCAAATAGTGACTTCTAG
>Ca_18630 (CaTIP1-3)
MAIYRIAIGSPREASNPAAIRAAFAEFFSMLIFVFAGQGSGMAYSKLTNNGAATPEGLIVASLS
HAFGLFVAVSVGANISGGHVNPAVTFGAFIGGNITFLRSILYWIAQLLGSVAACIILNSCTGGM
ETSAFSLSSGVSVWNALVFEIVMTFGLVYTVYATAIDPKKGNLGVVAPLAIGCVVGANILVGGV
FDGASMNPAVSFGPAVVSGVWTHHWVYWVGPFIGSATAAILYDNIFIGDDAHQPLSNSDF

>Ca_19377 (CaTIP1-4)
ATGGCAATCTATAGAATTGCAATTGGGTCTCCTGGAGAGGCTGGTCAACCTGATGCAATTAGAG
CTGCATTTGCTGAATTTTTTTCTATGCTCATTTTTGTTTTTGCAGGGGAAGGCTCTGGCATGGC
TTACAACAAACTTACCAATAATGGACCTGCAACACCTGCTGGTCTCATAGCTGCATCACTGTCA
CATGCATTTGGACTTTTTGTTGCTGTTTCTGTTGGAGCAAATGCCTCTGGTGGTCATGTCAACC
CAGCAGTTACATTTGGTGCTTTCATGGGAGGAAACATTACCCTTTTGAGAAGTATTTTATATTG
GATTGCACAATTACTTGGTTCAGTTGTTGCTTGCATTCTTCTCAAGTCTGCTACTGGTGGAATG
GAGACATCAGCTTTTGCTATATCTTCAGATATATCAGTTTGGAATGCACTAGTTTTTGAAATTG
TGATGACATTTGGATTGGTATATACAGTTTATGCAACAGCAGTAGATCCAAAAAAAGGAAATGT
AGGAGTTGTTGCTCCAATTGCAATTGGTTTTATTGTTGGTGCAAATATCTTAGCTGGTGGAGCA
TTTGATGGTGCATCAATGAATCCAGCTGTCTCATTTGGGCCTGCAGTTGTTAGTTGGACATGGA
CTCATCATTGGGTCTATTGGGTTGGCCCATTTACTGGTGCAGCAATTGCTGCAATCATTTATGA
TAATATCTTTATTGGTGACGATGGTCATGAACCCCTCAGTGATTTTTAG
>Ca_19377 (CaTIP1-4)
MAIYRIAIGSPGEAGQPDAIRAAFAEFFSMLIFVFAGEGSGMAYNKLTNNGPATPAGLIAASLS
HAFGLFVAVSVGANASGGHVNPAVTFGAFMGGNITLLRSILYWIAQLLGSVVACILLKSATGGM
ETSAFAISSDISVWNALVFEIVMTFGLVYTVYATAVDPKKGNVGVVAPIAIGFIVGANILAGGA
FDGASMNPAVSFGPAVVSWTWTHHWVYWVGPFTGAAIAAIIYDNIFIGDDGHEPLSDF

>Ca_02797 (CaTIP2-1)
ATGGCTCGCATATCATTTGGAGACTTTGAGGATTCTTTCAGTTCCAGCTCTATTAGGGCATATA
TTGCTGAGTTCATCTCAACCTTACTCTTTGTTTTTGCTGGTGTTGGTTCTACTAGAGCCTTTGA
TAAGTTGACATCAGATGCAGCACTTGATCCAGCTGGGTTATTATCAATTGCTGTATGTCATGGT
TTTGCTCTCTTTGTTGCTGTTTCTGTTGGAGCTAACATTTCTGGTGGCCATGTCAATCCAGCTG
TCACCTTTGGAATGGCTCTTGGTGGACAAATCACCATCCTCACTTCTCTCTTCTATTGTATTGC

ACAGTTTCTTGGCTCCATAGCTGCATGCTTACTCCTCAAGTTTGTAACTGGAAGCTTGACAAAT
ATTCCAATCCATAGCATAGGTGCAGGTGTTGGAGTTGGAGAAGGAGTAGTAACAGAGATAGTGA
TAACATTTGGATTGGTGTATACAGTGTATGCAACAGCAGCTGATCCAAAGAAAGGGTCATTAGG
AACAATAGCACCAATAGCAATTGGTTTAAGTGTTGGTGCAAACATATTAGCTGCAGGTCCATTC
TCAGGTGGTTCAATGAATCCAGCACGTTCATTTGGACCAGCAGTTGTTAGTGGTGATTTTCATG
ACAATTGGATATATTGGGTTGGTCCTCTTATTGGTGGTGGTTTGGCTGGTCTTATCTATACTCA
TCTCTTCATTCCATCAAAACATCAACAACCTATGCCCAAGTGA

>Ca_02797 (CaTIP2-1)

MARISFGDFEDSFSSSSIRAYIAEFISTLLFVFAGVGSTRAFDKLTSDAALDPAGLLSIAVCHG
FALFVAVSVGANISGGHVNPAVTFGMALGGQITILTSLFYCIAQFLGSIAACLLLKFVTGSLTN
IPIHSIGAGVGVGEGVVTEIVITFGLVYTVYATAADPKKGSLGTIAPIAIGLSVGANILAAGPF
SGGSMNPARSFGPAVVSGDFHDNWIYWVGPLIGGGLAGLIYTHLFIPSKHQQPMPK

>Ca_24137 (CaTIP2-2)

ATGGCTGGCATAGCATTTGGACGTTTTGATGATTCCTTCAGTTTTGGCTCAATTAAGGCATATA
TTGCTGAATTCATCTCAACTTTGATCTTTGTTTTTGCTGGTGTTGGTTCAGCCATAGCCTATGG
TAAGTTGACATCAGATGCAGCTCTGGATCCAGCTGGATTGCTAGCAGTTGCAATTTGCCATGGT
TTTGCACTGTTTGTTGCTGTTTCAGTTGGTGCTAACATTTCTGGTGGACATGTCAATCCTGCTG
TCACATTTGGATTGGCTCTTGGTGGACACATCACAATCCTCACTGGTATCTTCTACTGGATTGT
ACAGCTTCTTGGCTCCATAGTTGCATGTTTTCTTCTTCAATTTGTCACAGGAGGATTGGAAACT
CCAACACACAGTGTGGCAGCTGAAGTAGGACCTATTGGAGGAATTGTGACTGAAATAATCATAA
CATTTGGTTTAGTGTACACAGTATATGCCACAGCAGCTGATCCAAAAAAAGGTTCATTGGGAAC
AATTGCACCCATTGCTATTGGATTCATTGTTGGAGCAAACATATTAGCAGCAGGCCCATTCTCT
GGTGGATCAATGAACCCAGCTAGATCTTTTGGGCCTGCTGTTGTTAGTGGCAATTTCCATGATA
ATTGGGTCTATTGGGTTGGGCCTCTTGTTGGTGGTGGTTTGGCTGGGCTTATTTATGACAATGT
GTTCCTGCGTTCAGAACATGCTCCACTTGCTAGTGATTATTAA

>Ca_24137 (CaTIP2-2)

MAGIAFGRFDDSFSFGSIKAYIAEFISTLIFVFAGVGSAIAYGKLTSDAALDPAGLLAVAICHG
FALFVAVSVGANISGGHVNPAVTFGLALGGHITILTGIFYWIVQLLGSIVACFLLQFVTGGLET
PTHSVAAEVGPIGGIVTEIIITFGLVYTVYATAADPKKGSLGTIAPIAIGFIVGANILAAGPFS
GGSMNPARSFGPAVVSGNFHDNWVYWVGPLVGGGLAGLIYDNVFLRSEHAPLASDY

>Ca_02338 (CaTIP2-3)

ATGGTGAAGATAGCTTTTGGTACATTTGATGACTCTTTTAGTGTTGCCTCTCTTAAGGCTTATC
TATCAGAGTTCATTGCCACTCTGATTTTTGTGTTTGCAGGAGTTGGATCAGCCATTGCTTACAA
TGATATTACATCAGATGCAGCCTTGGATCCAGCTGGTCTAGTGGCAGTAGCTATTGCTCATGCA
TTTGCACTATTTGTGGGAGTGTCAATTGCAGCCAACATTTCAGGTGGACATTTGAATCCAGCTG
TGACTTTTGGATTAGCCATTGGAGGCAACATCACAATCATAACTGGTCTCTTCTATTGGATTGC
TCAATTGCTTGGCTCCATTGTTGCAAGTCTCCTCCTCAATTATGTCACCTCTAAGAGTGTTCCA
ACCCATGGAGTGGCTGCTGGATTGAGCCCTATTGCAGGTTTAGTGTTTGAGATTATTGTTACCT
TTGGATTGGTTTACACTGTTTATGCCACAGCAGCTGACCCCAAAAAGGGCTCATTGGGTACCAT
CGCACCTATTGCTATTGGGTTCATAGTTGGTGCCAACATCTTAGTTGCCGGTCCATTCAGTGGC
GGTTCAATGAACCCGGCTCGGTCATTCGGACCAGCTGTGGTTAGTGGAAACTTTGCTGATAACT

GGATCTACTGGGTTGGCCCATTGATAGGAGGAGGTTTGGCTGGGTTGATTTATGGTGATATCTT
CATTGGTTCCTATGCCCCAGCCCCAGCAAGTGAAACATACCCTTGA
>Ca_02338 (CaTIP2-3)
MVKIAFGTFDDSFSVASLKAYLSEFIATLIFVFAGVGSAIAYNDITSDAALDPAGLVAVAIAHA
FALFVGVSIAANISGGHLNPAVTFGLAIGGNITIITGLFYWIAQLLGSIVASLLLNYVTSKSVP
THGVAAGLSPIAGLVFEIIVTFGLVYTVYATAADPKKGSLGTIAPIAIGFIVGANILVAGPFSG
GSMNPARSFGPAVVSGNFADNWIYWVGPLIGGGLAGLIYGDIFIGSYAPAPASETYP

>Ca_03854 (CaTIP3-1)
ATGGCAACACGAAGATATACATTCGGAAGTTTGGAGGAGGCAAACCATGGAGATTCCATAAGAG
CAACATTAGCTGAATTGATATCAACTTGCATATTTGTTTTTGCTGGAGAAGGCTCTGCTCTTGC
TCTAACCAAAATATACAAAGATGCTGGATCATCAGCTGGGGAGTTAGTTGTACTTGCATTGGCT
CATTCTTTTTCACTTTTTGCTGCTATTTCATCCACAGCTCATGTTTCTGGTGGACATGTTAATC
CTGCTGTTACTTTTGGTGCTCTTCTTGGTGGAAGGATATCTGTTATTAGAGCACTATACTATTG
GATTGCTCAACTTCTTGGTTCTGTAGTTGCTGCTCTTTTATTGAGACTTGTCACTAATAACATG
AGACCGCAAGCATTCAGTGTGGCAGTTGGCGTTGGTGCAGGGCAGAGTCTCATACTTGAGATTG
CGATGACATTTGGTTTGATGTACACTGTTTATGCCACAGCCATTGATCCCAAAAGAGGAACCGT
TGGAACTATTGCTCCCTTAGCAATTGGACTTGTTGTTGGGGCAAACATTCTAGCTGGTGGCCCG
TTTGATGGAGCATGCATGAACCCTGCTCGGGCTTTTGGGCCTGCTTTAGTGGGCTGGAGATGGC
ATTTCCATTGGATCTATTGGGTCGGTCCATTACTTGGGGCTGCAATTGCAGCACTTTTATATGA
ATATGTTATACTTCCAACTGTGGCTCCTAATCCTAATCCACATCCACATCCACATCATCAACCT
TTGGCTCCTGAAGATTACTAG
>Ca_03854 (CaTIP3-1)
MATRRYTFGSLEEANHGDSIRATLAELISTCIFVFAGEGSALALTKIYKDAGSSAGELVVLALA
HSFSLFAAISSTAHVSGGHVNPAVTFGALLGGRISVIRALYYWIAQLLGSVVAALLLRLVTNNM
RPQAFSVAVGVGAGQSLILEIAMTFGLMYTVYATAIDPKRGTVGTIAPLAIGLVVGANILAGGP
FDGACMNPARAFGPALVGWRWHFHWIYWVGPLLGAAIAALLYEYVILPTVAPNPNPHPHPHHQP
LAPEDY

>Ca_19737 (CaTIP3-2)
ATGGCAACTCGTAGGTATGCTTTTGGAAGAGCTGATGAGGCTACACATCCTGATTCAATTAGAG
CAACTATAGCTGAATTTGCATCCACTTTCATCTTTGTCTTTGCTGGAGAAGGATCTGGCCTTGC
TTTGGTTAAGATTTACCAAGACTCAGCATTCTCAGCTGGTGAACTGTTGGCAACAGCACTTGCA
CATGCTTTTGCACTATTTGCTGCTGTGTCTGCTAGTATGCATGTATCTGGTGGTCATATCAACC
CAGCAGTTACATTTGGTGCTCTCATTGGTGGCAGAATCTCTGTTCTTAGAGCTGTCTACTACTG
GATTGCTCAACTTCTTGGTGCTGTTGTTGCTGCTCTCTTGCTTAGGCTTGTCACTAATAACATG
AGACCAGCAGGGTTTCATGTAGGAGATGGTATTGGTTCAGGACATGCACTTATACTTGAGATAA
TAATGACATTTGGGCTAATGTACACAGTATATGCAACTGCAATTGATCCTAAAAGAGGTACCAT
TGGTGCTATTGCACCTTTAGCAATTGGACTCATTGTTGGTGCAAATATCCTTGTTGGTGGGGCA
TTTGATGGAGCATGTATGAACCCTGCTCTTGCTTTTGGGCCTTCTTTGGTTGGCTGGAGATGGC
ACCAACATTGGATCTTTTGGCTTGGTCCATTCATTGGAGCAGCATTGGCAGCAATCATATATGA
ATATGTTATTATCCCAACTGAGCCACCTCATGCACACCAGCCTCTGGCTCCTGAAGATTACTAG
>Ca_19737 (CaTIP3-2)
MATRRYAFGRADEATHPDSIRATIAEFASTFIFVFAGEGSGLALVKIYQDSAFSAGELLATALA
HAFALFAAVSASMHVSGGHINPAVTFGALIGGRISVLRAVYYWIAQLLGAVVAALLLRLVTNNM

RPAGFHVGDGIGSGHALILEIIMTFGLMYTVYATAIDPKRGTIGAIAPLAIGLIVGANILVGGA
FDGACMNPALAFGPSLVGWRWHQHWIFWLGPFIGAALAAIIYEYVIIPTEPPHAHQPLAPEDY

>Ca_14915 (CaTIP4-1)
ATGGTCAAAATTTCATTAGGAAGAATCAAAGAGGCCACTCAGCCAGATTGCATTCAAGCTCTAA
TTGTTGAATTCATTACCACTTTTCTATTTATCTTTGCTGGTGTTGCTTCTGCAATCACTGCCGA
AAAGCTAAGTGGAGATGCATTAGTGGGATTGTTTTTTGTGGCTATAGCACAGACTCTTGTGGTG
GCTGTAATGATCGGCGCCGGTGGCCACTTTAACCCCGCCGTGACTCTAGGTCTCCTTGTCGGTG
GCCACATCACTATGGTCCGTTCCATTTTATATTGGATTGATCAATTAATAGCATCAGCATCAGC
TTGTTATCTTCTTCATTATCTCTCTGGTGGATTGACAACTCCAGCTCAAACACTTGCAAGTGGA
GTAGGGTACACACAAGGAGTAGTTTGTGGGATTGTGTTGACATTCTCTTTGTTATTCACTGTTT
ATGCAACCATGGTTGATCCAAAGAAGGGAGTACGTCATGGGCTTGGCCCAACTTTAGTTGGGTT
TGTGGTAGGGGCTAACATTCTAGCTGGGGCTTTCTCTGGTGCTTCAATAAACCCAGCAAGATCT
TTTGGGCCTGCTTTGGTTAGTGGAAATTGGACTAATCATTGGGTTTATTGGGTTGGGCCACTTA
TTGGTGGTGGGTTTGCTGGTTTTATTTATGAAAATTTCTTCATTTAG
>Ca_14915 (CaTIP4-1)
MVKISLGRIKEATQPDCIQALIVEFITTFLFIFAGVASAITAEKLSGDALVGLFFVAIAQTLVV
AVMIGAGGHFNPAVTLGLLVGGHITMVRSILYWIDQLIASASACYLLHYLSGGLTTPAQTLASG
VGYTQGVVCGIVLTFSLLFTVYATMVDPKKGVRHGLGPTLVGFVVGANILAGAFSGASINPARS
FGPALVSGNWTNHWVYWVGPLIGGGFAGFIYENFFI

>Ca_14916 (CaTIP4-2)
ATGGCCAAAATCGCGTTAGGAACAACAAGAGAGGCCACTCAACCAGATTGCATTCAAGCTCTAA
TCGTTGAATTCATTGCCACTTTTCTCTTTGTTTTTGCTGGTGTTGGTTCTGCCATGACTGCTGA
TAAGCTAAGTGGAGATGCATTAGTGGGATTGTTTTTTGTGGCTATAGCACATGCTCTTGTGGTG
GCTGTAATGATCTCCGCCGCTCACATTTCCGGTGGCCATTTGAATCCGGCGGTGACGCTAGGTC
TCCTTGCCGGCGGCCACATCACTGTGTTCCGTTCAATTCTATATTGGATTGATCAATTAATAGC
ATCTGCTGCAGCTTCTTATCTTCTGTATTATCTCTCTGGTGGATTGACAACTCCAGCTCATACA
CTTGCAAGTGGAATAGGGTACACACAAGGTGTAGTTTGGGAGATTGTGTTGACATTCTCTTTGT
TATTCACTGTTTATGCAACCATGGTTGATCCAAAGAAGGGAGCACTTAATGGGCTTGGCCCAAC
TTTAGTTGGGTTTGTGGTAGGGGCCAATATTCTAGCTGGTGGGGCTTTCTCTGCTGCTTCAATG
AACCCAGCAAGATCTTTTGGGCCTGCTTTGGTTAGTGGAAATTGGACTGATCATTGGGTTTATT
GGGTTGGGCCACTTATTGGTGGTGGGCTTGCTGGTTTCATTTATGAGAATTTCTTCATTAATAG
AGATCATGTCCCTCTAGTTGTAGATGAAGAAAGTTACTAA
>Ca_14916 (CaTIP4-2)
MAKIALGTTREATQPDCIQALIVEFIATFLFVFAGVGSAMTADKLSGDALVGLFFVAIAHALVV
AVMISAAHISGGHLNPAVTLGLLAGGHITVFRSILYWIDQLIASAAASYLLYYLSGGLTTPAHT
LASGIGYTQGVVWEIVLTFSLLFTVYATMVDPKKGALNGLGPTLVGFVVGANILAGGAFSAASM
NPARSFGPALVSGNWTDHWVYWVGPLIGGGLAGFIYENFFINRDHVPLVVDEESY

>Ca_15805 (CaTIP5-1)
ATGGCTTCACGTTTTCATGAATCCTTCACACGAGATGCACTTCGCTCTTATTTCGCAGAGTTTA
TTTCCACTTTCTTCTATGTCCTTATTGTTATTGCCTCTGGAATGTCTTCAAGGAAGTTGATGCC
TGATGCTTCAGTGAACCCAACAAGTTTGGTTGTTGGAGCAATTGCAAATGCTTTTGCTCTGTCC

TCAGTTTTGTACATTGCATGGGACATTTCAGGTGGACATGTTAATCCTGCTGTCACATTTGCAA
TGGCTGTTGGAGGACATATTAGTGTCCCAACTGCTCTCTTCTATTGGATTGCCCAACTTATTGC
CTCTGTTATGGCTTGCTTTTTCCTCAGACTCTTTCTTGTTGGAATGCATGTGCCAACTTATTCA
ATTGCAGAAGAGATGACAGGTTTTGGAGCATCAATATTAGAGGGTATATTAACATTTGTTTTGG
TGTACACAATATATGCTGCAAGGGACACTAGGCGTGGTGGTCAACAATTGAGTTCAACACTTGT
AATTGGTTTAATATCAGGAGCAAGTGTTTTAGCAGCAGGTCCATTCTCTGGTGGATCAATCAAC
CCTGCTTGTGCTTTTGGCTCTGCTTCCATTGCTGGAACTTTTAGGAATCAAGCTGTTTATTGGG
TTGGTCCTTTGATTGGTGCTACTGTTGCTGGTCTTCTTTATGATAATGTGTTGTTCCCTTCTCA
GAGTTTAGATTCAATTAGAGGGGTTTCTGAAGTAAATGTTAGGGTGTAA
>Ca_15805 (CaTIP5-1)
MASRFHESFTRDALRSYFAEFISTFFYVLIVIASGMSSRKLMPDASVNPTSLVVGAIANAFALS
SVLYIAWDISGGHVNPAVTFAMAVGGHISVPTALFYWIAQLIASVMACFFLRLFLVGMHVPTYS
IAEEMTGFGASILEGILTFVLVYTIYAARDTRRGGQQLSSTLVIGLISGASVLAAGPFSGGSIN
PACAFGSASIAGTFRNQAVYWVGPLIGATVAGLLYDNVLFPSQSLDSIRGVSEVNVRV
