# Supplementary material for: Genome-Wide Analysis of the Aquaporin Gene Family in Chickpea (Cicer arietinum L.)
Source: Front Plant Sci. 2016 Nov 29;7:1802. doi: 10.3389/fpls.2016.01802 (PMC5126082; doi:10.3389/fpls.2016.01802)
Supplement: Supplementary File S2 — Comparison of number of different AQP subfamily members in chickpea, Arabidopsis, Medicago, common bean, and soybean. [file DataSheet2.PDF]

**Supplementary File S2. Comparison of number of different AQP subfamily members in chickpea, Arabidopsis, Medicago, common bean, and soybean**

| **Subfamily** | **Chickpea** | **Arabidopsis** | **Medicago** | **Common bean** | **Soybean** |
|---|---|---|---|---|---|
| PIPs | 9 | 13 | 8 | 12 | 22 |
| TIPs | 12 | 10 | 8 | 13 | 23 |
| NIPs | 16 | 9 | 16 | 10 | 17 |
| SIPs | 3 | 3 | 3 | 4 | 8 |
| XIPs | 0 | 0 | 0 | 2 | 2 |
| Total AQP | 40 | 35 | 35 | 41 | 72 |
