# Supplementary material for: Genome-Wide Analysis of the Aquaporin Gene Family in Chickpea (Cicer arietinum L.)
Source: Front Plant Sci. 2016 Nov 29;7:1802. doi: 10.3389/fpls.2016.01802 (PMC5126082; doi:10.3389/fpls.2016.01802)
Supplement: Supplementary File S3 — Multiple sequence alignment of deduced amino acid sequences of 40 CaAQP genes. Sequence alignment was performed using CLUSATLW. Transmembrane helices (TM1–TM6) and two NPAs (LB and LE) are highlighted in gray color, P1–P5 residues highlighted in blue color, and ar/R selectivity filter residues highlighted in green color. The NPA motifs are shown in red color. [file DataSheet3.PDF]

```
CaPIP1-3     ------------MEAKEQDVSLG-----------------------ANKYPERQPIGIAA
CaPIP1-4     ------------MEAKEQDVSLG-----------------------ANKFPERQPLGIAA
CaPIP1-1     -------------MEREEDVKIG-----------------------ANKFSEKSALGIGA
CaPIP1-2     -------------MEKEEDVKVG-----------------------ASKFSERQALGTAA
CaPIP2-5     -----------MSKEVSEEGHLQ-----------------------THHHGG--------
CaPIP2-2     ---------MAKDIQTEPQTALP-----------------------N-------------
CaPIP2-1     ---------MAKNVEVAERGSFS-----------------------N-------------
CaPIP2-3     -----------MAKDVEVQEHGE-----------------------FSA-----------
CaPIP2-4     -----------MGKDVEVQEQGG-----------------------EYSA----------
CaTIP5-1     --------------------MAS-----------------------R-------------
CaTIP4-1     ----------------MVKISLG-----------------------R-------------
CaTIP4-2     ----------------MAKIALG-----------------------T-------------
CaTIP3-1     --------------MATRRYTFG-----------------------S-------------
CaTIP3-2     --------------MATRRYAFG-----------------------R-------------
CaTIP2-3     ----------------MVKIAFG-----------------------T-------------
CaTIP2-1     ----------------MARISFG-----------------------D-------------
CaTIP2-2     ----------------MAGIAFG-----------------------R-------------
CaTIP1-1     --------------MPIRNIAIG-----------------------N-------------
CaTIP1-2     --------------MPISRIAIG-----------------------S-------------
CaTIP1-3     --------------MAIYRIAIG-----------------------S-------------
CaTIP1-4     --------------MAIYRIAIG-----------------------S-------------
CaNIP4-1     MFEKQSSPELASNYASSSGLSGD-----------------------DKEIGYRASTLK--
CaNIP2-1     -----------MDRRTHSLVNAT-----------------------N-------------
CaNIP3-4     MPGLETGTPTAASTPATPDTPGG-----------------------PLFSSVRVDSLDQR
CaNIP3-1     -----------MEHSNNEEIPSTP--ATPGTPGVPLFGGFKSERNGNGSNKNKKSLLKNC
CaNIP6-1     -----------MDNEEIPSIPGTPGNATPGTPGAPLFGGLKPEKNGNRSVGRNKSLLKNM
CaNIP3-2     -----------------MKYSYK-------------------------------------
CaNIP3-3     -----------MNCFTIEEWNLE-------------------------------------
CaNIP1-9     -----------MDDNSESNEIHH-----------------------EVV-----------
CaNIP1-6     -----------MGDILDSNDVVL-----------------------K-------------
CaNIP1-8     ----------MGDISATNDVVLN-------------------------------------
CaNIP1-5     -----------MANNNSARIETL-----------------------DVV-----------
CaNIP1-7     -----------MADHSESNGNRE-----------------------MV------------
CaNIP1-3     -----------MDDNSASNGTIN-----------------------EVV-----------
CaNIP1-4     -----------MDDNSASNGTIN-----------------------EVV-----------
CaNIP1-2     -----------MANKREGNIQLD-----------------------EETSS---------
CaNIP1-1     -----------MEEGGFKELTGN-----------------------EGGF----------
CaSIP2-1     ------------------------------------------------------------
CaSIP1-1     ------------------------------------------------------------
CaSIP1-2     ------------------------------------------------------------

                                                        TM1
CaPIP1-3     QSQDDG---KDYKEPPPAPLFEPSE-LTSWSFYRAGIAEFVATFLFLYITILTVMGVNRS
CaPIP1-4     QSQDEP---KDYQEPPPAPLFEPSE-LTSWSFYRAGIAEFIATFLFLYITVLTVMGVVRE
CaPIP1-1     KSDS-----KDYKEAPAAPLFEPGE-LKSWSFYRAGIAEFVATFLFLYISVLTVMGVNRS
CaPIP1-2     QND------KDYKEVPAAPLFEPGE-LKSWSFYRAGIAEFIATFLFLYITILTVMGVNRS
CaPIP2-5     ---------KDYVDPPPAPLLDFAE-IKLWSFYRALIAEFIATLLFLYVTVATVIGHKKQ
CaPIP2-2     ---------KDYQDPPPAPLFDTSE-LSQWSFYRALIAEFVATLLFLYVTVATVIGYNSQ
CaPIP2-1     ---------KDYHDPPPAPFIDAAE-LTKWSFYRALIAEFIATLLFLYVTVLTVIGYSIQ
CaPIP2-3     ---------KDYQDPPPAPLIDFDE-LTKWSFYRALIAEFVATLLFLYVTILTIIGYSHQ
CaPIP2-4     ---------KDYQDPPPAPLFDPAE-LTKWSLYRAVIAEFIATLLFLYITVLTIIGYSRQ
CaTIP5-1     ----------------------FHE-SFTRDALRSYFAEFISTFFYVLIVIASGMSSRKL
CaTIP4-1     ----------------------IKE-ATQPDCIQALIVEFITTFLFIFAGVASAITAEKL
CaTIP4-2     ----------------------TRE-ATQPDCIQALIVEFIATFLFVFAGVGSAMTADKL
```

```
                                                      TM1
CaTIP3-1   -----------------------LEE-ANHGDSIRATLAELISTCIFVFAGEGSALALTKI
CaTIP3-2   -----------------------ADE-ATHPDSIRATIAEFASTFIFVFAGEGSGLALVKI
CaTIP2-3   -----------------------FDD-SFSVASLKAYLSEFIATLIFVFAGVGSAIAYNDI
CaTIP2-1   -----------------------FED-SFSSSSIRAYIAEFISTLLFVFAGVGSTRAFDKL
CaTIP2-2   -----------------------FDD-SFSFGSIKAYIAEFISTLIFVFAGVGSAIAYGKL
CaTIP1-1   -----------------------PQE-ATHPDTLKAGLAEFISTFIFVFAGSGSSIAYNKL
CaTIP1-2   -----------------------PSE-FGKADALKAALAEFISMLIFVFAGEGSGMAYNKL
CaTIP1-3   -----------------------PRE-ASNPAAIRAAFAEFFSMLIFVFAGQGSGMAYSKL
CaTIP1-4   -----------------------PGE-AGQPDAIRAAFAEFFSMLIFVFAGEGSGMAYNKL
CaNIP4-1   ---------HGYLLANNSSLHFLPN-KIDLNFARMVMAEVVGTFILMFCVCGIIASTQHQ
CaNIP2-1   --------------DFQNHITQKQS-LYPSGFPRKVLAEVIGTYLLVFVGSGSAAMNAID
CaNIP3-4   ESFGMGRCNNCFPGKSNGGCINIADFSASVSLTQKIGAEFVGTFILIYAATAGPIVNNKY
CaNIP3-1   KCFSVQDWTIEDGALPTVSCSL-MP-PPPVPLAKKIGAEFIGTFILIFAGTATAIVNQKT
CaNIP6-1   NCFAVEEWNLEDGSLPRVSCALPLP-PAPIPLAKKVGAEFIGTFILMFAGIGTAIENQKV
CaNIP3-2   --------------------------FRSIPLAKKVAAEFIGTFILMFAGMGSAIENEKV
CaNIP3-3   ------------DGYLPRVSCALPLP-HAPIPLAKKVAAEFIGTFILMFAGMGSAIENEKV
CaNIP1-9   ---------LNVNGDASKNCDESGF-KDSVPLLKKLVAEVVGTYLMIFAGCAAVLVNLNN
CaNIP1-6   --------------------VDDSII-EDSVPLLQKLVAEVVGTFMLIFVGCGVVVTNLNN
CaNIP1-8   ------------------VDANDSII-EDSVPLLQKLVAEVVGTFMLIFVGCGVVVTNLNN
CaNIP1-5   ---------LDVNKDSSRTCEGSDS-YVSVPFLQKLIAEMVGTYFLIFAGCASVVVNNNK
CaNIP1-7   ---------LNVNGDASNICDNSSI-EERVPLLKKLVAEVVGTYLLIFAGCGAVVVNLDK
CaNIP1-3   ---------LNVNKDDLKITENSTA-PATASFLQKLVAEVVGTYFLIFAGCASVVVNKNN
CaNIP1-4   ---------LNVNKDDLKITENSTA-PATASFLQKLVAEVVGTYFLIFAGCASVVVNKNN
CaNIP1-2   ---------SVDEGNPSIMQFCCSS-NHTITLIQKVIAEIIGTYFLVFAGCGAVVVNKIY
CaNIP1-1   -----------------------CGS-PEVVQVIQKVIAEVIGSYFLIFAGCCSVVLNKVE
CaSIP2-1   --------------------------MERSKLI--IVSDFVMSFMWVCSGVLVRLFVFKV
CaSIP1-1   ----------------------------MVSAIKSAIGDAVLTFMWVFCSSMLGIVTNAI
CaSIP1-2   ----------------------------MVNAIKAAIGDAVLTFTWLFISSTLGLVTNEI
                                                       :        :

                                     TM2                  LB
CaPIP1-3   DSK-------CKSVGIQG--IAWSFGGMIFALVYCTAGISGG-HINPAVTFGLFLARKLS
CaPIP1-4   SSK-------CKTVGIQG--IAWAFGGMIFALVYCTAGISGG-HINPAVTFGLFLARKLS
CaPIP1-1   TSK-------CASVGIQG--IAWAFGGMIFALVYCTAGISGG-HINPAVTFGLFLARKLS
CaPIP1-2   TSK-------CSSVGIQG--IAWSFGGMIFALVYCTAGISGG-HINPAVTFGLFLARKLS
CaPIP2-5   TGP-------CDGVGLLG--IAWSFGGMIFVLVYCTAGISGG-HINPAVTFGLFLARKVS
CaPIP2-2   TDP-AHNGTACDGVGILG--IAWAFGGMIFVLVYCTAGISGG-HINPAVTFGLFLARKVS
CaPIP2-1   TDI-KAGGDVCGGVGILG--IAWAFGGMIFVLVYCTAGISGG-HINPAVTFGLFLARKVS
CaPIP2-3   SDP-KAGGTDCDGVGILG--IAWAFGGMIFILVYCTAGISGG-HINPAVTFGLFVGRKVS
CaPIP2-4   TDTTITGNTECDGVGVLG--IAWAFGGMIFVLVYCTAGISGG-HINPAVTFGLFLGRKVS
CaTIP5-1   MPD-----ASVNPTSLVV--GAIANAFALSSVLYIAWDISGG-HVNPAVTFAMAVGGHIS
CaTIP4-1   SGD---------ALVGLFF--VAIAQTLVVAVMIGAGG------HFNPAVTLGLLVGGHIT
CaTIP4-2   SGD---------ALVGLFF--VAIAHALVVAVMISAA-HISGG-HLNPAVTLGLLAGGHIT
CaTIP3-1   YKD-----AGSSAGELVV--LALAHSFSLFAAISSTAHVSGG-HVNPAVTFGALLGGRIS
CaTIP3-2   YQD-----SAFSAGELLA--TALAHAFALFAAVSASMHVSGG-HINPAVTFGALIGGRIS
CaTIP2-3   TSD-----AALDPAGLVA--VAIAHAFALFVGVSIAANISGG-HLNPAVTFGLAIGGNIT
CaTIP2-1   TSD-----AALDPAGLLS--IAVCHGFALFVAVSVGANISGG-HVNPAVTFGMALGGQIT
CaTIP2-2   TSD-----AALDPAGLLA--VAICHGFALFVAVSVGANISGG-HVNPAVTFGLALGGHIT
CaTIP1-1   TND-----GAATPSGLIS--AAIAHAFALFVAVSVGANISGG-HVNPAVTFGAFVGGNIT
CaTIP1-2   TNN-----GAATPAGLVA--ASLSHAFALFVAVSVGANISGG-HVNPAVTFGAFIGGHIT
CaTIP1-3   TNN-----GAATPEGLIV--ASLSHAFGLFVAVSVGANISGG-HVNPAVTFGAFIGGNIT
CaTIP1-4   TNN-----GPATPAGLIA--ASLSHAFGLFVAVSVGANASGG-HVNPAVTFGAFMGGNIT
CaNIP4-1   NG----------AVGLLE--YAATAGLTVVVIIFSIGPISCA-HVNPAVTIAFATIGQFP
CaNIP2-1   ENK----------VSKLG--ASMAGGFIVTVMIYAIGHISGA-HMNPAVSLAFATVSHFP
CaNIP3-4   NG----------AESLMG--NAACAGLTVMFIILSIGHISGA-HLNPSLTIAFAAFRHFP
CaNIP3-1   KGS-----------ETLIG--CAASSGLAVMIVILSTGHISGA-HLNPAVTISFAALKHFP
CaNIP6-1   ENS-----------ETLIG--CAGASGLAVMIIILSTGHISGA-HLNPAVTISFAALKHFP
CaNIP3-2   ENS-----------ETLIG--CAGASGLAVMIIILSTGHISGA-HLNPA----------HFP
```

```
                                                 TM2                      LB
CaNIP3-3     ENS----------ETLIG--CVGASGLAVMIIILSTGYISGA-HLNPAVTISFVALKHFP
CaNIP1-9     DH----------VVTLPG--IAVTWGFTVMVLIYSLGHISGA-HFNPAVTIAHASTKTFP
CaNIP1-6     DN----------VVTLPG--VAIVWGLAVMVLAYSLGHVSGA-HFNPAVTIAQASTKRFP
CaNIP1-8     DNV----------VTLPG--VAIVWGLAVMVLAYSLGHVSGA-HFNPAVTIAQASTKRFP
CaNIP1-5     DN----------VVTLPG--IAIVWGLTLMVLVYSLGHISGA-HFNPAVTLAFASTRRFP
CaNIP1-7     DK----------VITHPG--ISIVWGLTVMVLVYSIGHISGA-HFNPAVTIAHASTKRFP
CaNIP1-3     EN----------VVTLPG--ISIVWGLAVMVLVYSLGHISGA-HFNPAVTIAFATTRRFP
CaNIP1-4     EN----------VVTLPG--ISIVWGLAVMVLVYSLGHISGA-HFNPAVTIAFATTRRFP
CaNIP1-2     GS-----------VTFPG--ICITWGLIVMVMCYSLGHISGG-HFNPAVTITWTLFRRIS
CaNIP1-1     GSK--------GTITFPG--ICIVWGVSVMILVYALGHISGA-HFNPAV-----------
CaSIP2-1     LAF---------SHTHIAEIVKIVFSIANMFLFAFLAKVSRGGAYNPLTVLADAFSGDFH
CaSIP1-1     TKSLDLQDVSYNGFPYPSFIVITTLVFLLVFLFTLIGSAMGGASFNPTGTASFYAVGLGS
CaSIP1-2     MKFFDLQFVTYNGLNYPFIIITILLIFITIITFTTIGNALGGASFNPTGNASLYAAGLGS
                                                          **

                          TM3                                       TM4
CaPIP1-3     --LTRAVFYIVMQVLGAICGAGVVKGFEGKTLYGKFHGGANFVAPGYTKGDGLGAEIIGT
CaPIP1-4     --LTRALFYMVMQVLGAICGAGVVKGFEGKTRFGDLKGGANFVNPGYTKGDGLGAEIVGT
CaPIP1-1     --LTRAVFYIIMQCLGAICGAGVVKGFEGNARYEMYKGGANVVNPGYTKGDGLGAEIVGT
CaPIP1-2     --LTRAIFYIVMQCLGAICGAGVVKGFEGNARYELFKGGANFVNAGYTKGDGLGAEIVGT
CaPIP2-5     --LIRAVLYMVAQCLGAICGVGLVKALM-KQPYNNLGGGANSVASGYSKGSALGAEMIGT
CaPIP2-2     --LIRAILYMVVQCLGAICGVGLVKGFQ-KSYYNRYKGGANMLSNGYSKGTGLGAEIIGT
CaPIP2-1     --LIRAIMYIVAQCLGAICGVGLVKAFQ-SAYFDRYGGGANFLHDGYSTGVGLGAEIVGT
CaPIP2-3     --LLRAVFYMAAQCAGAISGTGLAKGFQ-KAYFDRYGGGANFVHDGYNKGTALGAEIIGT
CaPIP2-4     --LIRAVLYIIAQCLGAICGAGLAKGFQ-KSYYNRYHGGVNLVSDGYSKGTALGAEIIGT
CaTIP5-1     --VPTALFYWIAQLIASVMACFFLRLFL-----VGMHVPTYSIAEEMTGFGASILEGILT
CaTIP4-1     --MVRSILYWIDQLIASASACYLLHYLS-----GGLTTPAQTLASGVGYTQGVVCGIVLT
CaTIP4-2     --VFRSILYWIDQLIASAAASYLLYYLS-----GGLTTPAHTLASGIGYTQGVVWEIVLT
CaTIP3-1     --VIRALYYWIAQLLGSVVAALLLRLVT-----NNMRPQAFSVAVGVGAGQSLILEIAMT
CaTIP3-2     --VLRAVYYWIAQLLGAVVAALLLRLVT-----NNMRPAGFHVGDGIGSGHALILEIIMT
CaTIP2-3     --IITGLFYWIAQLLGSIVASLLLNYVT------SKSVPTHGVAAGLSPIAGLVFEIIVT
CaTIP2-1     --ILTSLFYCIAQFLGSIAACLLLKFVT----GSLTNIPIHSIGAGVGVGEGVVTEIVIT
CaTIP2-2     --ILTGIFYWIVQLLGSIVACFLLQFVT-----GGLETPTHSVAAEVGPIGGIVTEIIIT
CaTIP1-1     --LLRGIVYIIAQLLGSIVASLLLVFAT------GLSVPAFSLSAGVGVGPALVLEIVLT
CaTIP1-2     --LIRGLLYWIAQLLGSVVACLLLKIAT-----GGLETSAFSLSSGVGATNALVFEIVMT
CaTIP1-3     --FLRSILYWIAQLLGSVAACIILNSCT-----GGMETSAFSLSSGVSVWNALVFEIVMT
CaTIP1-4     --LLRSILYWIAQLLGSVVACILLKSAT-----GGMETSAFAISSDISVWNALVFEIVMT
CaNIP4-1     --WFKVPVYIIAQTIGSLMATYIGSLVY------GIKSDVMMTQPLQGCNSAFWVEVIAT
CaNIP2-1     --WKQVPFYIAAQLTGAISASYTLKVLL----EPSKQLGA--TSPSGSNIQALIIEIVTT
CaNIP3-4     --WAHVPAYIAAQVSASICACYALKVVY----HPFLTGGV--TVPTVSIGQAFATEFIIT
CaNIP3-1     --WKHVPMYIGAQVLASICAAFALKGVF----HPFMSGGV--TVPSGGYGQAFALEFIIS
CaNIP6-1     --WKN-------------------------------------------------------
CaNIP3-2     --WKNVPVYIGAQILASICAAFSLKVIF----HPFMNGGV--TVPSVAIGEAFALKFIIG
CaNIP3-3     --WKNVPVYIGAQILASICAAFSLKVIF----HPFMNGGV--TVPSVAIGEAFALEFIIG
CaNIP1-9     --LKQVPAYIIAQVLGSILASGTLKLIF-----NGKDGHFIGTLPTGSNLQAFVIEFICT
CaNIP1-6     --VQQIPTYIIAQLFGSILASVVLKVIF-----SDKENRFVGTLPAGSNLQAFVVEFLIT
CaNIP1-8     --VQQIPTYIIAQLFGSILASVVLKVIF-----SDKENRFVGTLPAGSNLQAFVVEFLIT
CaNIP1-5     --LVQVPAYLSAQVLGATLGSGTLKLIF-----SGSHDQFTGTLPTGSNFQAFVLEFIIT
CaNIP1-7     --LKQVPAYVIAQVLGSTLASGTLRLIF-----NGKENHFSGTLPAGSNLQCFVVEFIIT
CaNIP1-3     --LKQVPGYVVAQVLGSTLASGTLRLLF-----SGKDNQFAGTLPDGSNLQAFVVEFIIT
CaNIP1-4     --LKQVPGYVVAQVLGSTLASGTLRLLF-----SGKDNQFAGTLPDGSNLQAFVVEFIIT
CaNIP1-2     --IKEAPLYIFAQLLGSTLASGTLSLMF-----DITPKTYFGTVPSGSNGQSLVVEIIIS
CaNIP1-1     ------PLYLIAQVLGSILASGTLYLLF----DDLNESTYFGTVPAGSDVQSLVFEIITS
CaSIP2-1     NFIFCVGSRIPAQVVGSIVG---VKFLI-----DTIPEVGRGPRLNVDIHRGALTEGLLT
CaSIP1-1     DTLFSMALRFPAQALGAAGGAMAISELI----HPKYKHMIGGPSLKVDLHTGAVAELVLT
CaSIP1-2     DTLFSMALRFPAQALGAVGGAVAIMEVI----PPKYRHMIGGPALKVDLHTGAIAEGVLT
```

```
                   TM4                        TM5
CaPIP1-3  FVLVYTVFS-ATDAKRSARDSHVPILAPLPIGFAVFLVHLATI-----------------
CaPIP1-4  FILVYTVFS-ATDAKRSARDSHVPILAPLPIGFAVFLVHLATI-----------------
CaPIP1-1  FVLVYTVFS-ATDAKRNARDSHVPILAPLPIGFAVFLVHLATI-----------------
CaPIP1-2  FVLVYTVFS-ATDAKRNARDSHVPLLAPLPIGFAVFLVHLATI-----------------
CaPIP2-5  FVLVYTVFS-ATDPKRNARDSHVPVLAPLPIGFAVFMVHLATI-----------------
CaPIP2-2  FFLVYTVFS-ATDPKRNARDSHVPVLAPLPIGFAVFVVHLATI-----------------
CaPIP2-1  FVLVYTVFS-ATDPKRSARDSHVPVLAPLPIGFAVFMVHLATI-----------------
CaPIP2-3  FVLVYTVFS-ATDPKRNARDSHVPVLAPLPIGFAVFMVHLATI-----------------
CaPIP2-4  FVLVYTVFS-ATDPKRSARDSHVPVLAPLPIGFAVFMVHLATI-----------------
CaTIP5-1  FVLVYTIYA-ARDTRRGG----QQLSSTLVIGLISGASVLAAG-----------------
CaTIP4-1  FSLLFTVYATMVDPKKGV----RHGLGPTLVGFVVGANILA-G-----------------
CaTIP4-2  FSLLFTVYATMVDPKKGA----LNGLGPTLVGFVVGANILAGG-----------------
CaTIP3-1  FGLMYTVYATAIDPKRGT----VGTIAPLAIGLVVGANILAGG-----------------
CaTIP3-2  FGLMYTVYATAIDPKRGT----IGAIAPLAIGLIVGANILVGG-----------------
CaTIP2-3  FGLVYTVYATAADPKKGS----LGTIAPIAIGFIVGANILVAG-----------------
CaTIP2-1  FGLVYTVYATAADPKKGS----LGTIAPIAIGLSVGANILAAG-----------------
CaTIP2-2  FGLVYTVYATAADPKKGS----LGTIAPIAIGFIVGANILAAG-----------------
CaTIP1-1  FGLVYTVYATAVDPKKGN----IGIIAPIAIGFIVGANILVGG-----------------
CaTIP1-2  FGLVYTVYATAVDPKKGD----LGTIAPIAIGFIVGANILAGG-----------------
CaTIP1-3  FGLVYTVYATAIDPKKGN----LGVVAPLAIGCVVGANILVGG-----------------
CaTIP1-4  FGLVYTVYATAVDPKKGN----VGVVAPIAIGFIVGANILAGG-----------------
CaNIP4-1  FIIMFLVSALTSEHQS------VGHLSGFVAGIAIGLAVLITG-----------------
CaNIP2-1  FTMVLISTAVSTDPKA------IGELSGVAVGSSVCIASIVAG-----------------
CaNIP3-4  FILLFVVTAVATDTRA------VGELAGIAVGATVLLNILISG-----------------
CaNIP3-1  FNLMFVVTAVATDTRA------VGELAGIAVGATVMLNILIAG-----------------
CaNIP6-1  ----------------------VGELAGIAVGATVMLNILIAG-----------------
CaNIP3-2  FNLMFVVTAVATDTRV------MGEFAGITVEATVMLNILIAGGQNCIFIWNGGWEVKVG
CaNIP3-3  FNLMFVVTDVATDTRA------MGEFAGITVGATIMHNILIAG-----------------
CaNIP1-9  FFLMFVITAVATDDRA------IGELTGIAVGCTILIDILFAG-----------------
CaNIP1-6  FLLMFVISGVGTDNRA------VNELAALAVGSTVLLVVLFAG-----------------
CaNIP1-8  FLLMFVISGVGTDNRA------VNELAALAVGSTVLLVVLFAG-----------------
CaNIP1-5  FFLMFVISGVATDNRA------IGELAGIAIGSTLLLNAIIAA-----------------
CaNIP1-7  FYLMFVISGVATDNRA------IGELAGLAVGSTVLLNVMFAG-----------------
CaNIP1-3  FYLMFIISGVATDNRA------IGELAGIAVGSTVLLNVMFAG-----------------
CaNIP1-4  FYLMFIISGVATDNRA------IGELAGIAVGSTVLLNVMFAG-----------------
CaNIP1-2  FLLMFVVSAVSTDDRA------VNDMGGVAVGMTIMLNLFIAG-----------------
CaNIP1-1  FLLMFVISAVSTDNRA------IGELAGIAVGMTIMIDVFIAG-----------------
CaSIP2-1  YAIVTISLGLAATKIHG-----SFFMKTWISSLSKLTLHILGS-----------------
CaSIP1-1  FVITFIVLCIFLKGPR------NELMKIWLLAMSTVTLVMAGG-----------------
CaSIP1-2  FVITFAVLFIMLRGPR------SELVKTLLTAILTVALIIVGS-----------------
                                              :

                               LE
CaPIP1-3  ------------PITGTGINPARSLGAAIIFNKDLGWDDQWIFWVGPFIGAALAA-LYHQ
CaPIP1-4  ------------PITGTGINPARSLGAAIIFNKDLGWDDHWIFWVGPFIGAALAA-LYHQ
CaPIP1-1  ------------PITGTGINPARSLGAAIIYNREHAWDDQWIFWVGPFIGAALAA-LYHQ
CaPIP1-2  ------------PITGTGINPARSLGAAIIFNRDFAWDDHWIFWVGPFIGAALAA-MYHQ
CaPIP2-5  ------------PITGTGINPARSFGAAVIFNNAKVWDDHWIFWVGPFVGALAAA-AYHQ
CaPIP2-2  ------------PITGTGINPARSLGAAVIYNNHKAWDHHWIFWVGPFIGAAIAA-IYHQ
CaPIP2-1  ------------PVTGTGINPARSLGSAVILNQDKPWDDHWIFWVGPFAGAAIAA-FYHQ
CaPIP2-3  ------------PITGTGINPARSFGSAVIYNEGKIWDDQWIFWVGPIIGATVAA-IYHQ
CaPIP2-4  ------------PVTGTGINPARSFGPAVIYNNDKAWDDQWIYWVGPFIGAAIAA-FYHQ
CaTIP5-1  ------------PFSGGSINPACAFGSASIAGT---FRNQAVYWVGPLIGATVAGLLYDN
CaTIP4-1  ------------AFSGASINPARSFGPALVSGN---WTNHWVYWVGPLIGGGFAGFIYEN
CaTIP4-2  ------------AFSAASMNPARSFGPALVSGN---WTDHWVYWVGPLIGGGLAGFIYEN
CaTIP3-1  ------------PFDGACMNPARAFGPALVGWR---WHFHWIYWVGPLLGAAIAALLYEY
CaTIP3-2  ------------AFDGACMNPALAFGPSLVGWR---WHQHWIFWLGPFIGAALAAIIYEY
CaTIP2-3  ------------PFSGGSMNPARSFGPAVVSGN---FADNWIYWVGPLIGGGLAGLIYGD
```

```
                                         LE                      TM6
CaTIP2-1   -------------PFSGGSMNPARSFGPAVVSGD---FHDNWIYWVGPLIGGGLAGLIYTH
CaTIP2-2   -------------PFSGGSMNPARSFGPAVVSGN---FHDNWVYWVGPLVGGGLAGLIYDN
CaTIP1-1   -------------AFTGASMNPAVSFGPAVVSWS---WSNHWIYWAGPLIGGGLAGLIYEV
CaTIP1-2   -------------AFDGASMNPAVSFGPAVVSWT---WTNHWVYWVGPLIGSAVAAVVYEI
CaTIP1-3   -------------VFDGASMNPAVSFGPAVVSGV---WTHHWVYWVGPFIGSATAAILYDN
CaTIP1-4   -------------AFDGASMNPAVSFGPAVVSWT---WTHHWVYWVGPFTGAAIAAIIYDN
CaNIP4-1   -------------PVSGGSMNPARSLGPAIVSWK---FNYIWIYIIAPSSGAIAGALMFRF
CaNIP2-1   -------------PISGGSMNPARTLGPAIATSS---YKGIWVYMVGPITGALLGTWSYVV
CaNIP3-4   -------------PTSGGSMNPVRTLGPAVAAGN---YKHIWIYLVAPTLGALAGSGVYTL
CaNIP3-1   -------------PITGGSMNPVRSLGPAIAANN---YKAIWVYLLAPIIGALGGAGTYTA
CaNIP6-1   -------------PATGGSMNPVRTLGPTIATNN---FRGIWLYLIAPILGALTGAGAYTV
CaNIP3-2   DAWYISLSGGVWPATGGSMNPVRALGPAIAANN---FRDIWFYLIAPILGALIGAGAYIV
CaNIP3-3   -------------------------------------YVQRYYF-----------------
CaNIP1-9   -------------PITGASMNPARSLGPAVVDHE---YRGLWIYLISPILGALIGTWTYNF
CaNIP1-6   -------------PITGASMNPARSLGPAIVHHE---YRGIWIYLVSPILGALAGTWTYTF
CaNIP1-8   -------------PITGASMNPARSLGPAIVHHE---YRGIWIYLVSPILGALAGTLTYTF
CaNIP1-5   -------------PITGASMNPARSLGPAFVHNK---YRGIWIYLVSPILGAVAGAWVYNT
CaNIP1-7   -------------PITGASMNPARSLGPAIVHNE---YRGIWIYIVSTTLGAMAGTWVYNI
CaNIP1-3   -------------PITGASMNPARSIGPAFVHME---YNGIWIYLVSPILGAVAGAWVYNI
CaNIP1-4   -------------PITGASMNPARSIGPAFVHME---YNGIWIYLVSPILGAVAGAWVYNI
CaNIP1-2   -------------PVSGASMNPARSIGPALVKHI---YKGLWIYIVGPIVGAIAGAIAYNF
CaNIP1-1   -------------PISGASMNPARSFGPALVMHI---YDGFWIYIVGPFVGAILGASAYNL
CaSIP2-1   -------------DLTGGCMNPAAVMGWAYARGDHITKEHILVYWLAPIEATILAVWTFKL
CaSIP1-1   -------------AYTGPSMNPANAFGWAYINNRHNTCDQFYVYWICPFTGAILAAWLFRA
CaSIP1-2   -------------AYTGPSMNPVLV------------GDQVFCFHQ-----------LLSL
                                                   :

CaPIP1-3   VVIRAIPFKS------S--------------------------------------------
CaPIP1-4   VVIRAIPFKS------K--------------------------------------------
CaPIP1-1   IIIRAIPFKA------RG-------------------------------------------
CaPIP1-2   IVIRAIPFKT------RA-------------------------------------------
CaPIP2-5   YILRAAAIKA------LGSFRSNPTN-----------------------------------
CaPIP2-2   FVLRAQAAKA------LGSFKSSSNL-----------------------------------
CaPIP2-1   FILRAGAVKA------LGSFRSNPTV-----------------------------------
CaPIP2-3   YILRGSAIKA------LGSFRSNA-------------------------------------
CaPIP2-4   FILRATAIKA------LGSFRSNP-------------------------------------
CaTIP5-1   VLFPSQSLDS------IRGVSEVNVRV----------------------------------
CaTIP4-1   FFI----------------------------------------------------------
CaTIP4-2   FFINRDHVPL------VVDEESY--------------------------------------
CaTIP3-1   VILPTVAPNP------NPHPHPHHQPLAPEDY-----------------------------
CaTIP3-2   VIIPTEPPHA------HQPLAPEDY------------------------------------
CaTIP2-3   IFIGSYAPAP------ASETYP---------------------------------------
CaTIP2-1   LFIPSKHQQP------MPK------------------------------------------
CaTIP2-2   VFLRSEHAPL------ASDY-----------------------------------------
CaTIP1-1   VFISHTHEQ-------LPTTDY---------------------------------------
CaTIP1-2   FFITPSSYEQ------LPVADY---------------------------------------
CaTIP1-3   IFIGDDAHQP------LSNSDF---------------------------------------
CaTIP1-4   IFIGDDGHEP------LSDF-----------------------------------------
CaNIP4-1   LRLQDQQCTS---SNITNVGHPIPFCARRSGSMILLVEKNWSLFSNRVEGFRQRYVLRTE
CaNIP2-1   IQETNKQALT-----TSLKLHHEMKGIELVGDKDNQCSV----------------------
CaNIP3-4   VKLRDNEANP------AQSVRSFRR------------------------------------
CaNIP3-1   VKLPQEDDNA------KANAPSNPASFRR--------------------------------
CaNIP6-1   VKLPNEEFNP------ELKASSAPGSFRR--------------------------------
CaNIP3-2   VKLPDEEFN----------------------------------------------------
CaNIP3-3   -------------------------------------------------------------
CaNIP1-9   IRHKNKPMCDELTKIVPTKIVPFFRSSRM--------------------------------
CaNIP1-6   IRYTNKPAT-------IPKSASFLKGA----------------------------------
```

Supplementary File S2: **Multiple sequence alignment of deduced amino acid sequences of 40 CaAQP genes**. Sequence alignment was performed using CLUSATLW. Transmembrane helices (TM1–TM6) and two NPAs (LB and LE) are highlighted in gray colour, P1–P5 residues highlighted in blue colour, and ar/R selectivity filter residues highlighted in green colour. The NPA motifs are shown in red color.
