# Supplementary material for: Genome-Wide Analysis of the Aquaporin Gene Family in Chickpea (Cicer arietinum L.)
Source: Front Plant Sci. 2016 Nov 29;7:1802. doi: 10.3389/fpls.2016.01802 (PMC5126082; doi:10.3389/fpls.2016.01802)
Supplement: Supplementary File S4 — Summary of specificity-determining positions (SDP) identified in the CaAQPs. SDP positions identified in the present study and not found conserved with the reported SDPs in AQPs are highlighted in red color. [file DataSheet4.PDF]

Supplementary File 3: **Summary of specificity-determining positions (SDP) identified in the CaAQPs.** SDP positions identified in the present study and not found conserved with the reported SDPs in AQPs are highlighted in red colour.

	SDP1	SDP2	SDP3	SDP4	SDP5	SDP6	SDP7	SDP8	SDP9
**Boric acid transporter**	**T/V**	**I/V**	**H/I**	**P**	**E**	**I/L**	**I/L/T**	**A/T**	**A/G/K/P**
**CaPIP1-1**	T	I	H	P	E	L	L	T	P
**CaPIP1-2**	T	I	H	P	E	L	L	T	P
**CaPIP1-3**	T	I	H	P	E	L	L	T	P
**CaPIP1-4**	T	I	H	P	E	L	L	T	P
**CaPIP2-1**	T	I	H	P	E	M	L	T	P
**CaPIP2-2**	T	I	H	P	E	V	L	T	P
**CO_2**	**I/L/V**	**I/V**	**C**	**A**	**I/V**	**D/S**	**W**	**D**	**W**
**CaPIP1-1**	V	I	C	A	I	E	W	D	W
**CaPIP1-2**	I	I	C	A	L	D	W	D	W
**CaPIP1-3**	V	I	C	A	I	D	W	D	W
**CaPIP1-4**	L	M	C	A	I	D	W	D	W
**CaPIP2-1**	I	I	C	A	V	D	W	D	W
**CaPIP2-2**	I	M	C	A	V	H	W	H	W
**H_2O_2**	**A/S**	**A/G**	**L/V**	**A/F/L/T/V**	**I/L/V**	**H/I/L/Q**	**F/Y**	**A/V**	**P**
**CaPIP1-1**	A	G	V	F	I	Q	F	V	P
**CaPIP1-2**	A	G	V	F	I	H	F	V	P
**CaPIP1-3**	A	G	V	F	I	Q	F	V	P
**CaPIP1-4**	A	G	V	F	I	H	F	V	P
**CaPIP2-1**	A	G	V	F	I	H	F	V	P
**CaPIP2-2**	A	G	V	F	I	H	F	V	P
**CaPIP2-3**	A	G	A	F	I	Q	F	V	P
**CaPIP2-4**	A	G	A	F	I	Q	Y	V	P
**CaPIP2-5**	A	G	V	L	I	H	F	V	P
**CaTIP1-1**	S	A	L	A	I	H	Y	A	P
**CaTIP1-2**	S	A	L	A	I	H	Y	V	P
**CaTIP1-3**	S	A	L	C	I	H	Y	V	P
**CaTIP1-4**	S	A	L	A	I	H	Y	V	P
**CaTIP2-1**	S	A	L	V	I	N	Y	V	P
**CaTIP2-2**	S	A	L	V	I	N	Y	V	P
**CaTIP2-3**	S	A	L	V	I	N	Y	V	P

**CaNIP1-5**	A	G	L	I	I	I	Y	V	P
**CaNIP1-6**	S	A	L	I	V	I	Y	V	P
**CaNIP1-7**	S	A	L	I	V	I	Y	V	P
**CaNIP1-8**	S	A	L	I	V	I	Y	V	P
**CaNIP1-9**	S	A	L	I	V	L	Y	I	P
**Urea Transporters**	**H**	**P**	**F/I/L/T**	**A/C/F/L**	**L/M**	**A/G/P**	**G/S**	**G/S**	**N**
**CaPIP1-1**	H	P	F	F	L	P	G	G	N
**CaPIP1-2**	H	P	F	F	L	P	G	G	N
**CaPIP1-3**	H	P	F	F	L	P	G	G	N
**CaPIP1-4**	H	P	F	F	L	P	G	G	N
**CaPIP2-1**	H	P	F	F	L	P	G	G	N
**CaPIP2-2**	H	P	F	F	L	P	G	G	N
**CaPIP2-3**	H	P	F	F	L	P	G	G	N
**CaPIP2-4**	H	P	F	F	L	P	G	G	N
**CaPIP2-5**	H	P	F	F	L	P	G	G	N
**CaTIP1-1**	H	P	F	F	L	A	G	S	N
**CaTIP1-2**	H	P	F	F	L	A	G	S	N
**CaTIP1-3**	H	P	F	F	L	V	G	S	N
**CaTIP1-4**	H	P	F	F	L	A	G	S	N
**CaTIP2-1**	H	P	F	A	L	P	G	S	N
**CaTIP2-3**	H	P	F	A	L	P	G	S	N
**CaTIP2-2**	H	P	F	A	L	P	G	S	N
**CaTIP5-1**	H	P	F	A	L	P	G	S	N
**CaNIP1-5**	H	P	L	A	L	P	G	S	N
**CaNIP1-6**	H	P	I	A	L	P	G	S	N
**CaNIP1-7**	H	P	I	A	L	P	G	S	N
**CaNIP1-8**	H	P	I	A	L	P	G	S	N
**CaNIP1-9**	H	P	I	A	L	P	G	S	N
**CaNIP3-1**	H	P	I	A	L	P	G	S	N
