# Supplementary material for: Genome-Wide Analysis of the Aquaporin Gene Family in Chickpea (Cicer arietinum L.)
Source: Front Plant Sci. 2016 Nov 29;7:1802. doi: 10.3389/fpls.2016.01802 (PMC5126082; doi:10.3389/fpls.2016.01802)
Supplement: Supplementary File S5 — Predicted 3D structure of the 40 CaAQP protein generated using Phyre 2 server. [file DataSheet5.PDF]

Supplementary File S4: **Predicted 3D structure of the 40 CaAQP protein generated using Phyre 2 server.**

CaAQP Gene ID	Hit	Confidence (%)	Alignment coverage (%)	3D Image
**CaNIP1-8**	**c2w2eA**	**100**	**97**	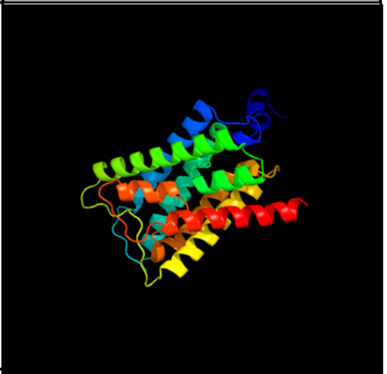
**CaTIP4-1**	**c5i32A**	**100**	**96**	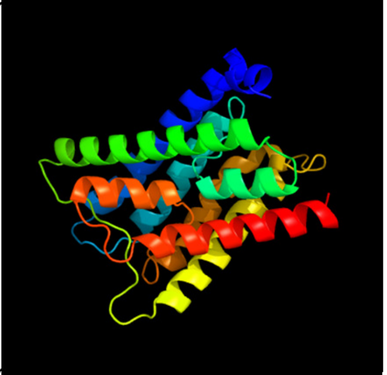
**CaNIP1-7**	**c2w2eA**	**100**	**92**	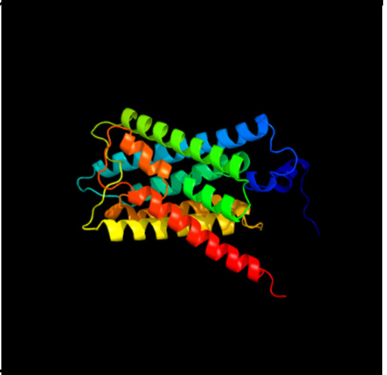
**CaNIP6-1**	**c2w2eA**	**100**	**72**	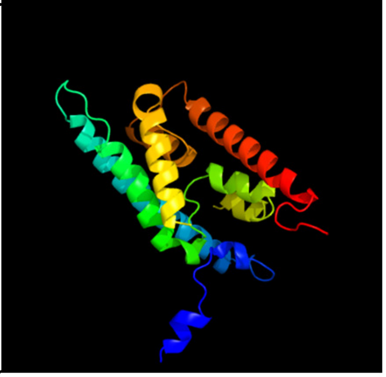

CaPIP2-3	c2w2eA	100	91	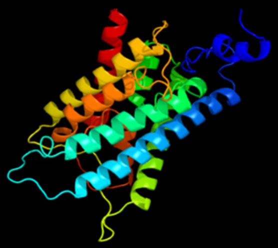
CaTIP1-4	c5i32A	100	93	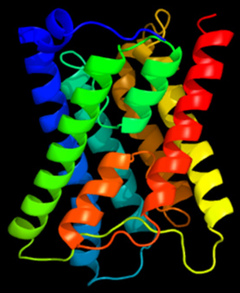
CaNIP3-1	c2w2eA	100	80	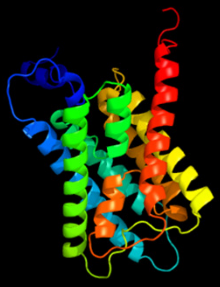
CaTIP3-1	c5i32A	100	88	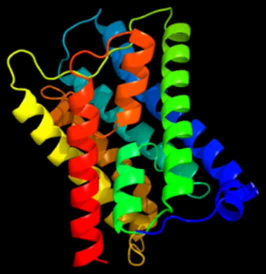

**CaNIP1-1**	**c2w2eA**	**100**	**90**	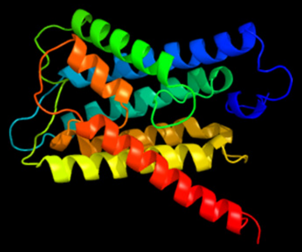
**CaTIP2-2**	**c5i32A**	**100**	**94**	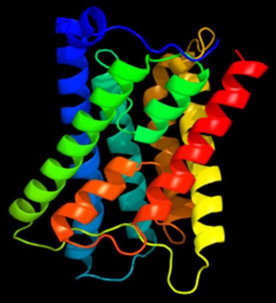
**CaPIP2-2**	**c2w2eA**	**100**	**93**	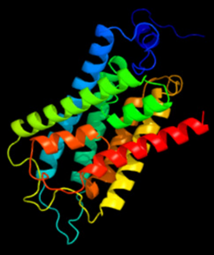
**CaTIP1-1**	**c5i32A**	**100**	**93**	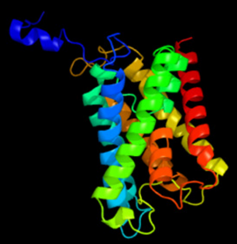

CaPIP1-2	c2w2eA	100	88	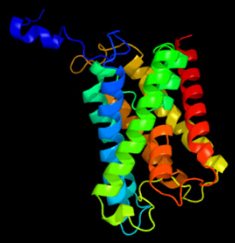
CaNIP4-1	c2w2eA	100	73	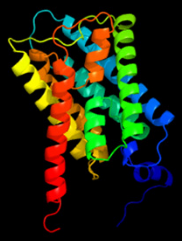
CaPIP2-5	c2w2eA	100	92	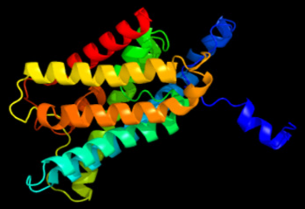
CaPIP2-1	c2w2eA	100	98	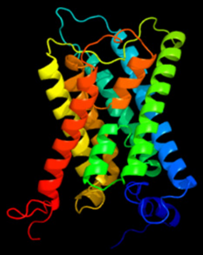

**CaNIP1-5**	**c2w2eA**	**100**	**88**	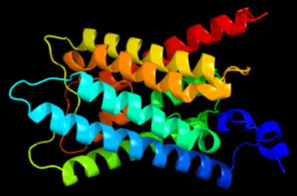
**CaNIP3-3**	**c2w2eA**	**100**	**94**	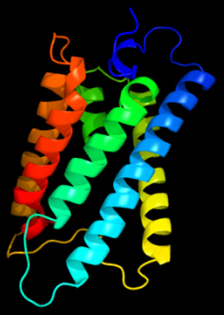
**CaTIP4-2**	**c5i32A**	**100**	**93**	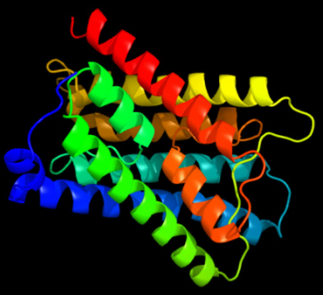
**CaTIP2-3**	**c5i32A**	**100**	**93**	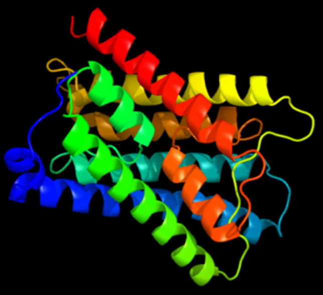

CaTIP3-2	c5i32A	100	91	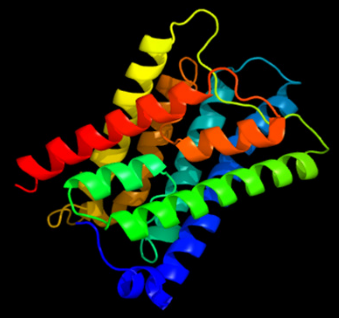
CaPIP1-3	c2w2eA	100	88	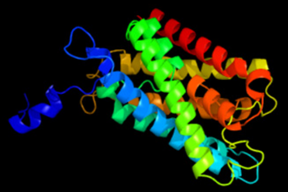
CaPIP1-1	c2w2eA	100	93	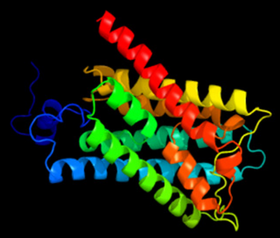
CaNIP3-4	c2w2eA	100	87	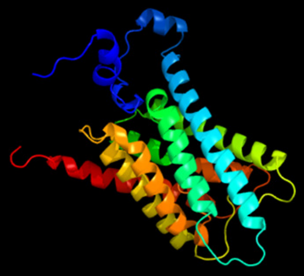

**CaTIP2-1**	**c5i32A**	**100**	**95**	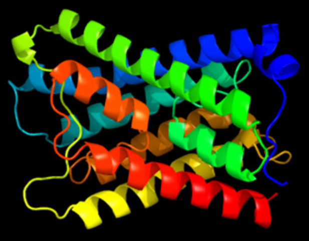
**CaTIP5-1**	**d1j4na**	**100**	**96**	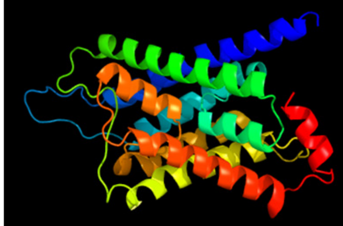
**CaSIP2-1**	**c2b6pA**	**100**	**96**	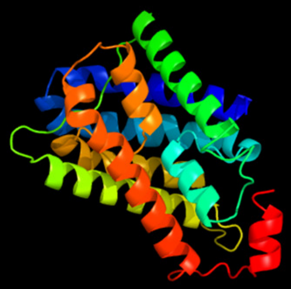
**CaSIP1-2**	**c2f2bA**	**100**	**98**	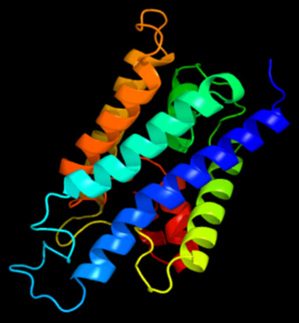

CaTIP1-2	c5i32A	100	92	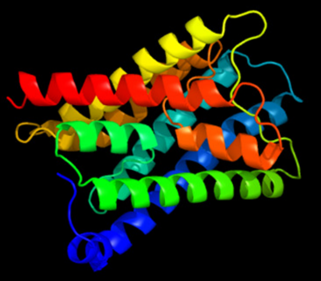
CaNIP1-9	c2w2eA	100	90	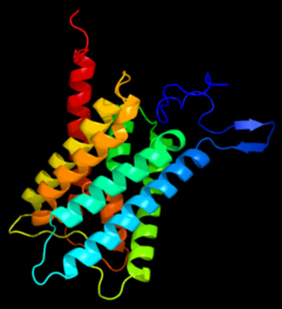
CaNIP1-6	c2w2eA	100	92	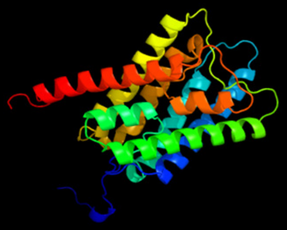
CaSIP1-1	c2f2bA	100	95	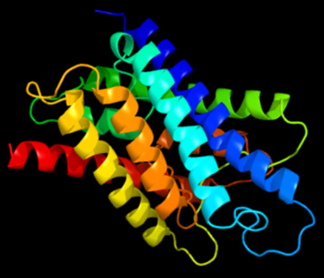

**CaPIP1-4**	**c2w2eA**	**100**	**89**	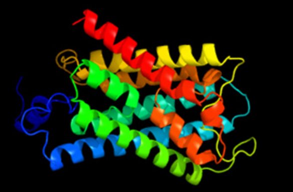
**CaNIP1-2**	**c2w2eA**	**100**	**92**	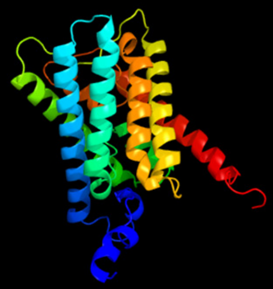
**CaNIP1-3**	**c2w2eA**	**100**	**90**	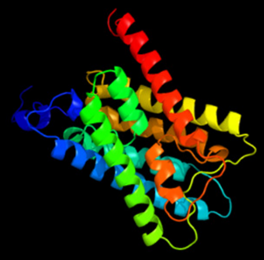
**CaNIP2-1**	**c2b6pA**	**100**	**90**	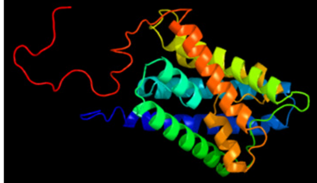

**CaNIP3-2**	**c2b6pA**	**100**	**98**	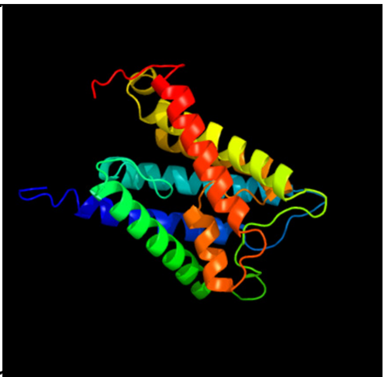
**CaNIP1-4**	**c2w2eA**	**100**	**90**	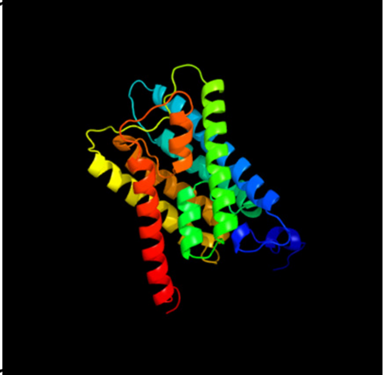
**CaTIP1-3**	**c5i32A**	**100**	**91**	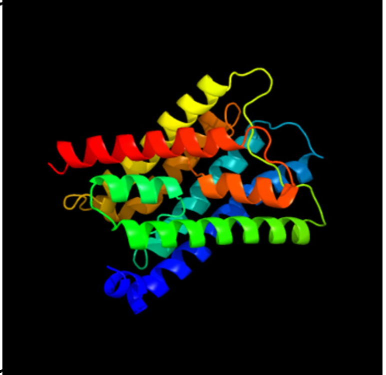
**CaPIP2-4**	**c2w2eA**	**100**	**98**	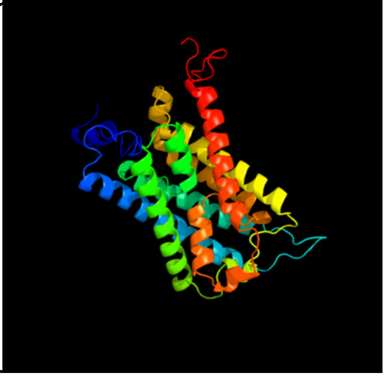
